# Effects of shinbuto and ninjinto on prostaglandin E_2_ production in lipopolysaccharide-treated human gingival fibroblasts

**DOI:** 10.7717/peerj.4120

**Published:** 2017-12-01

**Authors:** Toshiaki Ara, Norio Sogawa

**Affiliations:** Department of Pharmacology, Matsumoto Dental University, Shiojiri, Nagano, Japan

**Keywords:** Shokyo, Kankyo, Kampo medicine, Herb, Human gingival fibroblast, Anti-inflammatory effect, Arachidonic acid cascade, Prostaglandin E2

## Abstract

Previously, we revealed that several kampo medicines used for patients with excess and/or medium patterns (kakkonto (TJ-1), shosaikoto (TJ-9), hangeshashinto (TJ-14), and orento (TJ-120)) reduced prostaglandin (PG)E_2_ levels using LPS-treated human gingival fibroblasts (HGFs). Recently, we examined other kampo medicines used for patients with the deficiency pattern [bakumondoto (TJ-29), shinbuto (TJ-30), ninjinto (TJ-32), and hochuekkito (TJ-41)] and the herbs comprising shinbuto and ninjinto using the same experimental model. Shinbuto and ninjinto concentration-dependently reduced LPS-induced PGE_2_ production by HGFs, whereas hochuekkito weakly reduced and bakumondoto did not reduce PGE_2_ production. Shinbuto and ninjinto did not alter cyclooxygenase (COX) activity or the expression of molecules involved in the arachidonic acid cascade. Therefore, we next examined which herbs compromising shinbuto and ninjinto reduce LPS-induced PGE_2_ production. Among these herbs, shokyo (*Zingiberis Rhizoma*) and kankyo (*Zingiberis Processum Rhizoma*) strongly and concentration-dependently decreased LPS-induced PGE_2_ production. However, both shokyo and kankyo increased the expression of cytosolic phospholipase (cPL)A_2_ but did not affect annexin1 or COX-2 expression. These results suggest that shokyo and kankyo suppress cPLA_2_ activity. We demonstrated that kampo medicines suppress inflammatory responses in patients with the deficiency pattern, and in those with excess or medium patterns. Moreover, kampo medicines that contain shokyo or kankyo are considered to be effective for the treatment of inflammatory diseases.

## Introduction

Periodontal disease is an inflammatory disease of the gingiva that destroys periodontal tissues. In severe cases, alveolar bone is absorbed. In inflammatory responses and tissue degradation, prostaglandin E_2_ (PGE_2_), interleukin (IL)-6, and IL-8 play important roles. As PGE_2_ has several functions in vasodilation, the enhancement of vascular permeability and pain, and osteoclastogenesis induction, PGE_2_ participates in inflammatory responses and alveolar bone resorption in periodontal disease (Noguchi & Ishikawa, 2007).

Previously, we reported that several kampo medicines, shosaikoto (TJ-9) (Ara et al., 2008b), orento (TJ-120) (Ara et al., 2010), hangeshashinto (TJ-14) (Nakazono et al., 2010), and kakkonto (TJ-1) (Kitamura, Urano & Ara, 2014), suppress lipopolysaccharide (LPS)-induced PGE_2_ production by human gingival fibroblasts (HGFs). Moreover, we found that shokyo, kanzo, and keihi, which are herbs contained in kakkonto, reduce PGE_2_ production (Ara & Sogawa, 2016). These results suggested that these kampo medicines and herbs have anti-inflammatory effects in periodontal disease.

However, these kampo medicines are used for patients with the excess pattern or medium pattern. Kampo medicine used for those with the deficiency pattern remains to be elucidated. In the present study, we therefore examined the anti-inflammatory effects of the kampo medicines for patients with the deficiency pattern [bakumondoto (TJ-29), shinbuto (TJ-30), ninjinto (TJ-32), and hochuekkito (TJ-41)], which are used for the treatment of inflammatory diseases. Furthermore, we examined the effects on PGE_2_ production using herbs comprising the kampo medicines that reduce PGE_2_ production.

## Materials and Methods

### Reagents

Kampo medicines (bakumondoto, shinbuto, ninjinto, and hochuekkito) were purchased from Tsumura & Co. (Tokyo, Japan). Powders of 8 herbs (bukuryo, bushi, kankyo, kanzo, ninjin, shakuyaku, shokyo, and sojutsu) were provided by Tsumura & Co. The ingredients in shinbuto and ninjinto formulas are shown in Tables 1 and 2. Powders of kampo medicines or herbs were suspended in Dulbecco’s modified Eagle’s medium (D-MEM; Sigma, St. Louis, MO, USA) containing 10% heat-inactivated fetal calf serum, 100 units/ml penicillin, and 100 mg/ml streptomycin (culture medium), and were rotated at 4 °C overnight. Then, the suspensions were centrifuged and the supernatants were filtrated through a 0.45 µm-pore membrane. Lipopolysaccharide (LPS) from *Porphyromonas gingivalis* 381 was provided by Professor Nobuhiro Hanada (School of Dental Medicine, Tsurumi University, Japan). Arachidonic acid was purchased from Cayman Chemical (Ann Arbor, MI). Other reagents were purchased from Nacalai tesque (Kyoto, Japan).

### Cells

HGFs were prepared as described previously (Nakazono et al., 2010). In brief, HGFs were prepared from free gingiva during the extraction of an impacted tooth with the informed consent of the subjects who consulted Matsumoto Dental University Hospital. The free gingival tissues were cut into pieces and seeded onto 24-well plates (AGC Techno Glass Co., Chiba, Japan). HGFs were maintained in culture medium at 37 °C in a humidified atmosphere of 5% CO_2_. For passage, HGFs were trypsinized, suspended, and plated into new cultures in a 1:3 dilution ratio. HGFs were used between the 10th to 15th passages in the assays. This study was approved by the Ethical Committee of Matsumoto Dental University (No. 0063).

### Measurement of cell viability

The numbers of cells were measured using WST-8 (Cell Counting Kit-8; Dojindo, Kumamoto, Japan) according to the manufacturer’s instructions. In brief, the media were removed by aspiration and the cells were treated with a 100-µl mixture of WST-8 with culture medium for 2 h at 37 °C in CO_2_ incubator. Optical density was measured (measured wavelength at 450 nm and reference wavelength at 655 nm) using an iMark microplate reader (Bio-Rad, Hercules, CA, USA), and the mean background value was subtracted from each value. Data is represented as means ± S.D. (*n* = 4).

**Table 1:** The ingredients in the shinbuto formula.

Japanese name	Latin name	Amount (g)	Amount (g/g of product)^*^
bukuryo	*Poria Sclerotium*	4.0	0.089
shakuyaku	*Paeoniae Radix*	3.0	0.067
sojutsu	*Atractylodis Lanceae Rhizoma*	3.0	0.067
shokyo	*Zingiberis Rhizoma*	1.5	0.033
bushi	*Processi Aconiti Radix*	0.5	0.011
Total		12.0	0.267

**Notes.**

* 7.5 g of shinbuto product contains 2.0 g of a dried extract of the mixed crude drugs.

**Table 2:** The ingredients in the ninjinto formula.

Japanese name	Latin name	Amount (g)	Amount (g/g of product)^*^
kankyo	*Zingiberis Processum Rhizoma*	3.0	0.083
kanzo	*Glycyrrhizae Radix*	3.0	0.083
sojutsu	*Atractylodis Lanceae Rhizoma*	3.0	0.083
ninjin	*Ginseng Radix*	3.0	0.083
Total		12.0	0.333

**Notes.**

* 7.5 g of ninjinto product contains 2.5 g of a dried extract of the mixed crude drugs.

### Measurement of prostaglandin E_2_ (PGE_2_), interleukin (IL)-6, and IL-8

HGFs were seeded in 96-well plates (10,000 cells/well) and incubated in serum-containing medium at 37 °C overnight. Then, the cells were treated with varying concentrations of each kampo medicine (0, 0.01, 0.1, or 1 mg/ml) or each herb (0, 10, 30, or 100 µg/ml) in the absence or presence of LPS (10 ng/ml) for 24 h (200 µl per well) in triplicate or quadruplicate for each sample. After the culture supernatants were collected, viable cell numbers were measured using WST-8 as described above.

The concentrations of PGE_2_, IL-6, and IL-8 in the culture supernatants were measured by enzyme-linked immunosorbent assay (ELISA) according to the manufacturer’s instructions (PGE_2_), Cayman Chemical; IL-6 and IL-8, Thermo Fisher Scientific Inc., Camarillo, MA, USA), and were adjusted by the number of viable cells. Data are represented as pg or ng per 10,000 cells (mean ± S.D.).

### Measurement of cyclooxygenase (COX)-2 activity

COX-2 activity was evaluated as reported previously (Wilborn et al., 1995) with slight modification. In brief, to estimate COX-2 activity, HGFs were treated with LPS and herbs for 8 h, washed, and incubated in culture medium containing exogenous arachidonic acid (10 µM). The concentrations of PGE_2_ in the supernatants were measured by ELISA. Data are represented as pg per 10,000 cells (mean ± S.D.).

### Preparation of cell lysates

HGFs were cultured in 60-mm dishes and treated with combinations of LPS and herbs for the indicated times. Then, cells were washed twice with Tris-buffered saline, transferred into microcentrifuge tubes, and centrifuged at 6,000 × g for 5 min at 4 °C. Supernatants were aspirated and cells were lysed on ice in lysis buffer (50 mM Tris–HCl, pH 7.4, 1% Nonidet P-40, 0.25% sodium deoxycholate, 150 mM NaCl, 1 mM ethyleneglycol bis(2-aminoethylether)tetraacetic acid (EGTA), 1 mM sodium orthovanadate, 10 mM sodium fluoride, 1/100 volume of protease inhibitor cocktail (Nacalai tesque)) for 30 min at 4 °C. Samples were next centrifuged at 12,000 × g for 15 min at 4 °C, and supernatants were collected. The protein concentration was measured using a BCA Protein Assay Reagent kit (Pierce Chemical Co., Rockford, IL, USA).

### Western blotting

The samples (10 µg of protein) were fractionated in a polyacrylamide gel under reducing conditions and transferred onto a polyvinylidene difluoride (PVDF) membrane (Hybond-P; GE Healthcare, Uppsala, Sweden). The membranes were blocked with 5% ovalbumin for 1 h at room temperature and incubated with primary antibody for an additional 1 h. The membranes were further incubated with horseradish peroxidase-conjugated secondary antibodies for 1 h at room temperature. Protein bands were visualized with an ECL kit (GE Healthcare). Densitometric values of each band were calculated using ImageJ software.

Antibodies against COX-2 (sc-1745, 1:500 dilution), cytosolic PLA_2_ (cPLA_2_) (sc-438, 1:200 dilution), annexin1 (sc-11387, 1:1,000 dilution), and actin (sc-1616, 1:1,000 dilution), which detects a broad range of actin isoforms, were purchased from Santa Cruz Biotechnology (Santa Cruz, CA). Antibodies against extracellular signal-regulated kinase (ERK; p44/42 MAP kinase antibody, 1:1,000 dilution) and phosphorylated ERK [Phospho-p44/42 MAPK (Thr202/Tyr204) (E10) monoclonal antibody, 1:2,000 dilution] were from Cell Signaling Technology (Danvers, MA). Horseradish peroxidase-conjugated anti-goat IgG (sc-2020, 1:20,000 dilution) was from Santa Cruz, and anti-rabbit IgG (1:20,000 dilution) and anti-mouse IgG (1:20,000 dilution) were from DakoCytomation (Glostrup, Denmark).

### Statistical analysis

**Figure 1:**
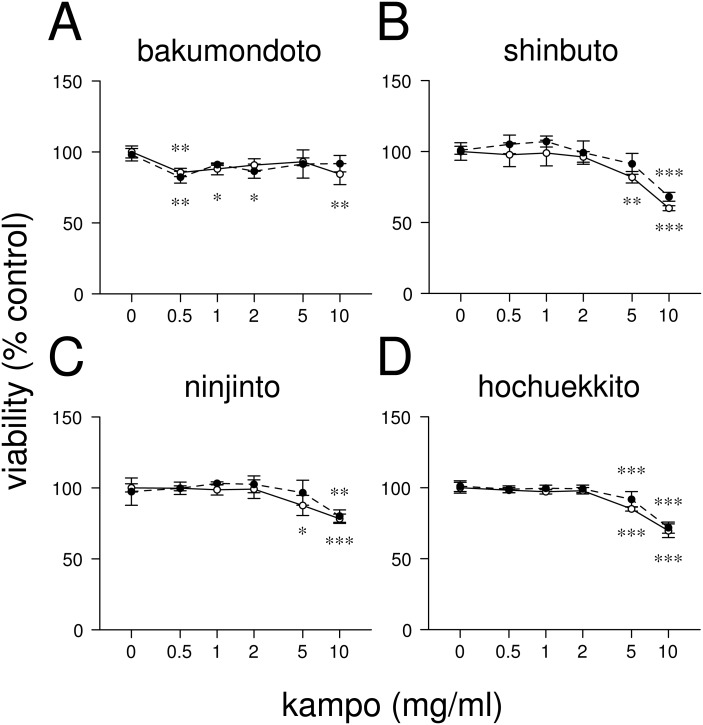
Effects of kampo medicines on cytotoxicity. HGFs were treated with combinations of LPS (0 or 10 ng/ml) and kampo medicine (0, 0.5, 1, 2, 5, or 10 mg/ml) for 24 h. Then, the numbers of viable cells were measured with WST-8. Open circles, treatment without LPS; closed circles, treatment with 10 ng/ml of LPS. ^∗^*P* < 0.05, ^∗∗^*P* < 0.01, ^∗∗∗^*P* < 0.001 (without vs. with kampo medicine). *P* values were calculated by pairwise comparisons and corrected with the Holm method (10 null hypotheses).

**Figure 2:**
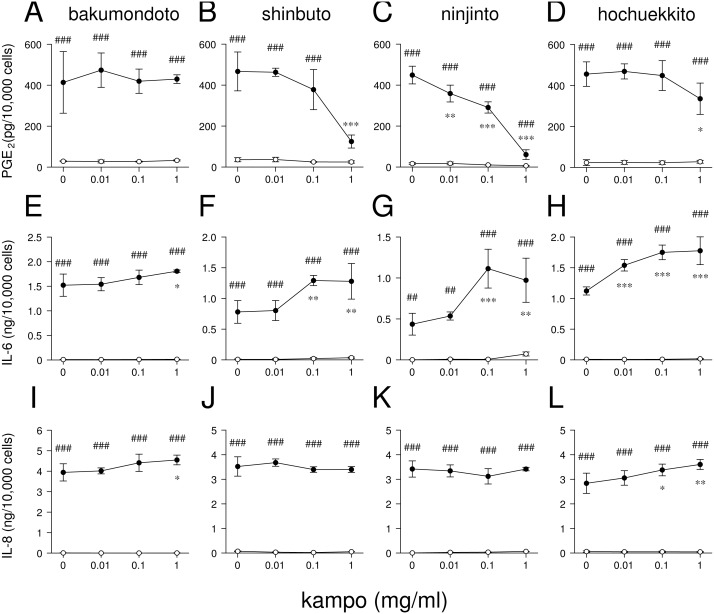
Effects of kampo medicines on PGE_2_, IL-6, and IL-8 production. HGFs were treated with combinations of LPS (0 or 10 ng/ml) and kampo medicine (0, 0.01, 0.1, or 1 mg/ml) for 24 h. Concentrations of PGE_2_ (A–D), IL-6 (E–H), and IL-8 (I–L) were measured by ELISA, adjusted by cell number, and expressed as per 10,000 cells (mean ± S.D., *n* = 3). Open circles, treatment without LPS; closed circles, treatment with 10 ng/ml of LPS. ^∗^*P* < 0.05, ^∗∗^*P* < 0.01, ^∗∗∗^*P* < 0.001 (without vs. with kampo medicine). ^#^*P* < 0.05, ^##^*P* < 0.01, ^###^*P* < 0.001 (without LPS vs. with LPS). *P* values were calculated by pairwise comparisons and corrected with the Holm method (10 null hypotheses).

Differences between groups were evaluated by the two-tailed pairwise comparison test with a pooled variance, followed by correction with the Holm method (total 10 null hypotheses; five null hypotheses without kampo vs. with kampo in the absence or presence of LPS in Fig. 1, total 10 null hypotheses; three null hypotheses without kampo vs. with kampo in the absence of LPS, three null hypotheses without kampo vs. with kampo in the presence of LPS, and four null hypotheses without LPS vs. with LPS in Fig. 2). Differences between the control group and experimental groups were evaluated by a two-tailed Dunnett’s test.

All computations were performed with the statistical program R (R Core Team, 2017). Dunnett’s test was performed using the ‘glht’ function in the ‘multcomp’ package. Values with *P* < 0.05 were considered significantly different.

## Results

### Effects of kampo medicines on HGFs viability

First, we examined the effects of four kampo medicines (bakumondoto, shinbuto, ninjinto, and hochuekkito) on HGFs viability. Bakumondoto did not affect the viability up to 10 mg/ml at 24 h treatment (Fig. 1A). In contrast, Shinbuto, ninjinto, and hochuekkito did not affect the viability up to 2 mg/ml, but decreased at 5 mg/ml and 10 mg/ml (Figs. 1B–1C). Therefore, up to 1 mg/ml of kampo medicines was used in further experiments because we used the same concentration of kampo medicines in previous studies (Ara et al., 2008b; Ara et al., 2010; Nakazono et al., 2010; Kitamura, Urano & Ara, 2014).

### Effects of kampo medicines on prostaglandin (PG)E_2_, interleukin (IL)-6, and IL-8 production

We examined whether these kampo medicines affected the production of PGE_2_ and inflammatory cytokines (IL-6 and IL-8) by HGFs. The concentrations of PGE_2_, IL-6, and IL-8 were adjusted according to viable cell number. HGFs treated with 10 ng/ml of LPS produced large amounts of PGE_2_, IL-6, and IL-8. Shinbuto and ninjinto strongly and concentration-dependently reduced LPS-induced PGE_2_ production (Figs. 2B–2C). In contrast, bakumondoto and hochuekkito had no or little effect on PGE_2_ production. Bakumondoto weakly, and shinbuto, ninjinto, and hochuekkito strongly increased LPS-induced IL-6 production (Figs. 2E–2H). Bakumondoto and hochuekkito weakly increased LPS-induced IL-8 production, but shinbuto and ninjinto did not affect IL-8 production (Figs. 2I–2L).

From these results, we selected two kampo medicines, shinbuto and ninjinto, which decreased PGE_2_ production and used them in the following experiments.

### Effects of shinbuto and ninjinto on the arachidonic acid cascade

**Figure 3:**
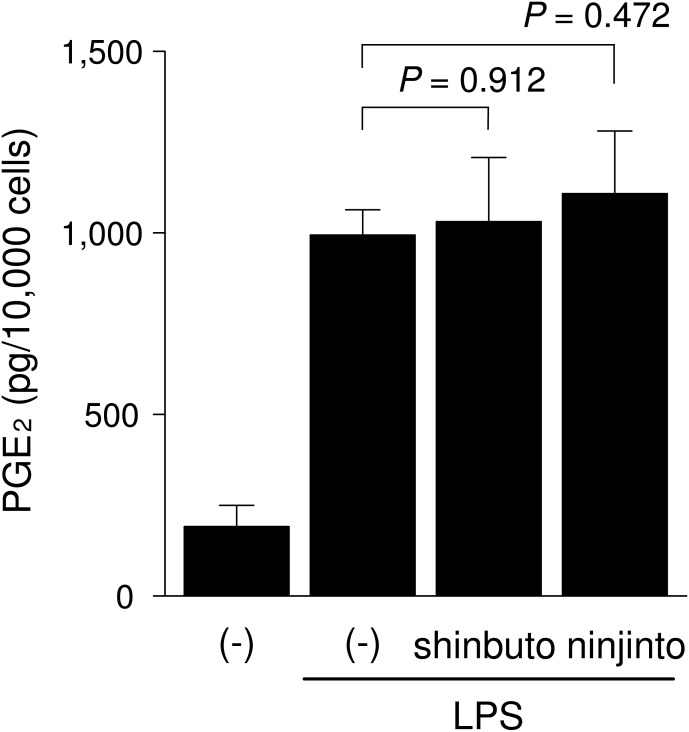
Effects of kampo medicines on COX activity. HGFs were treated with LPS (10 ng/ml) and kampo medicine (1 mg/ml) for 8 h, washed, and then treated with 10 µM arachidonic acid for 30 min. Concentrations of PGE_2_ were measured by ELISA, adjusted by cell number, and expressed as per 10,000 cells (mean ± S.D., *n* = 4). *P* values by Dunnett’s test are indicated.

To clarify the mechanism of how shinbuto and ninjinto reduced LPS-induced PGE_2_ production more directly, we examined the effects of these two kampo medicines on the arachidonic acid cascade. First, we examined the effects of shinbuto and ninjinto on COX activity. In order to bypass PLA_2_, we added exogenous arachidonic acid to HGFs treated with LPS alone or LPS plus kampo medicine (shinbuto or ninjinto). Then, we measured the PGE_2_ level produced by COX. However, shinbuto and ninjinto did not affect LPS-induced PGE_2_ production (Fig. 3).

**Figure 4:**
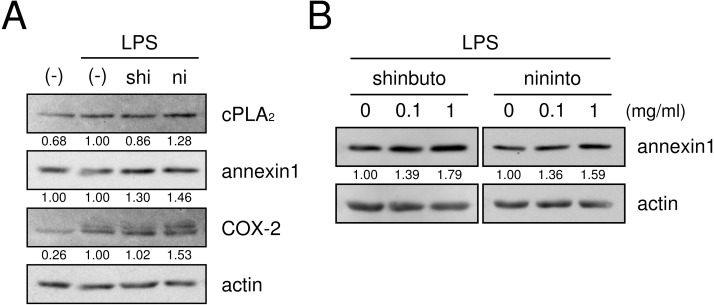
Effects of kampo medicines on cPLA_2_, annexin1, and COX-2 expression. HGFs were treated with a combination of LPS (0 or 10 ng/ml) and kampo medicines (0 or 1 mg/ml) for 8 h, and protein levels were examined by Western blotting. The band densities were normalized against LPS alone and actin, and indicated below each band. shi, shinbuto; ni, ninjinto.

Next, we examined whether shinbuto and ninjinto affected the expression of molecules in the arachidonic acid cascade. cPLA_2_, which is the most upstream enzyme in the arachidonic acid cascade, releases arachidonic acid from plasma membranes. Shinbuto slightly reduced cPLA_2_ expression and ninjinto slightly increased cPLA_2_ expression (Fig. 4A). COX-2 was weakly expressed in the absence of LPS, and the treatment with LPS alone increased COX-2 expression. However, shokyo did not alter but kankyo slightly increased LPS-induced COX-2 expression (Fig. 4). Annexin1 (also named lipocortin1) is produced by glucocorticoids and inhibits cPLA_2_ activity (Gupta et al., 1984; Wallner et al., 1986). Shinbuto and ninjinto slightly increased annexin1 expression (Fig. 4A) in a concentration-dependent manner (Fig. 4B).

**Figure 5:**
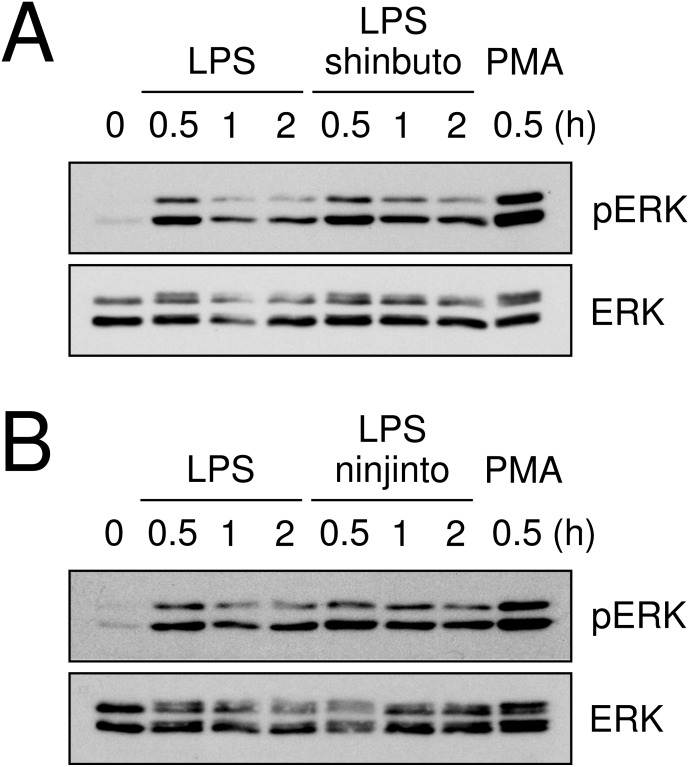
Effects of kampo medicines on LPS-induced ERK phosphorylation. HGFs were untreated (0 h), treated with LPS (10 ng/ml), or treated with both LPS and kampo medicines (1 mg/ml) for 0.5, 1, or 2 h. PMA was used as a positive control. Western blotting was performed using anti-phosphorylated ERK or anti-ERK antibodies. pERK, phosphorylated ERK. The upper band indicates ERK1 (p44 MAPK) and lower band ERK2 (p42 MAPK).

Lastly, we evaluated the effects of shinbuto and ninjinto on ERK phosphorylation. cPLA_2_ is directly phosphorylated and activated by phosphorylated ERK (Lin et al., 1993; Gijón et al., 1999). Therefore, we examined whether shinbuto and ninjinto suppressed LPS-induced ERK phosphorylation. LPS treatment enhanced ERK phosphorylation at 0.5 h and its phosphorylation was attenuated. However, 1 mg/ml of shinbuto or ninjinto did not affect LPS-induced ERK phosphorylation (Fig. 5).

### Effects of herbs on PGE_2_ production and molecular expression in the arachidonic acid cascade

**Figure 6:**
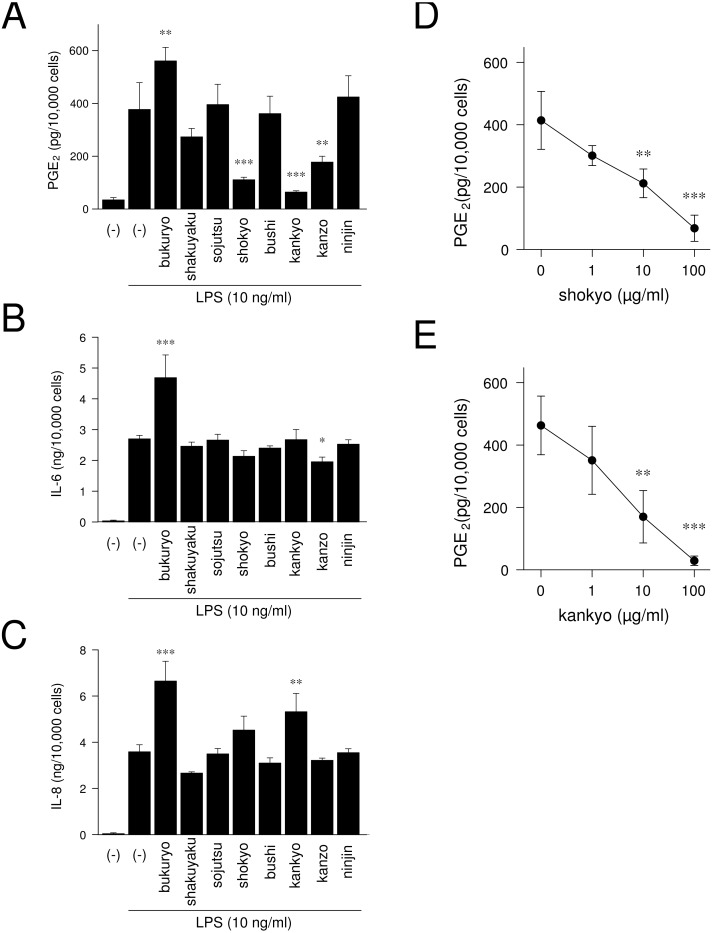
Effects of herbs on LPS-induced PGE_2_, IL-6, and IL-8 production. (A–C) HGFs were treated with combinations of LPS (0 or 10 ng/ml) and each herb (100 µg/ml) for 24 h. Concentrations of PGE_2_ (A), IL-6 (B), and IL-8 (C) were measured by ELISA, adjusted by cell number, and expressed as per 10,000 cells (mean ± S.D., *n* = 3). (D–E) HGFs were treated with combinations of LPS (10 ng/ml) and herbs (0, 1, 10, or 100 µg/ml) for 24 h. Concentrations of PGE_2_ were measured by ELISA, adjusted by cell number, and expressed as per 10,000 cells (mean ± S.D., *n* = 3). ^∗^*P* < 0.05, ^∗∗^*P* < 0.01, ^∗∗∗^*P* < 0.001 (LPS alone vs. LPS plus herb, Dunnett’s test).

We examined whether herbs which comprising shinbuto and ninjinto affected LPS-induced PGE_2_, IL-6 and IL-8 production by HGFs. When HGFs cells were treated with 10 ng/ml of LPS, HGFs cells produced large amounts of PGE_2_. Bukuryo increased LPS-induced PGE_2_ production. Shokyo, kankyo and kanzo strongly and significantly reduced LPS-induced PGE_2_ production (Fig. 6A). Moreover, shokyo and kankyo decreased PGE_2_ production in a concentration-dependent manner (Figs. 6D–6E). Other herbs had little or no effect on PGE_2_ production. Bukuryo increased LPS-induced IL-6 and IL-8 production, and kankyo increased IL-8 production (Figs. 6B–6C). Kanzo reduced IL-6 production (Fig. 6B).

**Figure 7:**
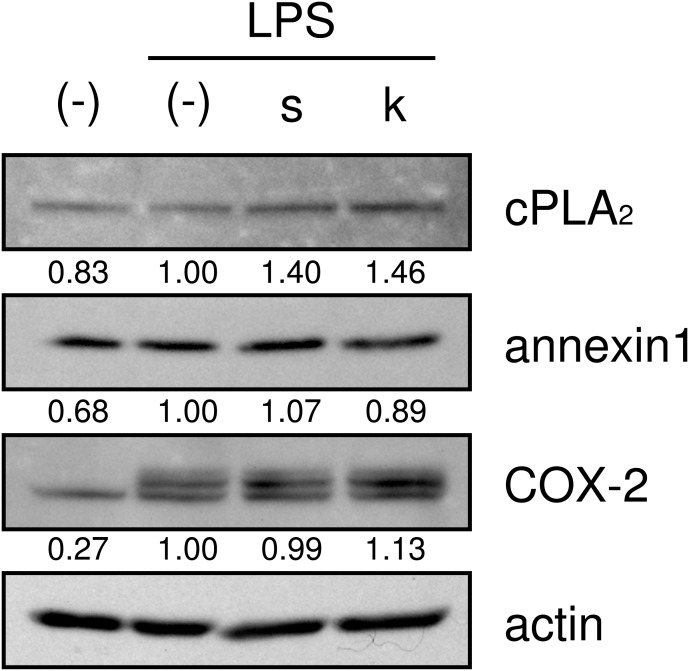
Effects of shokyo and kankyo on cPLA_2_, annexin1, and COX-2 expression. HGFs were treated with a combination of LPS (0 or 10 ng/ml) and herbs (1 mg/ml) for 8 h, and protein levels were examined by Western blotting. The band densities were normalized against LPS alone and actin, and indicated below each band. s, shokyo; k, kankyo.

We then examined whether shokyo and kankyo affected the expression of molecules in the arachidonic acid cascade. Both shokyo and kankyo increased the expression of cPLA_2_ but did not affect annexin1 or COX-2 expression (Fig. 7).

## Discussion

In our previous studies, we reported the importance of HGFs in the study of periodontal disease (Kamemoto et al., 2009; Ara et al., 2010; Nakazono et al., 2010; Ara et al., 2012; Kitamura, Urano & Ara, 2014; Ara & Sogawa, 2016), because HGFs are the most prominent cells in periodontal tissue. Moreover, LPS-treated HGFs produce inflammatory chemical mediators, such as PGE_2_ and inflammatory cytokines such as IL-6 and IL-8 (Sismey-Durrant & Hopps, 1991; Bartold & Haynes, 1991; Tamura et al., 1992). Moreover, HGFs continue to produce PGE_2_ (Ara et al., 2008a), IL-6, and IL-8 (Ara et al., 2009) in the presence of LPS. Therefore, the large amount of chemical mediators and cytokines derived from HGFs may be contained in periodontal tissues. From these findings, we believe that examining the effects of drugs on HGFs is needed in the study of periodontal disease.

In the present study, we examined the effects of kampo medicines on LPS-induced PGE_2_, IL-6, and IL-8 production by HGFs in patients with the deficiency pattern. Shinbuto and ninjinto dose-dependently reduceed LPS-induced PGE_2_ production (Figs. 2B–2C), similar with shosaikoto, hangeshashinto, orento, and kakkonto (Ara et al., 2008b; Nakazono et al., 2010; Ara et al., 2010; Kitamura, Urano & Ara, 2014). However, shinbuto and ninjinto increased LPS-induced IL-6 and IL-8 production (Figs. 2F–2G, 2J–2K). In general, acid non-steroidal anti-inflammatory drugs (NSAIDs) exhibit anti-inflammatory effects by suppressing PGE_2_ production even though they do not affect IL-6 or IL-8 production. Therefore, our results suggest that shinbuto and ninjinto have anti-inflammatory effects in periodontal disease similar with acid NSAIDs.

In the experiments at the herb level, shokyo (*Zingiberis Rhizoma*), kankyo (*Zingiberis Processum Rhizoma*), and kanzo (*Glycyrrhizae Radix*) reduced PGE_2_ production (Fig. 6). Shokyo is contained in shinbuto (Table 1), and kankyo and kanzo are contained in ninjinto (Table 2). Shokyo is the powdered rhizome of ginger (*Zingiber offinale* Roscoe), whereas, kankyo is the steamed and powdered rhizome of ginger. Many reports demonstrated that ginger has anti-inflammatory effects in human (Afzal et al., 2001; Lakhan, Ford & Tepper, 2015), animal (Thomson et al., 2002; Aimbire et al., 2007; El-Abhar, Hammad & Gawad, 2008), and *in vitro* models (Ara & Sogawa, 2016; Podlogar & Verspohl, 2012). Shokyo contains gingerols such as 6-, 8-, and 10-gingerols. With prolonged storage or heat-treatment of ginger, gingerols are converted to shogaols, which are the dehydrated form of the gingerols (Afzal et al., 2001). Therefore, kankyo contains the largest amount of shogaols.

Recently, we found that shokyo suppressed LPS-induced PGE_2_ production by HGFs and that shokyo may suppress PLA_2_ activity (Ara & Sogawa, 2016). In the present study, we examined the effects of kankyo in comparison with shokyo. Shokyo and kankyo increased cPLA_2_ expression but did not alter annexin1 expression (Fig. 7). Moreover, we revealed that shinbuto and ninjinto, which contain shokyo and kankyo respectively, did not alter PGE_2_ production when arachidonic acid was added to bypass the upstream pathway (Fig. 3). These data suggest that shokyo and kankyo did not affect the downstream pathway of arachidonic acid, which includes COX-2 and PGE synthase. In addition, shinbuto and ninjinto did not affect ERK phosphorylation (Fig. 5). From our findings described above, we were unable to explain the mechanism of the reduction in PGE_2_ production. As gingerols in ginger are reported to inhibit both calcium-independent PLA_2_ (iPLA_2_) and cPLA_2_ activities (Nievergelt et al., 2011), shokyo and kankyo are suggested to inhibit PLA_2_ as discussed in the previous study (Ara & Sogawa, 2016). Previously, we reported that cPLA_2_ is the main isoform in HGFs (Ara & Sogawa, 2016) among the subtypes such as cPLA_2_, iPLA_2_, and secretory PLA_2_ (sPLA_2_) (Burke & Dennis, 2009). Therefore, shokyo and kankyo may mainly inhibit cPLA_2_ activity in HGFs. We found that orento decreases LPS-induced PGE_2_ production via the suppression of ERK phosphorylation (Ara et al., 2010). However, orento may also reduce LPS-induced PGE_2_ production by inhibition of cPLA_2_ activity because orento contains kankyo.

We demonstrated that shokyo and kankyo concentration-dependently reduced LPS-induced PGE_2_ production (Fig. 6A), and that the effects of kankyo are slightly stronger than those of shokyo (Figs. 6D–6E). In previous study, 6- and 8-gingerols were found to not inhibit cPLA_2_ activity, but 10-gingerol and 6-, 8-, and 10-shogaols did (Nievergelt et al., 2011). Therefore, the difference in these effects on PGE_2_ production between shokyo and kankyo may be due to the amount of shogaols in these herbs.

We demonstrated that shinbuto and ninjinto slightly increased annexin1 expression (Fig. 4). However, the involvement of annexin1 in the reduction in PGE_2_ production is unlikely. Shokyo and kankyo did not alter annexin1 expression (Fig. 7). All 4 herbs other than shokyo in shinbuto did not reduce PGE_2_ production, but rather, bukuryo increased PGE_2_ production (Fig. 6A). Similarly, kanzo in ninjinto increased annexin1 expression in HGFs, and kanzo also inhibited COX activity (Ara & Sogawa, 2016). The 2 residual herbs other than kankyo and kanzo did not reduce PGE_2_ production (Fig. 6A). Therefore, the increased annexin1 expression did not contribute to decreased PGE_2_ production.

At the herb level, we were unable to clarify which herbs affect cytokine production. Bukuryo in shinbuto increased LPS-induced IL-6 and IL-8 production (Figs. 6B–6C). Therefore, this effect of shinbuto on increased IL-6 production may be due to bukuryo. However, shinbuto did not alter IL-8 production even though it contains bukuryo. Moreover, although ninjinto increased LPS-induced IL-6 production, kanzo reduced IL-6 production, and the other three herbs, kankyo, sojutsu, and ninjin, did not alter IL-6 production. Similarly, although ninjinto did not alter IL-8 production, kankyo increased IL-8 production. Therefore, the effects of herbs on IL-6 and IL-8 production are considered to not be due to a single herb but to the combination of herbs.

Both the expression of COX-2, and the production of IL-6 and IL-8 are widely known to be regulated by NF-*κ*B. Ginger and its components gingerol and shogaol have been reported to suppress NF-*κ*B activation, and to reduce COX-2 expression and the production of IL-6 and IL-8. For example, ginger suppressed NF-*κ*B activation in ovarian cancer cells (Rhode et al., 2007), and 6-gingerol suppressed NF-*κ*B activation in mouse macrophage RAW264.7 cells (Pan et al., 2008), TPA-treated mouse skin *in vivo* (Kim et al., 2005), and in intestinal epithelial cells (Saha et al., 2016). Similarly, 6-shogaol suppressed NF-*κ*B activation in mouse macrophage RAW264.7 cells (Pan et al., 2008) and microglia cells (Ha et al., 2012). 6-Gingerol and 6-shogaol suppressed COX-2 expression in mouse macrophage RAW264.7 cells (Pan et al., 2008) and primary rat astrocytes (Shim et al., 2011). 6-Gingerol reduced the production of IL-1*α*, IL-1*β*, IL-6, and IL-8 in intestinal epithelial cells (Saha et al., 2016). However, shinbuto and ninjinto, which contain shokyo and kankyo, respectively, increased LPS-induced IL-6 and IL-8 production by HGFs (Fig. 2) similar with kakkonto (Kitamura, Urano & Ara, 2014). Moreover, these two kampo medicines, shokyo, and kankyo did not suppress COX-2 expression (Figs. 4A and 7). These findings raised the possibility that shokyo and kankyo, their components, gingerols and shogaols, do not suppress the NF-*κ*B pathway in HGFs. The assumption is able to explain why shokyo and kankyo did not suppress COX-2 expression, which is also regulated by the NF-*κ*B pathway. Furthermore, 6-gingerol and 6-shogaol had no effect on LPS-induced IL-8 production in human bronchial epithelial cells (Podlogar & Verspohl, 2012). Therefore, the effects of gingerols and shogaols may be different among cell types.

## Conclusion

We demonstrated that shinbuto and ninjinto reduced LPS-induced PGE_2_ production by HGFs. Moreover, shokyo and kankyo, which are included in these kampo medicines respectively, concentration-dependently reduced LPS-induced PGE_2_ production. However, shokyo and kankyo did not alter the expression of the molecules in the arachidonic acid cascade, suggesting that shokyo and kankyo inhibit cPLA_2_ activity. Therefore, the kampo medicines that contain shokyo or kankyo may have the ability to reduce PGE_2_ production. We found that the kampo medicines used for patients with the deficiency pattern also have anti-inflammatory effects in those with the excess pattern or medium pattern. We expect kampo medicines to be used for improving inflammatory diseases, such as periodontal disease and stomatitis, in patients with any pattern.

##  Supplemental Information

10.7717/peerj.4120/supp-1 Data S1 Raw data for Figs. 1, 2, 3, and 6

10.7717/peerj.4120/supp-2 Data S2 Raw data for Figs. 4, 5, and 7

10.7717/peerj.4120/supp-3 Data S3 Raw data

## Additional Information and Declarations

### Data Availability

The following information was supplied regarding data availability: The raw data has been provided as Supplemental Files.
