# Supplementary material for: Effects of shinbuto and ninjinto on prostaglandin E2 production in lipopolysaccharide-treated human gingival fibroblasts
Source: PeerJ. 2017 Dec 1;5:e4120. doi: 10.7717/peerj.4120 (PMC5713626; doi:10.7717/peerj.4120)
Supplement: Data S1 [file peerj-05-4120-s001.zip › Fig1/20120725WST_TJ032.pdf]

	drug	LPS	dose	mean	sd
1	TJ032	0	0.000	100.00	2.90
2	TJ032	0	0.500	99.72	4.37
3	TJ032	0	1.000	98.67	3.73
4	TJ032	0	2.000	99.12	6.55
5	TJ032	0	5.000	87.61	7.10
6	TJ032	0	10.000	78.25	3.27
7	TJ032	10	0.000	97.38	9.63
8	TJ032	10	0.500	99.70	1.73
9	TJ032	10	1.000	103.17	1.06
10	TJ032	10	2.000	102.51	5.91
11	TJ032	10	5.000	96.60	8.81
12	TJ032	10	10.000	80.10	4.38

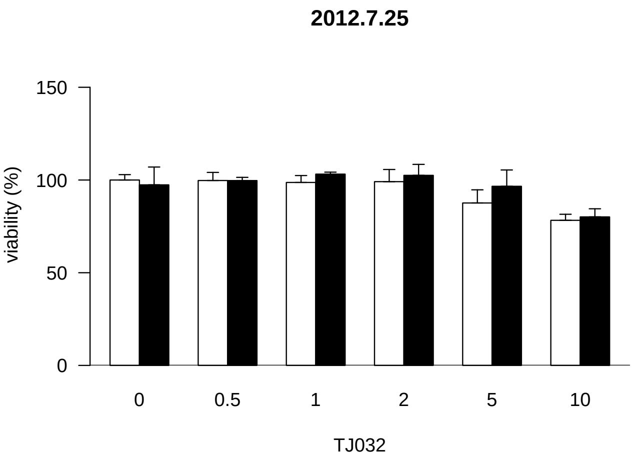

- cells: HGFs (No. 2)
- passages: 8
- cell numbers: 0.5×10^4 cells/well
- LPS: PgLPS (10 ng/ml), treatment: 24h

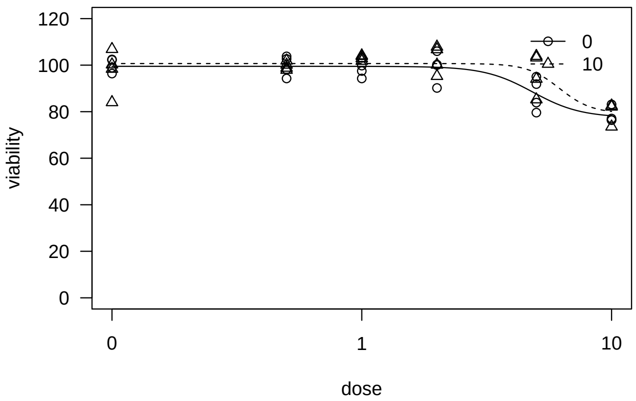

	Estimate	Std. Error	Lower	Upper
0:50	4.83	0.73	3.35	6.31
10:50	6.19	5.04	-3.99	16.37

	drug	OD	mean
1	blank	0.093	0.086
2	blank	0.080	
3	blank	0.086	
4	blank	0.084	
5	blank	0.085	
6	blank	0.086	
7	blank	0.104	
8	blank	0.071	

	drug	LPS	dose	OD	viability
1	TJ032	0	0.000	0.873	96.4
2	TJ032	0	0.000	0.896	98.9
3	TJ032	0	0.000	0.926	102.2
4	TJ032	0	0.000	0.928	102.5
5	TJ032	0	0.500	0.854	94.3
6	TJ032	0	0.500	0.940	103.8
7	TJ032	0	0.500	0.930	102.7
8	TJ032	0	0.500	0.889	98.2
9	TJ032	0	1.000	0.934	103.1
10	TJ032	0	1.000	0.904	99.8
11	TJ032	0	1.000	0.854	94.3
12	TJ032	0	1.000	0.883	97.5
13	TJ032	0	2.000	0.817	90.2
14	TJ032	0	2.000	0.960	106.0
15	TJ032	0	2.000	0.906	100.0
16	TJ032	0	2.000	0.908	100.2
17	TJ032	0	5.000	0.721	79.6
18	TJ032	0	5.000	0.760	83.9
19	TJ032	0	5.000	0.832	91.9
20	TJ032	0	5.000	0.861	95.1
21	TJ032	0	10.000	0.692	76.4
22	TJ032	0	10.000	0.692	76.4
23	TJ032	0	10.000	0.753	83.1
24	TJ032	0	10.000	0.698	77.1
25	TJ032	10	0.000	0.968	106.9
26	TJ032	10	0.000	0.761	84.0
27	TJ032	10	0.000	0.908	100.2
28	TJ032	10	0.000	0.891	98.4
29	TJ032	10	0.500	0.923	101.9
30	TJ032	10	0.500	0.907	100.1
31	TJ032	10	0.500	0.895	98.8
32	TJ032	10	0.500	0.887	97.9
33	TJ032	10	1.000	0.932	102.9
34	TJ032	10	1.000	0.943	104.1
35	TJ032	10	1.000	0.941	103.9
36	TJ032	10	1.000	0.922	101.8
37	TJ032	10	2.000	0.967	106.8
38	TJ032	10	2.000	0.907	100.1
39	TJ032	10	2.000	0.977	107.9
40	TJ032	10	2.000	0.863	95.3
41	TJ032	10	5.000	0.935	103.2
42	TJ032	10	5.000	0.852	94.1
43	TJ032	10	5.000	0.941	103.9
44	TJ032	10	5.000	0.772	85.2
45	TJ032	10	10.000	0.744	82.1
46	TJ032	10	10.000	0.744	82.1
47	TJ032	10	10.000	0.748	82.6
48	TJ032	10	10.000	0.666	73.5
