# Supplementary material for: Effects of shinbuto and ninjinto on prostaglandin E2 production in lipopolysaccharide-treated human gingival fibroblasts
Source: PeerJ. 2017 Dec 1;5:e4120. doi: 10.7717/peerj.4120 (PMC5713626; doi:10.7717/peerj.4120)
Supplement: Data S1 [file peerj-05-4120-s001.zip › Fig1/20120608WST_TJ029.pdf]

```
'drc' has been loaded.

Please cite R and 'drc' if used for a publication,
for references type 'citation()' and 'citation('drc')'.
```

	drug	LPS	dose	mean	sd
1	TJ029	0	0.000	100.00	4.23
2	TJ029	0	0.500	85.52	2.93
3	TJ029	0	1.000	88.05	4.19
4	TJ029	0	2.000	90.75	4.42
5	TJ029	0	5.000	93.05	2.75
6	TJ029	0	10.000	84.37	7.39
7	TJ029	10	0.000	98.05	4.38
8	TJ029	10	0.500	82.19	4.17
9	TJ029	10	1.000	91.09	0.47
10	TJ029	10	2.000	86.44	5.00
11	TJ029	10	5.000	91.50	9.95
12	TJ029	10	10.000	91.73	5.80

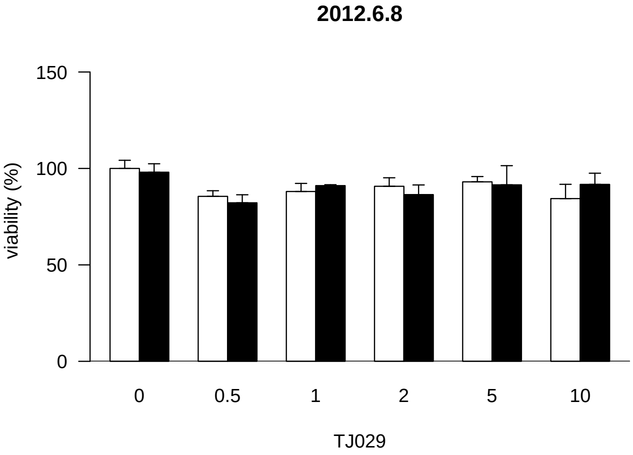

- cells: HGFs (No. 2)
- passages: 14
- cell numbers: 0.5×10^4 cells/well
- LPS: PgLPS (10 ng/ml), treatment: 24h

	drug	OD	mean
1	blank	0.099	0.098
2	blank	0.094	
3	blank	0.098	
4	blank	0.098	
5	blank	0.098	
6	blank	0.101	
7	blank	0.105	
8	blank	0.090	

	drug	LPS	dose	OD	viability
1	TJ029	0	0.000	0.443	101.8
2	TJ029	0	0.000	0.449	103.2
3	TJ029	0	0.000	0.408	93.8
4	TJ029	0	0.000	0.440	101.1
5	TJ029	0	0.500	0.375	86.2
6	TJ029	0	0.500	0.388	89.2
7	TJ029	0	0.500	0.358	82.3
8	TJ029	0	0.500	0.367	84.4
9	TJ029	0	1.000	0.374	86.0
10	TJ029	0	1.000	0.364	83.7
11	TJ029	0	1.000	0.388	89.2
12	TJ029	0	1.000	0.406	93.3
13	TJ029	0	2.000	0.393	90.3
14	TJ029	0	2.000	0.372	85.5
15	TJ029	0	2.000	0.419	96.3
16	TJ029	0	2.000	0.395	90.8
17	TJ029	0	5.000	0.390	89.7
18	TJ029	0	5.000	0.400	92.0
19	TJ029	0	5.000	0.414	95.2
20	TJ029	0	5.000	0.415	95.4
21	TJ029	0	10.000	0.407	93.6
22	TJ029	0	10.000	0.335	77.0
23	TJ029	0	10.000	0.378	86.9
24	TJ029	0	10.000	0.348	80.0
25	TJ029	10	0.000	0.406	93.3
26	TJ029	10	0.000	0.452	103.9
27	TJ029	10	0.000	0.422	97.0
28	TJ029	10	0.000	0.426	97.9
29	TJ029	10	0.500	0.375	86.2
30	TJ029	10	0.500	0.371	85.3
31	TJ029	10	0.500	0.339	77.9
32	TJ029	10	0.500	0.345	79.3
33	TJ029	10	1.000	0.398	91.5
34	TJ029	10	1.000	0.394	90.6
35	TJ029	10	1.000	0.395	90.8
36	TJ029	10	1.000	0.398	91.5
37	TJ029	10	2.000	0.387	89.0
38	TJ029	10	2.000	0.400	92.0
39	TJ029	10	2.000	0.351	80.7
40	TJ029	10	2.000	0.366	84.1
41	TJ029	10	5.000	0.408	93.8
42	TJ029	10	5.000	0.443	101.8
43	TJ029	10	5.000	0.402	92.4
44	TJ029	10	5.000	0.339	77.9
45	TJ029	10	10.000	0.402	92.4
46	TJ029	10	10.000	0.372	85.5
47	TJ029	10	10.000	0.390	89.7
48	TJ029	10	10.000	0.432	99.3
