# Supplementary material for: Effects of shinbuto and ninjinto on prostaglandin E2 production in lipopolysaccharide-treated human gingival fibroblasts
Source: PeerJ. 2017 Dec 1;5:e4120. doi: 10.7717/peerj.4120 (PMC5713626; doi:10.7717/peerj.4120)
Supplement: Data S1 [file peerj-05-4120-s001.zip › Fig1/20120725WST_TJ041.pdf]

	drug	LPS	dose	mean	sd
1	TJ041	0	0.000	100.00	3.83
2	TJ041	0	0.500	98.25	1.83
3	TJ041	0	1.000	97.18	1.62
4	TJ041	0	2.000	97.87	2.26
5	TJ041	0	5.000	85.06	1.53
6	TJ041	0	10.000	69.69	4.82
7	TJ041	10	0.000	101.12	3.79
8	TJ041	10	0.500	98.93	2.40
9	TJ041	10	1.000	99.64	2.18
10	TJ041	10	2.000	99.21	2.65
11	TJ041	10	5.000	91.81	5.45
12	TJ041	10	10.000	71.87	3.92

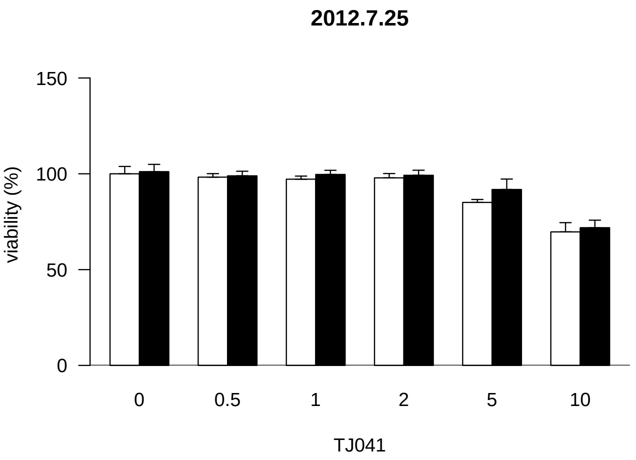

- cells: HGFs (No. 2)
- passages: 8
- cell numbers: 0.5×10^4 cells/well
- LPS: PgLPS (10 ng/ml), treatment: 24h

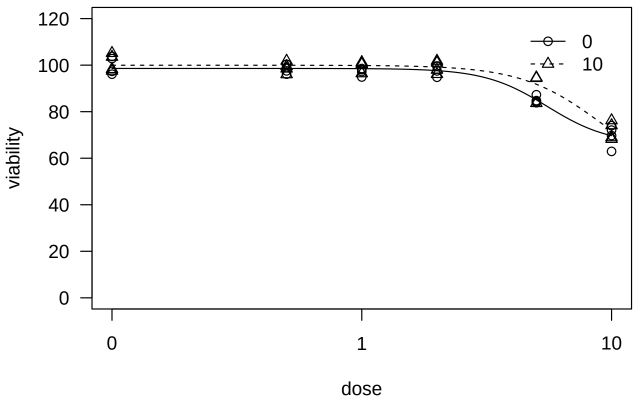

	Estimate	Std. Error	Lower	Upper
0:50	5.52	1.34	2.81	8.23
10:50	9.12	12.65	-16.44	34.68

	drug	OD	mean
1	blank	0.094	0.086
2	blank	0.081	
3	blank	0.086	
4	blank	0.087	
5	blank	0.088	
6	blank	0.086	
7	blank	0.094	
8	blank	0.070	

	drug	LPS	dose	OD	viability
1	TJ041	0	0.000	0.959	97.3
2	TJ041	0	0.000	1.013	102.8
3	TJ041	0	0.000	0.947	96.1
4	TJ041	0	0.000	1.022	103.7
5	TJ041	0	0.500	0.989	100.4
6	TJ041	0	0.500	0.961	97.5
7	TJ041	0	0.500	0.947	96.1
8	TJ041	0	0.500	0.975	99.0
9	TJ041	0	1.000	0.958	97.2
10	TJ041	0	1.000	0.935	94.9
11	TJ041	0	1.000	0.971	98.6
12	TJ041	0	1.000	0.966	98.0
13	TJ041	0	2.000	0.980	99.5
14	TJ041	0	2.000	0.961	97.5
15	TJ041	0	2.000	0.982	99.7
16	TJ041	0	2.000	0.934	94.8
17	TJ041	0	5.000	0.835	84.8
18	TJ041	0	5.000	0.826	83.8
19	TJ041	0	5.000	0.831	84.3
20	TJ041	0	5.000	0.860	87.3
21	TJ041	0	10.000	0.710	72.1
22	TJ041	0	10.000	0.687	69.7
23	TJ041	0	10.000	0.729	74.0
24	TJ041	0	10.000	0.620	62.9
25	TJ041	10	0.000	0.961	97.5
26	TJ041	10	0.000	0.968	98.2
27	TJ041	10	0.000	1.020	103.5
28	TJ041	10	0.000	1.036	105.1
29	TJ041	10	0.500	0.970	98.5
30	TJ041	10	0.500	0.946	96.0
31	TJ041	10	0.500	0.980	99.5
32	TJ041	10	0.500	1.003	101.8
33	TJ041	10	1.000	0.950	96.4
34	TJ041	10	1.000	0.991	100.6
35	TJ041	10	1.000	0.997	101.2
36	TJ041	10	1.000	0.989	100.4
37	TJ041	10	2.000	0.947	96.1
38	TJ041	10	2.000	0.965	97.9
39	TJ041	10	2.000	1.003	101.8
40	TJ041	10	2.000	0.995	101.0
41	TJ041	10	5.000	0.930	94.4
42	TJ041	10	5.000	0.824	83.6
43	TJ041	10	5.000	0.932	94.6
44	TJ041	10	5.000	0.932	94.6
45	TJ041	10	10.000	0.679	68.9
46	TJ041	10	10.000	0.730	74.1
47	TJ041	10	10.000	0.751	76.2
48	TJ041	10	10.000	0.672	68.2
