# Supplementary material for: Effects of shinbuto and ninjinto on prostaglandin E2 production in lipopolysaccharide-treated human gingival fibroblasts
Source: PeerJ. 2017 Dec 1;5:e4120. doi: 10.7717/peerj.4120 (PMC5713626; doi:10.7717/peerj.4120)
Supplement: Data S1 [file peerj-05-4120-s001.zip › Fig1/20120725WST_TJ030.pdf]

	drug	LPS	dose	mean	sd
1	TJ030	0	0.000	100.00	6.19
2	TJ030	0	0.500	97.72	8.39
3	TJ030	0	1.000	98.92	9.06
4	TJ030	0	2.000	96.18	3.72
5	TJ030	0	5.000	81.87	4.05
6	TJ030	0	10.000	60.04	1.75
7	TJ030	10	0.000	100.88	2.81
8	TJ030	10	0.500	105.05	6.54
9	TJ030	10	1.000	107.04	3.88
10	TJ030	10	2.000	99.21	8.20
11	TJ030	10	5.000	91.30	7.38
12	TJ030	10	10.000	68.09	3.12

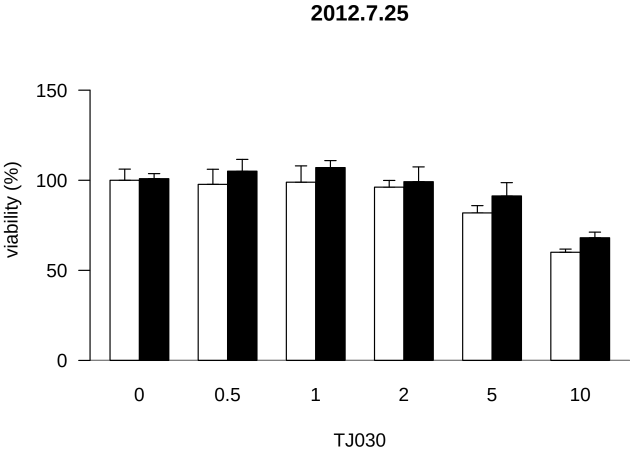

- cells: HGFs (No. 2)
- passages: 8
- cell numbers: 0.5×10^4 cells/well
- LPS: PgLPS (10 ng/ml), treatment: 24h

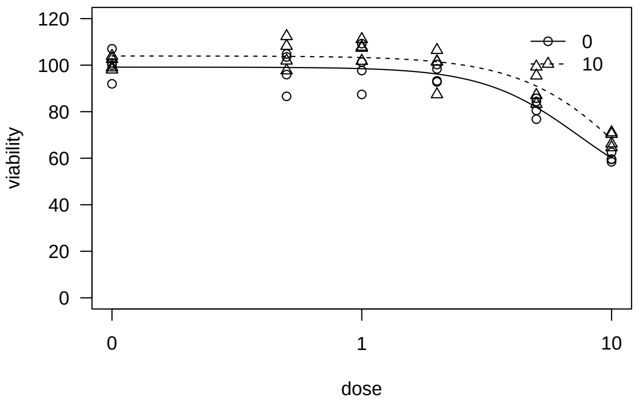

	Estimate	Std. Error	Lower	Upper
0:50	7.40	6.25	-5.23	20.04
10:50	13.13	25.90	-39.21	65.47

	drug	OD	mean
1	blank	0.083	0.082
2	blank	0.077	
3	blank	0.082	
4	blank	0.081	
5	blank	0.083	
6	blank	0.083	
7	blank	0.101	
8	blank	0.069	

	drug	LPS	dose	OD	viability
1	TJ030	0	0.000	0.788	92.0
2	TJ030	0	0.000	0.857	100.0
3	TJ030	0	0.000	0.865	101.0
4	TJ030	0	0.000	0.917	107.0
5	TJ030	0	0.500	0.742	86.6
6	TJ030	0	0.500	0.887	103.5
7	TJ030	0	0.500	0.822	95.9
8	TJ030	0	0.500	0.898	104.8
9	TJ030	0	1.000	0.749	87.4
10	TJ030	0	1.000	0.868	101.3
11	TJ030	0	1.000	0.837	97.7
12	TJ030	0	1.000	0.936	109.3
13	TJ030	0	2.000	0.795	92.8
14	TJ030	0	2.000	0.843	98.4
15	TJ030	0	2.000	0.859	100.3
16	TJ030	0	2.000	0.799	93.3
17	TJ030	0	5.000	0.658	76.8
18	TJ030	0	5.000	0.690	80.5
19	TJ030	0	5.000	0.723	84.4
20	TJ030	0	5.000	0.735	85.8
21	TJ030	0	10.000	0.536	62.5
22	TJ030	0	10.000	0.511	59.6
23	TJ030	0	10.000	0.501	58.5
24	TJ030	0	10.000	0.510	59.5
25	TJ030	10	0.000	0.879	102.6
26	TJ030	10	0.000	0.890	103.9
27	TJ030	10	0.000	0.848	99.0
28	TJ030	10	0.000	0.840	98.0
29	TJ030	10	0.500	0.873	101.9
30	TJ030	10	0.500	0.963	112.4
31	TJ030	10	0.500	0.927	108.2
32	TJ030	10	0.500	0.837	97.7
33	TJ030	10	1.000	0.924	107.9
34	TJ030	10	1.000	0.920	107.4
35	TJ030	10	1.000	0.952	111.1
36	TJ030	10	1.000	0.872	101.8
37	TJ030	10	2.000	0.870	101.5
38	TJ030	10	2.000	0.869	101.4
39	TJ030	10	2.000	0.912	106.5
40	TJ030	10	2.000	0.749	87.4
41	TJ030	10	5.000	0.818	95.5
42	TJ030	10	5.000	0.746	87.1
43	TJ030	10	5.000	0.851	99.3
44	TJ030	10	5.000	0.714	83.3
45	TJ030	10	10.000	0.603	70.4
46	TJ030	10	10.000	0.554	64.6
47	TJ030	10	10.000	0.609	71.1
48	TJ030	10	10.000	0.568	66.3
