# Supplementary material for: Effects of shinbuto and ninjinto on prostaglandin E2 production in lipopolysaccharide-treated human gingival fibroblasts
Source: PeerJ. 2017 Dec 1;5:e4120. doi: 10.7717/peerj.4120 (PMC5713626; doi:10.7717/peerj.4120)
Supplement: Data S1 [file peerj-05-4120-s001.zip › Fig2/006_PgLPS_TJ030_PGE2-1.pdf]

- Exp. 6
- Condition
  - drug1: PgLPS (pg/ml)
  - drug2: TJ030 (mg/ml)
  - experimental No. 1
  - treatment: 24h
- Measurement
  - PGE2
  - Date: 2012.7.6
- Cells
  - cells: HGFs (No. 1), passages: 15
  - cell numbers: 1×10^4 cells/well = 5×10^4 cells/ml

	conc.	OD
1	7.8	0.757
2	15.6	0.673
3	31.2	0.542
4	62.5	0.465
5	125.0	0.316
6	250.0	0.239
7	500.0	0.191
8	1000.0	0.170

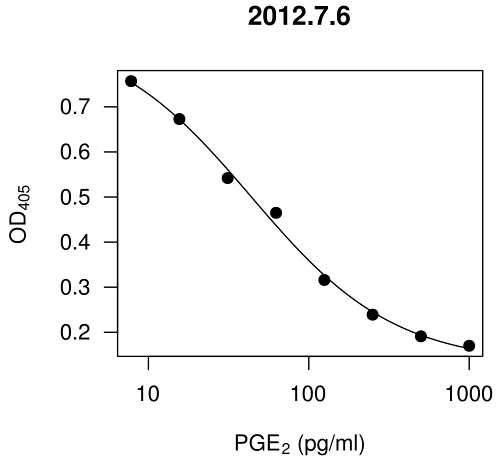

	drug1	drug2	mean	SD
1	0	0.000	0.036	0.010
2	0	0.010	0.037	0.011
3	0	0.100	0.025	0.002
4	0	1.000	0.025	0.005
5	10	0.000	0.467	0.095
6	10	0.010	0.463	0.020
7	10	0.100	0.378	0.098
8	10	1.000	0.125	0.032

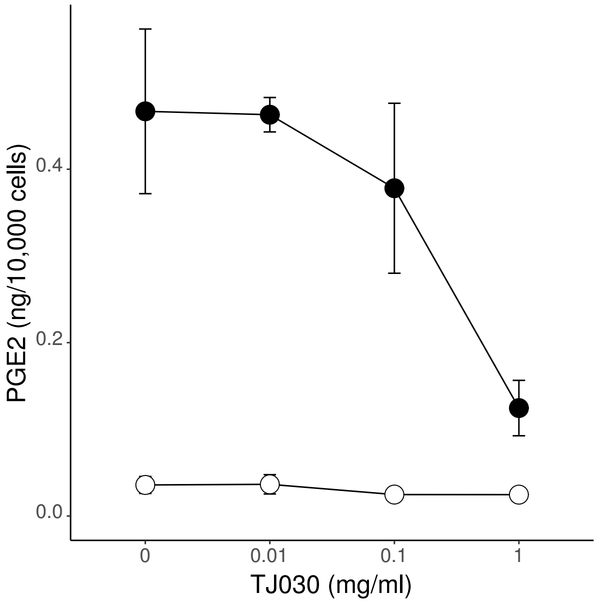

	drug1	drug2	viability	dilution	OD	conc. (pg/ml)	net (ng/ml)	(ng/10,000 cells)
1	0	0.000	103.12	5	0.574	28.94	0.145	0.028
2	0	0.000	96.10	5	0.560	31.36	0.157	0.033
3	0	0.000	100.78	5	0.487	47.17	0.236	0.047
4	0	0.010	103.41	5	0.601	24.69	0.123	0.024
5	0	0.010	96.10	5	0.504	42.92	0.215	0.045
6	0	0.010	98.59	5	0.514	40.60	0.203	0.041
7	0	0.100	104.29	5	0.611	23.24	0.116	0.022
8	0	0.100	96.54	5	0.605	24.10	0.121	0.025
9	0	0.100	98.73	5	0.588	26.67	0.133	0.027
10	0	1.000	102.39	5	0.613	22.96	0.115	0.022
11	0	1.000	96.98	5	0.568	29.95	0.150	0.031
12	0	1.000	101.07	5	0.628	20.92	0.105	0.021
13	10	0.000	100.93	5	0.205	414.10	2.070	0.410
14	10	0.000	101.66	5	0.204	420.51	2.103	0.414
15	10	0.000	99.90	5	0.186	575.81	2.879	0.576
16	10	0.010	98.59	5	0.196	478.90	2.395	0.486
17	10	0.010	99.03	5	0.200	448.01	2.240	0.452
18	10	0.010	99.46	5	0.200	448.01	2.240	0.450
19	10	0.100	98.00	5	0.239	267.03	1.335	0.272
20	10	0.100	103.12	5	0.206	407.86	2.039	0.396
21	10	0.100	99.32	5	0.198	462.99	2.315	0.466
22	10	1.000	99.03	5	0.370	93.33	0.467	0.094
23	10	1.000	101.95	5	0.293	160.99	0.805	0.158
24	10	1.000	100.34	5	0.330	121.85	0.609	0.121
