# Supplementary material for: Effects of shinbuto and ninjinto on prostaglandin E2 production in lipopolysaccharide-treated human gingival fibroblasts
Source: PeerJ. 2017 Dec 1;5:e4120. doi: 10.7717/peerj.4120 (PMC5713626; doi:10.7717/peerj.4120)
Supplement: Data S1 [file peerj-05-4120-s001.zip › Fig2/006_PgLPS_TJ032_WST-1.pdf]

- Exp. 6
- Condition
  - drug1: PgLPS (pg/ml)
  - drug2: TJ032 (mg/ml)
  - experimental No. 1
  - treatment: 24h
- Measurement
  - WST-8
  - Date: 2012.7.5
- Cells
  - cells: HGFs (No. 1), passages: 15
  - cell numbers: 1×10^4 cells/well

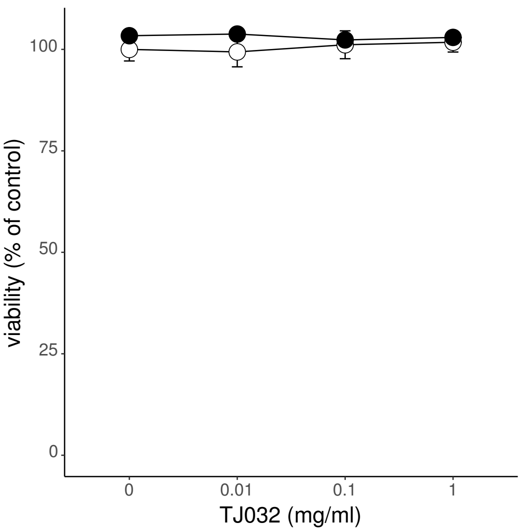

	drug1	drug2	mean	SD
1	0	0.000	100.0	2.9
2	0	0.010	99.4	3.7
3	0	0.100	101.1	3.4
4	0	1.000	101.8	2.4
5	10	0.000	103.4	0.8
6	10	0.010	103.8	1.2
7	10	0.100	102.3	0.9
8	10	1.000	103.0	1.3

	OD	mean
1	0.056	0.056
2	0.047	
3	0.053	
4	0.053	
5	0.054	
6	0.071	
7	0.058	
8	0.054	

	drug1	drug2	OD	OD-blank	viability
1	0	0.000	0.713	0.657	102.2
2	0	0.000	0.678	0.622	96.8
3	0	0.000	0.705	0.649	101.0
4	0	0.010	0.713	0.657	102.2
5	0	0.010	0.668	0.612	95.2
6	0	0.010	0.703	0.647	100.7
7	0	0.100	0.727	0.671	104.4
8	0	0.100	0.683	0.627	97.6
9	0	0.100	0.708	0.652	101.5
10	0	1.000	0.725	0.669	104.1
11	0	1.000	0.694	0.638	99.3
12	0	1.000	0.711	0.655	101.9
13	10	0.000	0.719	0.663	103.2
14	10	0.000	0.726	0.670	104.3
15	10	0.000	0.716	0.660	102.7
16	10	0.010	0.732	0.676	105.2
17	10	0.010	0.718	0.662	103.0
18	10	0.010	0.719	0.663	103.2
19	10	0.100	0.710	0.654	101.8
20	10	0.100	0.720	0.664	103.3
21	10	0.100	0.711	0.655	101.9
22	10	1.000	0.709	0.653	101.6
23	10	1.000	0.726	0.670	104.3
24	10	1.000	0.718	0.662	103.0
