# Supplementary material for: Effects of shinbuto and ninjinto on prostaglandin E2 production in lipopolysaccharide-treated human gingival fibroblasts
Source: PeerJ. 2017 Dec 1;5:e4120. doi: 10.7717/peerj.4120 (PMC5713626; doi:10.7717/peerj.4120)
Supplement: Data S1 [file peerj-05-4120-s001.zip › Fig2/006_PgLPS_TJ032_IL-8-1.pdf]

- Exp. 6
- Condition
  - drug1: PgLPS (pg/ml)
  - drug2: TJ030 (mg/ml)
  - experimental No. 1
  - treatment: 24h
- Measurement
  - IL-8
  - Date: 2012.11.5
- Cells
  - cells: HGFs (No. 1), passages: 15
  - cell numbers: 1×10^4 cells/well = 5×10^4 cells/ml

	conc.	OD	OD-blank
1	0.0	0.073	0.000
2	15.6	0.122	0.049
3	31.2	0.154	0.081
4	62.5	0.227	0.154
5	125.0	0.347	0.274
6	250.0	0.571	0.498
7	500.0	0.875	0.802
8	1000.0	1.179	1.106

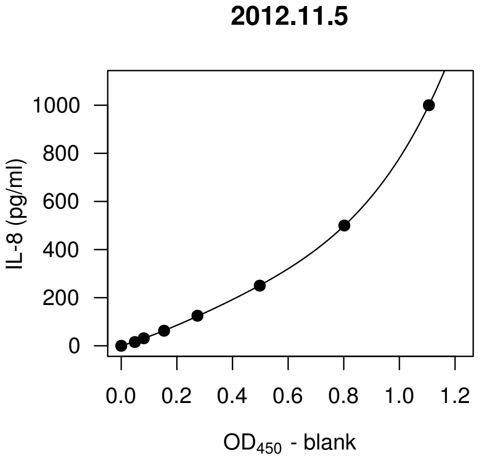

	drug1	drug2	mean	SD
1	0	0.000	0.003	0.004
2	0	0.010	0.025	0.011
3	0	0.100	0.034	0.008
4	0	1.000	0.064	0.004
5	10	0.000	3.418	0.329
6	10	0.010	3.341	0.245
7	10	0.100	3.120	0.312
8	10	1.000	3.416	0.072

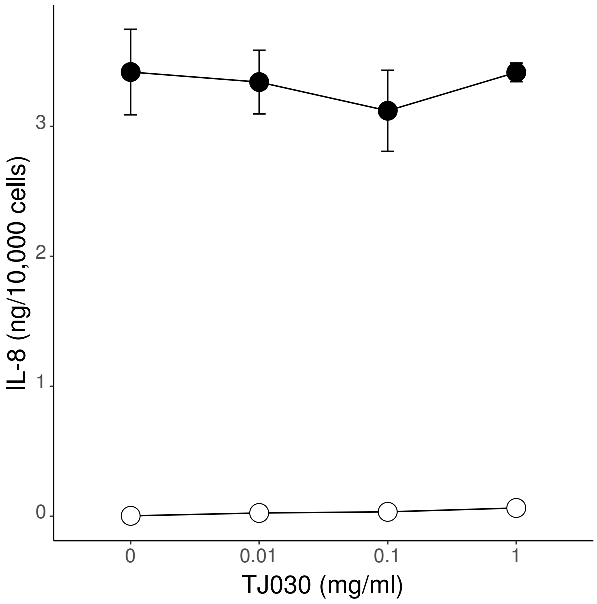

	drug1	drug2	viability	dilution	OD	conc. (pg/ml)	net (ng/ml)	(ng/10,000 cells)
1	0	0.000	102.23	50	0.074	0.34	0.017	0.003
2	0	0.000	96.79	50	0.075	0.68	0.034	0.007
3	0	0.000	100.99	50	0.073	0.00	0.000	0.000
4	0	0.010	102.23	50	0.077	1.36	0.068	0.013
5	0	0.010	95.23	50	0.081	2.74	0.137	0.029
6	0	0.010	100.67	50	0.083	3.44	0.172	0.034
7	0	0.100	104.41	50	0.081	2.74	0.137	0.026
8	0	0.100	97.56	50	0.085	4.14	0.207	0.042
9	0	0.100	101.45	50	0.083	3.44	0.172	0.034
10	0	1.000	104.10	50	0.093	6.98	0.349	0.067
11	0	1.000	99.27	50	0.090	5.91	0.295	0.060
12	0	1.000	101.92	50	0.092	6.62	0.331	0.065
13	10	0.000	103.16	50	0.729	362.98	18.149	3.519
14	10	0.000	104.25	50	0.670	318.02	15.901	3.051
15	10	0.000	102.70	50	0.748	378.51	18.925	3.686
16	10	0.010	105.18	50	0.675	321.66	16.083	3.058
17	10	0.010	103.01	50	0.723	358.19	17.910	3.477
18	10	0.010	103.16	50	0.725	359.78	17.989	3.488
19	10	0.100	101.76	50	0.670	318.02	15.901	3.125
20	10	0.100	103.32	50	0.630	289.90	14.495	2.806
21	10	0.100	101.92	50	0.712	349.55	17.477	3.430
22	10	1.000	101.61	50	0.698	338.79	16.939	3.334
23	10	1.000	104.25	50	0.724	358.99	17.949	3.444
24	10	1.000	103.01	50	0.722	357.40	17.870	3.470
