# Supplementary material for: Effects of shinbuto and ninjinto on prostaglandin E2 production in lipopolysaccharide-treated human gingival fibroblasts
Source: PeerJ. 2017 Dec 1;5:e4120. doi: 10.7717/peerj.4120 (PMC5713626; doi:10.7717/peerj.4120)
Supplement: Data S1 [file peerj-05-4120-s001.zip › Fig2/006_PgLPS_TJ029_IL-8-1.pdf]

- Exp. 6
- Condition
  - drug1: PgLPS (pg/ml)
  - drug2: TJ029 (mg/ml)
  - experimental No. 1
  - treatment: 24h
- Measurement
  - IL-8
  - Date: 2012.11.5
- Cells
  - cells: HGFs (No. 1), passages: 15
  - cell numbers: $1 \times 10^4$ cells/well = $5 \times 10^4$ cells/ml

| | conc. | OD | OD-blank |
|---|---|---|---|
| 1 | 0.0 | 0.053 | 0.000 |
| 2 | 15.6 | 0.092 | 0.039 |
| 3 | 31.2 | 0.120 | 0.067 |
| 4 | 62.5 | 0.188 | 0.135 |
| 5 | 125.0 | 0.295 | 0.242 |
| 6 | 250.0 | 0.493 | 0.440 |
| 7 | 500.0 | 0.793 | 0.740 |
| 8 | 1000.0 | 1.079 | 1.026 |

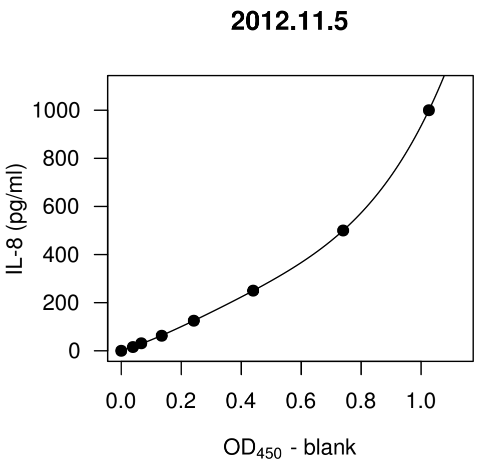


| | drug1 | drug2 | mean | SD |
|---|---|---|---|---|
| 1 | 0 | 0.000 | 0.009 | 0.008 |
| 2 | 0 | 0.010 | 0.004 | 0.004 |
| 3 | 0 | 0.100 | 0.002 | 0.004 |
| 4 | 0 | 1.000 | 0.005 | 0.009 |
| 5 | 10 | 0.000 | 3.942 | 0.421 |
| 6 | 10 | 0.010 | 4.014 | 0.147 |
| 7 | 10 | 0.100 | 4.409 | 0.422 |
| 8 | 10 | 1.000 | 4.547 | 0.237 |

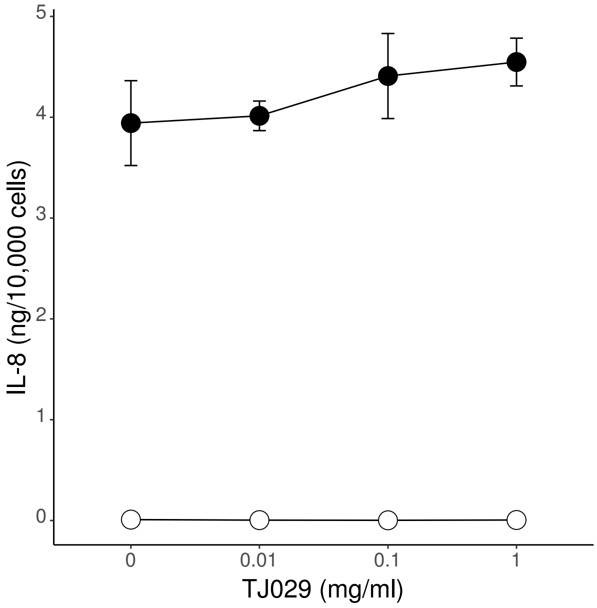

| | drug1 | drug2 | viability | dilution | OD | conc. (pg/ml) | net (ng/ml) | (ng/10,000 cells) |
|---|---|---|---|---|---|---|---|---|
| 1 | 0 | 0.000 | 103.89 | 50 | 0.057 | 1.54 | 0.077 | 0.015 |
| 2 | 0 | 0.000 | 95.67 | 50 | 0.056 | 1.15 | 0.058 | 0.012 |
| 3 | 0 | 0.000 | 100.43 | 50 | 0.053 | 0.00 | 0.000 | 0.000 |
| 4 | 0 | 0.010 | 104.18 | 50 | 0.053 | 0.00 | 0.000 | 0.000 |
| 5 | 0 | 0.010 | 96.68 | 50 | 0.054 | 0.38 | 0.019 | 0.004 |
| 6 | 0 | 0.010 | 99.28 | 50 | 0.055 | 0.77 | 0.038 | 0.008 |
| 7 | 0 | 0.100 | 104.04 | 50 | 0.055 | 0.77 | 0.038 | 0.007 |
| 8 | 0 | 0.100 | 95.67 | 50 | 0.053 | 0.00 | 0.000 | 0.000 |
| 9 | 0 | 0.100 | 98.85 | 50 | 0.053 | 0.00 | 0.000 | 0.000 |
| 10 | 0 | 1.000 | 101.15 | 50 | 0.053 | 0.00 | 0.000 | 0.000 |
| 11 | 0 | 1.000 | 96.54 | 50 | 0.053 | 0.00 | 0.000 | 0.000 |
| 12 | 0 | 1.000 | 100.43 | 50 | 0.057 | 1.54 | 0.077 | 0.015 |
| 13 | 10 | 0.000 | 100.14 | 50 | 0.696 | 403.17 | 20.158 | 4.026 |
| 14 | 10 | 0.000 | 101.44 | 50 | 0.637 | 353.55 | 17.678 | 3.485 |
| 15 | 10 | 0.000 | 99.42 | 50 | 0.724 | 428.90 | 21.445 | 4.314 |
| 16 | 10 | 0.010 | 98.13 | 50 | 0.696 | 403.17 | 20.158 | 4.108 |
| 17 | 10 | 0.010 | 99.71 | 50 | 0.701 | 407.65 | 20.382 | 4.088 |
| 18 | 10 | 0.010 | 98.99 | 50 | 0.670 | 380.60 | 19.030 | 3.845 |
| 19 | 10 | 0.100 | 100.29 | 50 | 0.694 | 401.39 | 20.069 | 4.002 |
| 20 | 10 | 0.100 | 101.01 | 50 | 0.738 | 442.39 | 22.119 | 4.380 |
| 21 | 10 | 0.100 | 98.27 | 50 | 0.771 | 476.04 | 23.802 | 4.844 |
| 22 | 10 | 1.000 | 98.99 | 50 | 0.772 | 477.11 | 23.855 | 4.820 |
| 23 | 10 | 1.000 | 102.16 | 50 | 0.744 | 448.31 | 22.415 | 4.388 |
| 24 | 10 | 1.000 | 100.43 | 50 | 0.741 | 445.34 | 22.267 | 4.434 |
