# Supplementary material for: Effects of shinbuto and ninjinto on prostaglandin E2 production in lipopolysaccharide-treated human gingival fibroblasts
Source: PeerJ. 2017 Dec 1;5:e4120. doi: 10.7717/peerj.4120 (PMC5713626; doi:10.7717/peerj.4120)
Supplement: Data S1 [file peerj-05-4120-s001.zip › Fig2/006_PgLPS_TJ029_WST-1.pdf]

- Exp. 6
- Condition
  - drug1: PgLPS (pg/ml)
  - drug2: TJ029 (mg/ml)
  - experimental No. 1
  - treatment: 24h
- Measurement
  - WST-8
  - Date: 2012.7.5
- Cells
  - cells: HGFs (No. 1), passages: 15
  - cell numbers: 1×10^4 cells/well

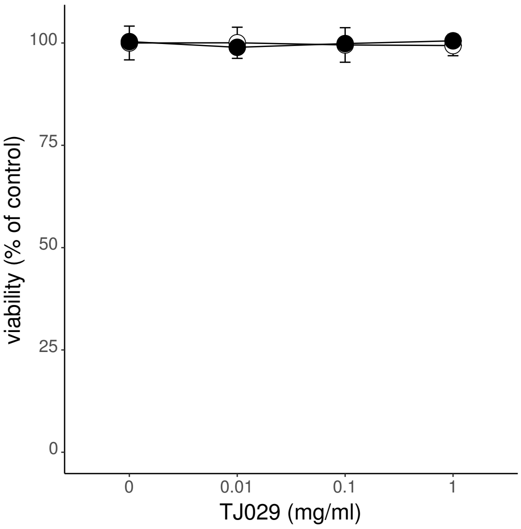

	drug1	drug2	mean	SD
1	0	0.000	100.0	4.1
2	0	0.010	100.0	3.8
3	0	0.100	99.5	4.2
4	0	1.000	99.4	2.5
5	10	0.000	100.3	1.0
6	10	0.010	98.9	0.8
7	10	0.100	99.9	1.4
8	10	1.000	100.5	1.6

	drug1	drug2	OD	OD-blank	viability
1	0	0.000	0.773	0.721	103.9
2	0	0.000	0.716	0.663	95.7
3	0	0.000	0.749	0.697	100.4
4	0	0.010	0.775	0.723	104.2
5	0	0.010	0.723	0.670	96.7
6	0	0.010	0.741	0.689	99.3
7	0	0.100	0.774	0.722	104.0
8	0	0.100	0.716	0.663	95.7
9	0	0.100	0.738	0.685	98.8
10	0	1.000	0.754	0.702	101.2
11	0	1.000	0.722	0.669	96.5
12	0	1.000	0.749	0.697	100.4
13	10	0.000	0.747	0.695	100.1
14	10	0.000	0.756	0.704	101.4
15	10	0.000	0.742	0.690	99.4
16	10	0.010	0.733	0.680	98.1
17	10	0.010	0.744	0.692	99.7
18	10	0.010	0.739	0.686	99.0
19	10	0.100	0.748	0.696	100.3
20	10	0.100	0.753	0.701	101.0
21	10	0.100	0.734	0.681	98.3
22	10	1.000	0.739	0.686	99.0
23	10	1.000	0.761	0.709	102.2
24	10	1.000	0.749	0.697	100.4

	OD	mean
1	0.049	0.052
2	0.055	
3	0.051	
4	0.054	
5	0.064	
6	0.062	
7	0.059	
8	0.026	
