# Supplementary material for: Effects of shinbuto and ninjinto on prostaglandin E2 production in lipopolysaccharide-treated human gingival fibroblasts
Source: PeerJ. 2017 Dec 1;5:e4120. doi: 10.7717/peerj.4120 (PMC5713626; doi:10.7717/peerj.4120)
Supplement: Data S1 [file peerj-05-4120-s001.zip › Fig2/006_PgLPS_TJ029_PGE2-1.pdf]

- Exp. 6
- Condition
  - drug1: PgLPS (pg/ml)
  - drug2: TJ029 (mg/ml)
  - experimental No. 1
  - treatment: 24h
- Measurement
  - PGE2
  - Date: 2012.7.6
- Cells
  - cells: HGFs (No. 1), passages: 15
  - cell numbers: 1×10^4 cells/well = 5×10^4 cells/ml

	conc.	OD
1	7.8	0.757
2	15.6	0.673
3	31.2	0.542
4	62.5	0.465
5	125.0	0.316
6	250.0	0.239
7	500.0	0.191
8	1000.0	0.170

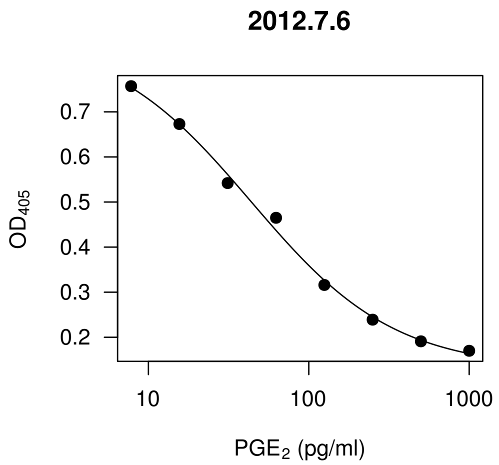

	drug1	drug2	mean	SD
1	0	0.000	0.029	0.002
2	0	0.010	0.028	0.007
3	0	0.100	0.027	0.002
4	0	1.000	0.033	0.003
5	10	0.000	0.414	0.151
6	10	0.010	0.474	0.084
7	10	0.100	0.420	0.059
8	10	1.000	0.430	0.021

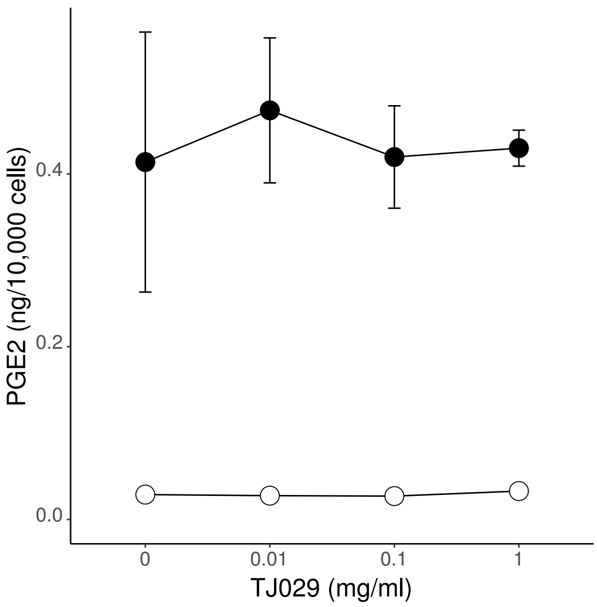

	drug1	drug2	viability	dilution	OD	conc. (pg/ml)	net (ng/ml)	(ng/10,000 cells)
1	0	0.000	103.89	5	0.570	29.61	0.148	0.029
2	0	0.000	95.67	5	0.569	29.78	0.149	0.031
3	0	0.000	100.43	5	0.587	26.83	0.134	0.027
4	0	0.010	104.18	5	0.634	20.14	0.101	0.019
5	0	0.010	96.68	5	0.560	31.36	0.157	0.032
6	0	0.010	99.28	5	0.564	30.65	0.153	0.031
7	0	0.100	104.04	5	0.595	25.59	0.128	0.025
8	0	0.100	95.67	5	0.584	27.30	0.137	0.029
9	0	0.100	98.85	5	0.582	27.62	0.138	0.028
10	0	1.000	101.15	5	0.534	36.32	0.182	0.036
11	0	1.000	96.54	5	0.559	31.54	0.158	0.033
12	0	1.000	100.43	5	0.567	30.13	0.151	0.030
13	10	0.000	100.14	5	0.187	564.56	2.823	0.564
14	10	0.000	101.44	5	0.204	420.51	2.103	0.415
15	10	0.000	99.42	5	0.241	261.23	1.306	0.263
16	10	0.010	98.13	5	0.204	420.51	2.103	0.429
17	10	0.010	99.71	5	0.204	420.51	2.103	0.422
18	10	0.010	98.99	5	0.187	564.56	2.823	0.570
19	10	0.100	100.29	5	0.202	433.87	2.169	0.433
20	10	0.100	101.01	5	0.215	358.42	1.792	0.355
21	10	0.100	98.27	5	0.198	462.99	2.315	0.471
22	10	1.000	98.99	5	0.200	448.01	2.240	0.453
23	10	1.000	102.16	5	0.204	420.51	2.103	0.412
24	10	1.000	100.43	5	0.203	427.09	2.135	0.425
