# Supplementary material for: Effects of shinbuto and ninjinto on prostaglandin E2 production in lipopolysaccharide-treated human gingival fibroblasts
Source: PeerJ. 2017 Dec 1;5:e4120. doi: 10.7717/peerj.4120 (PMC5713626; doi:10.7717/peerj.4120)
Supplement: Data S1 [file peerj-05-4120-s001.zip › Fig2/006_PgLPS_TJ041_WST-1.pdf]

- Exp. 6
- Condition
  - drug1: PgLPS (pg/ml)
  - drug2: TJ041 (mg/ml)
  - experimental No. 1
  - treatment: 24h
- Measurement
  - WST-8
  - Date: 2012.7.5
- Cells
  - cells: HGFs (No. 1), passages: 15
  - cell numbers: 1×10^4 cells/well

	drug1	drug2	mean	SD
1	0	0.000	100.0	3.3
2	0	0.010	98.0	2.7
3	0	0.100	99.4	3.5
4	0	1.000	100.3	2.6
5	10	0.000	101.5	1.3
6	10	0.010	101.1	0.9
7	10	0.100	102.3	1.9
8	10	1.000	102.0	1.6

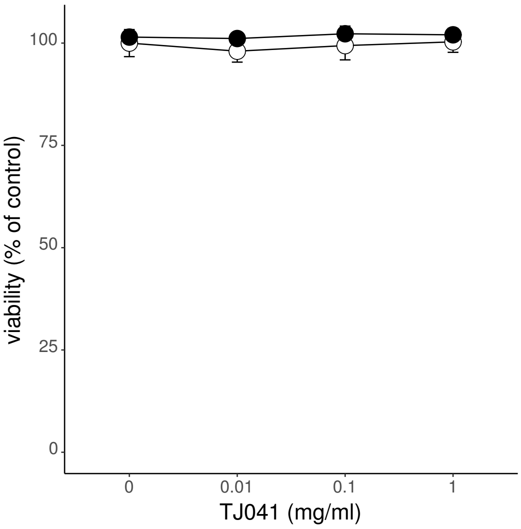

	OD	mean
1	0.059	0.058
2	0.048	
3	0.056	
4	0.055	
5	0.056	
6	0.077	
7	0.058	
8	0.054	

	drug1	drug2	OD	OD-blank	viability
1	0	0.000	0.734	0.676	102.7
2	0	0.000	0.692	0.634	96.3
3	0	0.000	0.723	0.665	101.0
4	0	0.010	0.713	0.655	99.5
5	0	0.010	0.683	0.625	94.9
6	0	0.010	0.714	0.656	99.6
7	0	0.100	0.735	0.677	102.8
8	0	0.100	0.689	0.631	95.8
9	0	0.100	0.713	0.655	99.5
10	0	1.000	0.726	0.668	101.5
11	0	1.000	0.699	0.641	97.4
12	0	1.000	0.730	0.672	102.1
13	10	0.000	0.729	0.671	101.9
14	10	0.000	0.733	0.675	102.5
15	10	0.000	0.716	0.658	99.9
16	10	0.010	0.719	0.661	100.4
17	10	0.010	0.730	0.672	102.1
18	10	0.010	0.722	0.664	100.9
19	10	0.100	0.721	0.663	100.7
20	10	0.100	0.745	0.687	104.4
21	10	0.100	0.728	0.670	101.8
22	10	1.000	0.721	0.663	100.7
23	10	1.000	0.741	0.683	103.7
24	10	1.000	0.727	0.669	101.6
