# Supplementary material for: Effects of shinbuto and ninjinto on prostaglandin E2 production in lipopolysaccharide-treated human gingival fibroblasts
Source: PeerJ. 2017 Dec 1;5:e4120. doi: 10.7717/peerj.4120 (PMC5713626; doi:10.7717/peerj.4120)
Supplement: Data S1 [file peerj-05-4120-s001.zip › Fig2/006_PgLPS_TJ041_IL-8-1.pdf]

- Exp. 6
- Condition
  - drug1: PgLPS (pg/ml)
  - drug2: TJ041 (mg/ml)
  - experimental No. 1
  - treatment: 24h
- Measurement
  - IL-8
  - Date: 2012.11.5
- Cells
  - cells: HGFs (No. 1), passages: 15
  - cell numbers: 1×10^4 cells/well = 5×10^4 cells/ml

	conc.	OD	OD-blank
1	0.0	0.073	0.000
2	15.6	0.122	0.049
3	31.2	0.154	0.081
4	62.5	0.227	0.154
5	125.0	0.347	0.274
6	250.0	0.571	0.498
7	500.0	0.875	0.802
8	1000.0	1.179	1.106

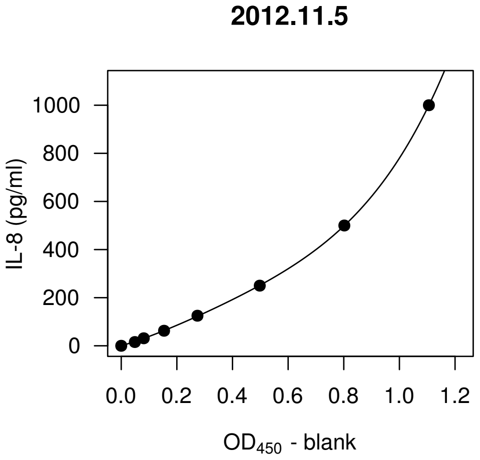

	drug1	drug2	mean	SD
1	0	0.000	0.060	0.028
2	0	0.010	0.049	0.019
3	0	0.100	0.049	0.029
4	0	1.000	0.046	0.012
5	10	0.000	2.837	0.412
6	10	0.010	3.054	0.296
7	10	0.100	3.379	0.238
8	10	1.000	3.603	0.203

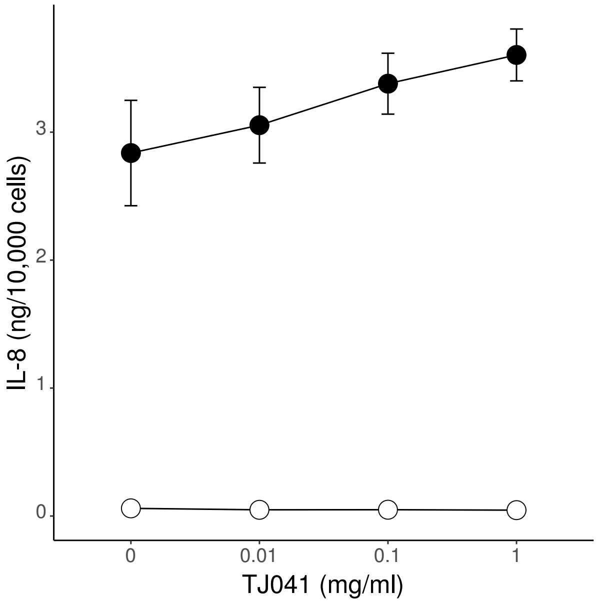

	drug1	drug2	viability	dilution	OD	conc. (pg/ml)	net (ng/ml)	(ng/10,000 cells)
1	0	0.000	102.68	50	0.093	6.98	0.349	0.068
2	0	0.000	96.30	50	0.081	2.74	0.137	0.028
3	0	0.000	101.01	50	0.097	8.43	0.422	0.084
4	0	0.010	99.49	50	0.085	4.14	0.207	0.042
5	0	0.010	94.94	50	0.092	6.62	0.331	0.070
6	0	0.010	99.65	50	0.083	3.44	0.172	0.034
7	0	0.100	102.83	50	0.079	2.05	0.102	0.020
8	0	0.100	95.85	50	0.087	4.84	0.242	0.051
9	0	0.100	99.49	50	0.095	7.71	0.385	0.077
10	0	1.000	101.47	50	0.090	5.91	0.295	0.058
11	0	1.000	97.37	50	0.086	4.49	0.224	0.046
12	0	1.000	102.08	50	0.083	3.44	0.172	0.034
13	10	0.000	101.92	50	0.616	280.44	14.022	2.752
14	10	0.000	102.53	50	0.575	253.70	12.685	2.474
15	10	0.000	99.95	50	0.684	328.28	16.414	3.284
16	10	0.010	100.40	50	0.611	277.10	13.855	2.760
17	10	0.010	102.08	50	0.661	311.54	15.577	3.052
18	10	0.010	100.86	50	0.697	338.03	16.901	3.351
19	10	0.100	100.71	50	0.671	318.74	15.937	3.165
20	10	0.100	104.35	50	0.749	379.34	18.967	3.635
21	10	0.100	101.77	50	0.699	339.55	16.977	3.336
22	10	1.000	100.71	50	0.709	347.22	17.361	3.448
23	10	1.000	103.75	50	0.733	366.20	18.310	3.530
24	10	1.000	101.62	50	0.761	389.47	19.473	3.833
