# Supplementary material for: Effects of shinbuto and ninjinto on prostaglandin E2 production in lipopolysaccharide-treated human gingival fibroblasts
Source: PeerJ. 2017 Dec 1;5:e4120. doi: 10.7717/peerj.4120 (PMC5713626; doi:10.7717/peerj.4120)
Supplement: Data S1 [file peerj-05-4120-s001.zip › Fig2/006_PgLPS_TJ032_IL-6-1.pdf]

- Exp. 6
- Condition
  - drug1: PgLPS (pg/ml)
  - drug2: TJ030 (mg/ml)
  - experimental No. 1
  - treatment: 24h
- Measurement
  - IL-6
  - Date: 2013.3.12
- Cells
  - cells: HGFs (No. 1), passages: 15
  - cell numbers: 1×10^4 cells/well = 5×10^4 cells/ml

	conc.	OD	OD-blank
1	0.0	0.075	0.000
2	15.6	0.097	0.022
3	31.2	0.122	0.047
4	62.5	0.188	0.113
5	125.0	0.308	0.233
6	250.0	0.382	0.307
7	500.0	0.488	0.413
8	1000.0	0.650	0.575

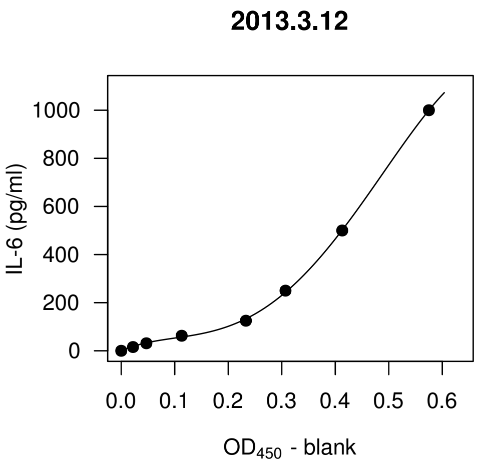

	drug1	drug2	mean	SD
1	0	0.000	0.001	0.002
2	0	0.010	0.008	0.004
3	0	0.100	0.006	0.006
4	0	1.000	0.072	0.025
5	10	0.000	0.436	0.133
6	10	0.010	0.536	0.050
7	10	0.100	1.114	0.237
8	10	1.000	0.972	0.269

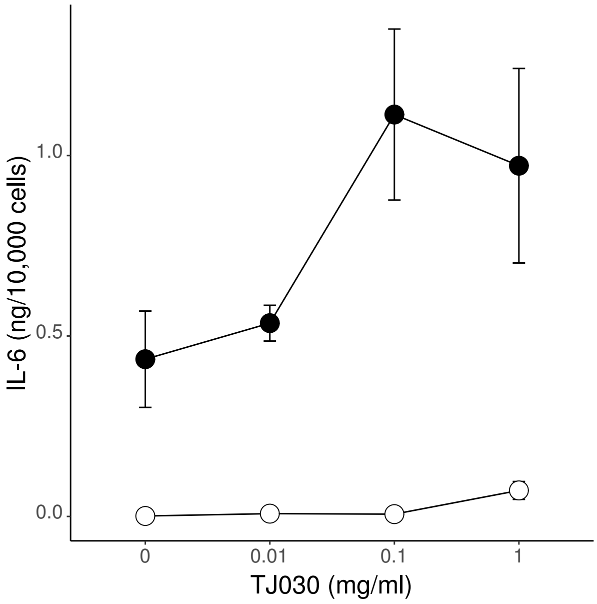

	drug1	drug2	viability	dilution	OD	conc. (pg/ml)	net (ng/ml)	(ng/10,000 cells)
1	0	0.000	102.23	20	0.076	0.97	0.019	0.004
2	0	0.000	96.79	20	0.075	0.00	0.000	0.000
3	0	0.000	100.99	20	0.075	0.00	0.000	0.000
4	0	0.010	102.23	20	0.076	0.97	0.019	0.004
5	0	0.010	95.23	20	0.078	2.86	0.057	0.012
6	0	0.010	100.67	20	0.077	1.92	0.038	0.008
7	0	0.100	104.41	20	0.075	0.00	0.000	0.000
8	0	0.100	97.56	20	0.078	2.86	0.057	0.012
9	0	0.100	101.45	20	0.077	1.92	0.038	0.008
10	0	1.000	104.10	20	0.109	26.05	0.521	0.100
11	0	1.000	99.27	20	0.090	13.12	0.262	0.053
12	0	1.000	101.92	20	0.094	16.15	0.323	0.063
13	10	0.000	103.16	20	0.282	107.49	2.150	0.417
14	10	0.000	104.25	20	0.323	150.53	3.011	0.578
15	10	0.000	102.70	20	0.243	80.26	1.605	0.313
16	10	0.010	105.18	20	0.321	148.05	2.961	0.563
17	10	0.010	103.01	20	0.319	145.60	2.912	0.565
18	10	0.010	103.16	20	0.299	123.36	2.467	0.478
19	10	0.100	101.76	20	0.432	351.66	7.033	1.382
20	10	0.100	103.32	20	0.381	241.44	4.829	0.935
21	10	0.100	101.92	20	0.391	260.85	5.217	1.024
22	10	1.000	101.61	20	0.400	279.25	5.585	1.099
23	10	1.000	104.25	20	0.410	300.73	6.015	1.154
24	10	1.000	103.01	20	0.338	170.54	3.411	0.662
