# Supplementary material for: Effects of shinbuto and ninjinto on prostaglandin E2 production in lipopolysaccharide-treated human gingival fibroblasts
Source: PeerJ. 2017 Dec 1;5:e4120. doi: 10.7717/peerj.4120 (PMC5713626; doi:10.7717/peerj.4120)
Supplement: Data S1 [file peerj-05-4120-s001.zip › Fig2/006_PgLPS_TJ029_IL-6-1.pdf]

- Exp. 6
- Condition
  - drug1: PgLPS (pg/ml)
  - drug2: TJ029 (mg/ml)
  - experimental No. 1
  - treatment: 24h
- Measurement
  - IL-6
  - Date: 2013.11.1
- Cells
  - cells: HGFs (No. 1), passages: 15
  - cell numbers: 1×10^4 cells/well = 5×10^4 cells/ml

	conc.	OD	OD-blank
1	0	0.063	0.000
2	125	0.220	0.157
3	250	0.318	0.255
4	500	0.492	0.429
5	1000	0.919	0.856

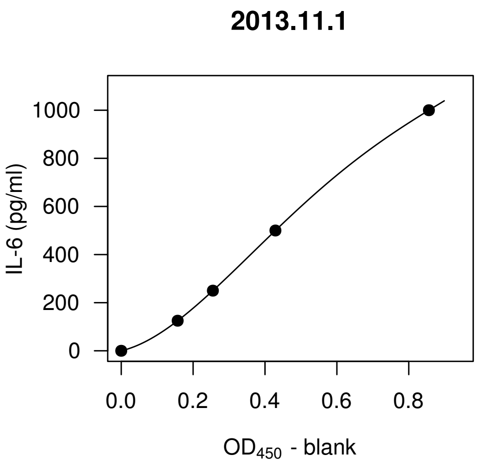

	drug1	drug2	mean	SD
1	0	0.000	0.009	0.002
2	0	0.010	0.008	0.004
3	0	0.100	0.011	0.001
4	0	1.000	0.014	0.001
5	10	0.000	1.521	0.226
6	10	0.010	1.541	0.134
7	10	0.100	1.680	0.148
8	10	1.000	1.806	0.025

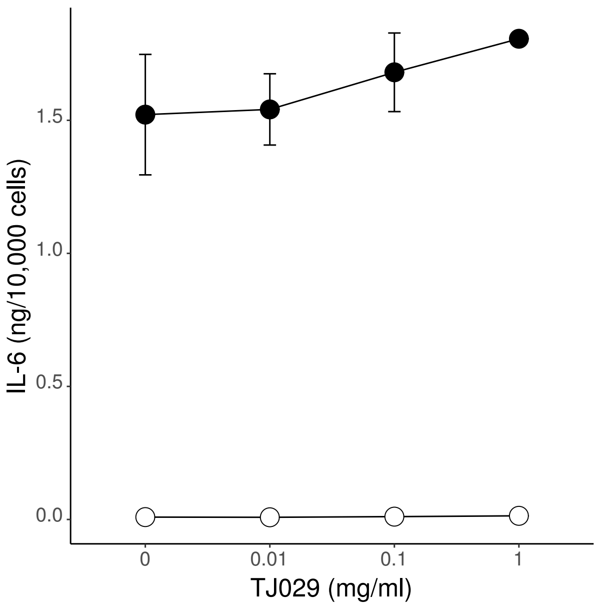

	drug1	drug2	viability	dilution	OD	conc. (pg/ml)	net (ng/ml)	(ng/10,000 cells)
1	0	0.000	103.89	10	0.073	3.85	0.039	0.007
2	0	0.000	95.67	10	0.073	3.85	0.039	0.008
3	0	0.000	100.43	10	0.077	5.58	0.056	0.011
4	0	0.010	104.18	10	0.069	2.23	0.022	0.004
5	0	0.010	96.68	10	0.072	3.43	0.034	0.007
6	0	0.010	99.28	10	0.079	6.49	0.065	0.013
7	0	0.100	104.04	10	0.078	6.03	0.060	0.012
8	0	0.100	95.67	10	0.076	5.14	0.051	0.011
9	0	0.100	98.85	10	0.075	4.70	0.047	0.010
10	0	1.000	101.15	10	0.080	6.95	0.069	0.014
11	0	1.000	96.54	10	0.078	6.03	0.060	0.012
12	0	1.000	100.43	10	0.081	7.42	0.074	0.015
13	10	0.000	100.14	10	0.654	718.94	7.189	1.436
14	10	0.000	101.44	10	0.627	684.76	6.848	1.350
15	10	0.000	99.42	10	0.799	883.82	8.838	1.778
16	10	0.010	98.13	10	0.735	814.90	8.149	1.661
17	10	0.010	99.71	10	0.705	780.52	7.805	1.566
18	10	0.010	98.99	10	0.632	691.17	6.912	1.396
19	10	0.100	100.29	10	0.748	829.37	8.294	1.654
20	10	0.100	101.01	10	0.844	929.05	9.290	1.840
21	10	0.100	98.27	10	0.688	760.43	7.604	1.548
22	10	1.000	98.99	10	0.813	898.15	8.982	1.815
23	10	1.000	102.16	10	0.823	908.24	9.082	1.778
24	10	1.000	100.43	10	0.832	917.22	9.172	1.827
