# Supplementary material for: Effects of shinbuto and ninjinto on prostaglandin E2 production in lipopolysaccharide-treated human gingival fibroblasts
Source: PeerJ. 2017 Dec 1;5:e4120. doi: 10.7717/peerj.4120 (PMC5713626; doi:10.7717/peerj.4120)
Supplement: Data S1 [file peerj-05-4120-s001.zip › Fig2/006_PgLPS_TJ030_IL-6-1.pdf]

- Exp. 6
- Condition
  - drug1: PgLPS (pg/ml)
  - drug2: TJ030 (mg/ml)
  - experimental No. 1
  - treatment: 24h
- Measurement
  - IL-6
  - Date: 2013.3.12
- Cells
  - cells: HGFs (No. 1), passages: 15
  - cell numbers: $1 \times 10^4$ cells/well = $5 \times 10^4$ cells/ml

| | conc. | OD | OD-blank |
|---|---|---|---|
| 1 | 0.0 | 0.078 | 0.000 |
| 2 | 15.6 | 0.097 | 0.019 |
| 3 | 31.2 | 0.122 | 0.044 |
| 4 | 62.5 | 0.188 | 0.110 |
| 5 | 125.0 | 0.308 | 0.230 |
| 6 | 250.0 | 0.382 | 0.304 |
| 7 | 500.0 | 0.488 | 0.410 |
| 8 | 1000.0 | 0.650 | 0.572 |

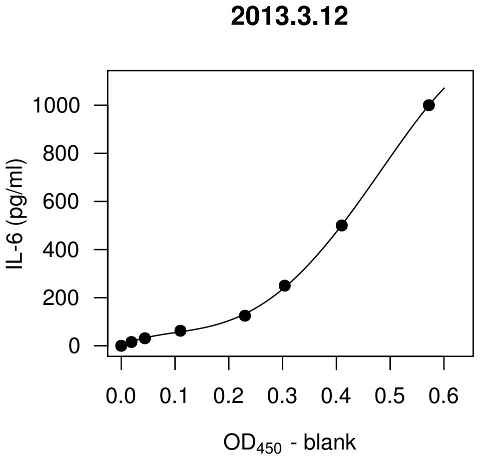


| | drug1 | drug2 | mean | SD |
|---|---|---|---|---|
| 1 | 0 | 0.000 | 0.008 | 0.007 |
| 2 | 0 | 0.010 | 0.008 | 0.014 |
| 3 | 0 | 0.100 | 0.021 | 0.018 |
| 4 | 0 | 1.000 | 0.035 | 0.011 |
| 5 | 10 | 0.000 | 0.784 | 0.185 |
| 6 | 10 | 0.010 | 0.803 | 0.163 |
| 7 | 10 | 0.100 | 1.294 | 0.053 |
| 8 | 10 | 1.000 | 1.278 | 0.291 |

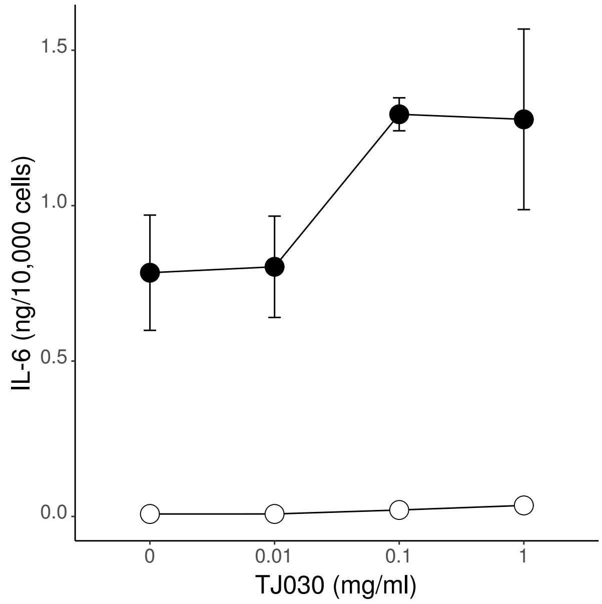

| | drug1 | drug2 | viability | dilution | OD | conc. (pg/ml) | net (ng/ml) | (ng/10,000 cells) |
|---|---|---|---|---|---|---|---|---|
| 1 | 0 | 0.000 | 103.89 | 20 | 0.081 | 2.97 | 0.059 | 0.011 |
| 2 | 0 | 0.000 | 95.67 | 20 | 0.081 | 2.97 | 0.059 | 0.012 |
| 3 | 0 | 0.000 | 100.43 | 20 | 0.078 | 0.00 | 0.000 | 0.000 |
| 4 | 0 | 0.010 | 104.18 | 20 | 0.078 | 0.00 | 0.000 | 0.000 |
| 5 | 0 | 0.010 | 96.68 | 20 | 0.084 | 5.82 | 0.116 | 0.024 |
| 6 | 0 | 0.010 | 99.28 | 20 | 0.078 | 0.00 | 0.000 | 0.000 |
| 7 | 0 | 0.100 | 104.04 | 20 | 0.083 | 4.89 | 0.098 | 0.019 |
| 8 | 0 | 0.100 | 95.67 | 20 | 0.088 | 9.43 | 0.189 | 0.039 |
| 9 | 0 | 0.100 | 98.85 | 20 | 0.079 | 1.01 | 0.020 | 0.004 |
| 10 | 0 | 1.000 | 101.15 | 20 | 0.084 | 5.82 | 0.116 | 0.023 |
| 11 | 0 | 1.000 | 96.54 | 20 | 0.088 | 9.43 | 0.189 | 0.039 |
| 12 | 0 | 1.000 | 100.43 | 20 | 0.090 | 11.15 | 0.223 | 0.044 |
| 13 | 10 | 0.000 | 100.14 | 20 | 0.369 | 219.14 | 4.383 | 0.875 |
| 14 | 10 | 0.000 | 101.44 | 20 | 0.375 | 229.85 | 4.597 | 0.906 |
| 15 | 10 | 0.000 | 99.42 | 20 | 0.316 | 141.88 | 2.838 | 0.571 |
| 16 | 10 | 0.010 | 98.13 | 20 | 0.327 | 155.41 | 3.108 | 0.633 |
| 17 | 10 | 0.010 | 99.71 | 20 | 0.360 | 203.84 | 4.077 | 0.818 |
| 18 | 10 | 0.010 | 98.99 | 20 | 0.379 | 237.21 | 4.744 | 0.959 |
| 19 | 10 | 0.100 | 100.29 | 20 | 0.427 | 339.31 | 6.786 | 1.353 |
| 20 | 10 | 0.100 | 101.01 | 20 | 0.417 | 316.00 | 6.320 | 1.251 |
| 21 | 10 | 0.100 | 98.27 | 20 | 0.416 | 313.72 | 6.274 | 1.277 |
| 22 | 10 | 1.000 | 98.99 | 20 | 0.450 | 396.77 | 7.935 | 1.603 |
| 23 | 10 | 1.000 | 102.16 | 20 | 0.411 | 302.51 | 6.050 | 1.184 |
| 24 | 10 | 1.000 | 100.43 | 20 | 0.392 | 262.37 | 5.247 | 1.045 |
