# Supplementary material for: Effects of shinbuto and ninjinto on prostaglandin E2 production in lipopolysaccharide-treated human gingival fibroblasts
Source: PeerJ. 2017 Dec 1;5:e4120. doi: 10.7717/peerj.4120 (PMC5713626; doi:10.7717/peerj.4120)
Supplement: Data S1 [file peerj-05-4120-s001.zip › Fig2/006_PgLPS_TJ030_WST-1.pdf]

- Exp. 6
- Condition
  - drug1: PgLPS (pg/ml)
  - drug2: TJ030 (mg/ml)
  - experimental No. 1
  - treatment: 24h
- Measurement
  - WST-8
  - Date: 2012.7.5
- Cells
  - cells: HGFs (No. 1), passages: 15
  - cell numbers: $1 \times 10^4$ cells/well

| | drug1 | drug2 | mean | SD |
|---|---|---|---|---|
| 1 | 0 | 0.000 | 100.0 | 3.6 |
| 2 | 0 | 0.010 | 99.4 | 3.7 |
| 3 | 0 | 0.100 | 99.9 | 4.0 |
| 4 | 0 | 1.000 | 100.1 | 2.8 |
| 5 | 10 | 0.000 | 100.8 | 0.9 |
| 6 | 10 | 0.010 | 99.0 | 0.4 |
| 7 | 10 | 0.100 | 100.1 | 2.7 |
| 8 | 10 | 1.000 | 100.4 | 1.5 |

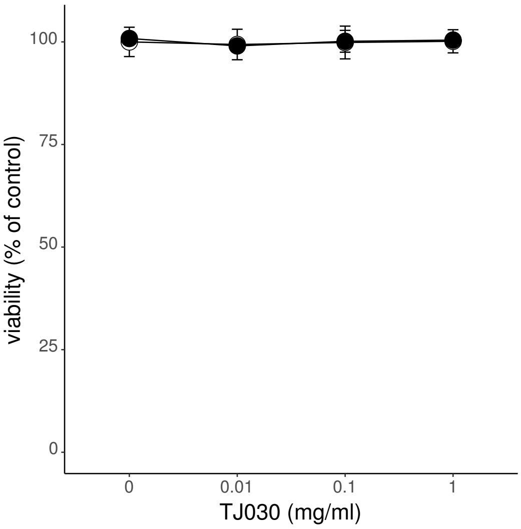


| | OD | mean |
|---|---|---|
| 1 | 0.054 | 0.057 |
| 2 | 0.048 | |
| 3 | 0.055 | |
| 4 | 0.054 | |
| 5 | 0.055 | |
| 6 | 0.075 | |
| 7 | 0.057 | |
| 8 | 0.056 | |

| | drug1 | drug2 | OD | OD-blank | viability |
|---|---|---|---|---|---|
| 1 | 0 | 0.000 | 0.762 | 0.705 | 103.1 |
| 2 | 0 | 0.000 | 0.714 | 0.657 | 96.1 |
| 3 | 0 | 0.000 | 0.746 | 0.689 | 100.8 |
| 4 | 0 | 0.010 | 0.764 | 0.707 | 103.4 |
| 5 | 0 | 0.010 | 0.714 | 0.657 | 96.1 |
| 6 | 0 | 0.010 | 0.731 | 0.674 | 98.6 |
| 7 | 0 | 0.100 | 0.770 | 0.713 | 104.3 |
| 8 | 0 | 0.100 | 0.717 | 0.660 | 96.5 |
| 9 | 0 | 0.100 | 0.732 | 0.675 | 98.7 |
| 10 | 0 | 1.000 | 0.757 | 0.700 | 102.4 |
| 11 | 0 | 1.000 | 0.720 | 0.663 | 97.0 |
| 12 | 0 | 1.000 | 0.748 | 0.691 | 101.1 |
| 13 | 10 | 0.000 | 0.747 | 0.690 | 100.9 |
| 14 | 10 | 0.000 | 0.752 | 0.695 | 101.7 |
| 15 | 10 | 0.000 | 0.740 | 0.683 | 99.9 |
| 16 | 10 | 0.010 | 0.731 | 0.674 | 98.6 |
| 17 | 10 | 0.010 | 0.734 | 0.677 | 99.0 |
| 18 | 10 | 0.010 | 0.737 | 0.680 | 99.5 |
| 19 | 10 | 0.100 | 0.727 | 0.670 | 98.0 |
| 20 | 10 | 0.100 | 0.762 | 0.705 | 103.1 |
| 21 | 10 | 0.100 | 0.736 | 0.679 | 99.3 |
| 22 | 10 | 1.000 | 0.734 | 0.677 | 99.0 |
| 23 | 10 | 1.000 | 0.754 | 0.697 | 101.9 |
| 24 | 10 | 1.000 | 0.743 | 0.686 | 100.3 |
