# Supplementary material for: Effects of shinbuto and ninjinto on prostaglandin E2 production in lipopolysaccharide-treated human gingival fibroblasts
Source: PeerJ. 2017 Dec 1;5:e4120. doi: 10.7717/peerj.4120 (PMC5713626; doi:10.7717/peerj.4120)
Supplement: Data S1 [file peerj-05-4120-s001.zip › Fig2/006_PgLPS_TJ030_IL-8-1.pdf]

- Exp. 6
- Condition
  - drug1: PgLPS (pg/ml)
  - drug2: TJ030 (mg/ml)
  - experimental No. 1
  - treatment: 24h
- Measurement
  - IL-8
  - Date: 2012.11.5
- Cells
  - cells: HGFs (No. 1), passages: 15
  - cell numbers: 1×10^4 cells/well = 5×10^4 cells/ml

	conc.	OD	OD-blank
1	0.0	0.053	0.000
2	15.6	0.092	0.039
3	31.2	0.120	0.067
4	62.5	0.188	0.135
5	125.0	0.295	0.242
6	250.0	0.493	0.440
7	500.0	0.793	0.740
8	1000.0	1.079	1.026

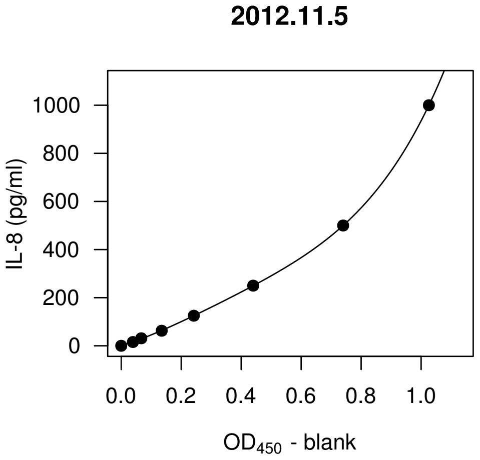

	drug1	drug2	mean	SD
1	0	0.000	0.070	0.012
2	0	0.010	0.032	0.015
3	0	0.100	0.020	0.011
4	0	1.000	0.051	0.005
5	10	0.000	3.520	0.395
6	10	0.010	3.678	0.149
7	10	0.100	3.396	0.113
8	10	1.000	3.398	0.118

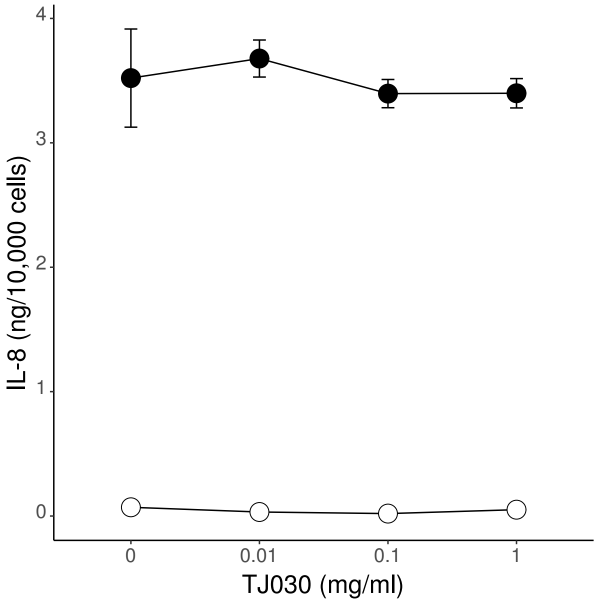

	drug1	drug2	viability	dilution	OD	conc. (pg/ml)	net (ng/ml)	(ng/10,000 cells)
1	0	0.000	103.12	50	0.071	7.14	0.357	0.069
2	0	0.000	96.10	50	0.073	7.96	0.398	0.083
3	0	0.000	100.78	50	0.068	5.91	0.296	0.059
4	0	0.010	103.41	50	0.066	5.10	0.255	0.049
5	0	0.010	96.10	50	0.059	2.32	0.116	0.024
6	0	0.010	98.59	50	0.059	2.32	0.116	0.024
7	0	0.100	104.29	50	0.055	0.77	0.038	0.007
8	0	0.100	96.54	50	0.059	2.32	0.116	0.024
9	0	0.100	98.73	50	0.060	2.71	0.136	0.027
10	0	1.000	102.39	50	0.065	4.70	0.235	0.046
11	0	1.000	96.98	50	0.066	5.10	0.255	0.053
12	0	1.000	101.07	50	0.067	5.51	0.275	0.054
13	10	0.000	100.93	50	0.579	309.46	15.473	3.066
14	10	0.000	101.66	50	0.674	384.00	19.200	3.777
15	10	0.000	99.90	50	0.659	371.40	18.570	3.718
16	10	0.010	98.59	50	0.646	360.77	18.039	3.659
17	10	0.010	99.03	50	0.669	379.76	18.988	3.835
18	10	0.010	99.46	50	0.635	351.96	17.598	3.539
19	10	0.100	98.00	50	0.611	333.31	16.665	3.401
20	10	0.100	103.12	50	0.647	361.58	18.079	3.506
21	10	0.100	99.32	50	0.601	325.74	16.287	3.280
22	10	1.000	99.03	50	0.603	327.24	16.362	3.304
23	10	1.000	101.95	50	0.645	359.96	17.998	3.531
24	10	1.000	100.34	50	0.616	337.13	16.857	3.360
