# Supplementary material for: Effects of shinbuto and ninjinto on prostaglandin E2 production in lipopolysaccharide-treated human gingival fibroblasts
Source: PeerJ. 2017 Dec 1;5:e4120. doi: 10.7717/peerj.4120 (PMC5713626; doi:10.7717/peerj.4120)
Supplement: Data S1 [file peerj-05-4120-s001.zip › Fig2/006_PgLPS_TJ041_IL-6-1.pdf]

- Exp. 6
- Condition
  - drug1: PgLPS (pg/ml)
  - drug2: TJ041 (mg/ml)
  - experimental No. 1
  - treatment: 24h
- Measurement
  - IL-6
  - Date: 2013.11.1
- Cells
  - cells: HGFs (No. 1), passages: 15
  - cell numbers: 1×10^4 cells/well = 5×10^4 cells/ml

	conc.	OD	OD-blank
1	0	0.063	0.000
2	125	0.220	0.157
3	250	0.318	0.255
4	500	0.492	0.429
5	1000	0.919	0.856

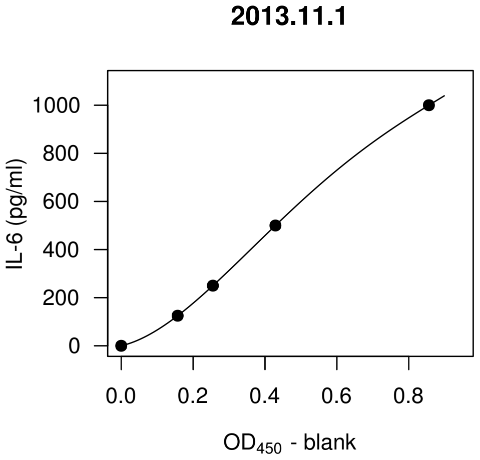

	drug1	drug2	mean	SD
1	0	0.000	0.009	0.001
2	0	0.010	0.008	0.001
3	0	0.100	0.010	0.002
4	0	1.000	0.017	0.004
5	10	0.000	1.122	0.066
6	10	0.010	1.539	0.093
7	10	0.100	1.750	0.119
8	10	1.000	1.776	0.226

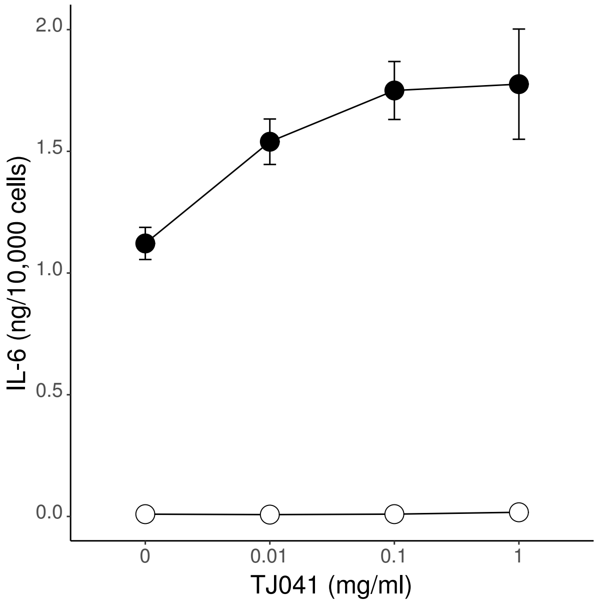

	drug1	drug2	viability	dilution	OD	conc. (pg/ml)	net (ng/ml)	(ng/10,000 cells)
1	0	0.000	102.68	10	0.076	5.14	0.051	0.010
2	0	0.000	96.30	10	0.073	3.85	0.039	0.008
3	0	0.000	101.01	10	0.076	5.14	0.051	0.010
4	0	0.010	99.49	10	0.072	3.43	0.034	0.007
5	0	0.010	94.94	10	0.072	3.43	0.034	0.007
6	0	0.010	99.65	10	0.074	4.27	0.043	0.009
7	0	0.100	102.83	10	0.073	3.85	0.039	0.007
8	0	0.100	95.85	10	0.076	5.14	0.051	0.011
9	0	0.100	99.49	10	0.076	5.14	0.051	0.010
10	0	1.000	101.47	10	0.079	6.49	0.065	0.013
11	0	1.000	97.37	10	0.087	10.37	0.104	0.021
12	0	1.000	102.08	10	0.084	8.86	0.089	0.017
13	10	0.000	101.92	10	0.547	577.67	5.777	1.134
14	10	0.000	102.53	10	0.519	538.46	5.385	1.050
15	10	0.000	99.95	10	0.556	590.11	5.901	1.181
16	10	0.010	100.40	10	0.710	786.35	7.863	1.566
17	10	0.010	102.08	10	0.744	824.95	8.249	1.616
18	10	0.010	100.86	10	0.658	723.91	7.239	1.435
19	10	0.100	100.71	10	0.770	853.31	8.533	1.695
20	10	0.100	104.35	10	0.902	984.28	9.843	1.886
21	10	0.100	101.77	10	0.766	849.01	8.490	1.668
22	10	1.000	100.71	10	0.894	976.82	9.768	1.940
23	10	1.000	103.75	10	0.887	970.26	9.703	1.870
24	10	1.000	101.62	10	0.697	771.12	7.711	1.518
