# Supplementary material for: Effects of shinbuto and ninjinto on prostaglandin E2 production in lipopolysaccharide-treated human gingival fibroblasts
Source: PeerJ. 2017 Dec 1;5:e4120. doi: 10.7717/peerj.4120 (PMC5713626; doi:10.7717/peerj.4120)
Supplement: Data S1 [file peerj-05-4120-s001.zip › Fig2/006_PgLPS_TJ032_PGE2-1.pdf]

- Exp. 6
- Condition
  - drug1: PgLPS (pg/ml)
  - drug2: TJ032 (mg/ml)
  - experimental No. 1
  - treatment: 24h
- Measurement
  - PGE2
  - Date: 2012.7.6
- Cells
  - cells: HGFs (No. 1), passages: 15
  - cell numbers: 1×10^4 cells/well = 5×10^4 cells/ml

	conc.	OD
1	7.8	0.802
2	15.6	0.726
3	31.2	0.707
4	62.5	0.580
5	125.0	0.428
6	250.0	0.318
7	500.0	0.243
8	1000.0	0.191

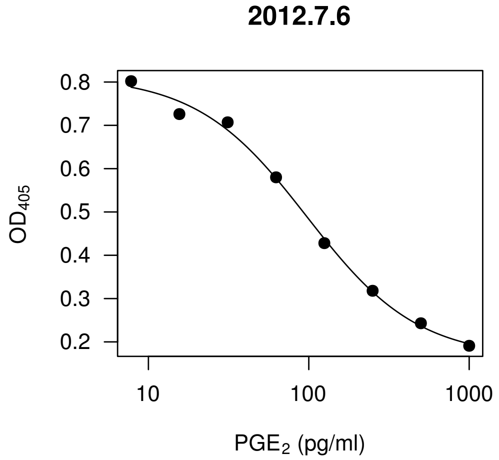

	drug1	drug2	mean	SD
1	0	0.000	0.017	0.004
2	0	0.010	0.018	0.007
3	0	0.100	0.010	0.001
4	0	1.000	0.006	0.001
5	10	0.000	0.449	0.043
6	10	0.010	0.359	0.041
7	10	0.100	0.291	0.027
8	10	1.000	0.061	0.024

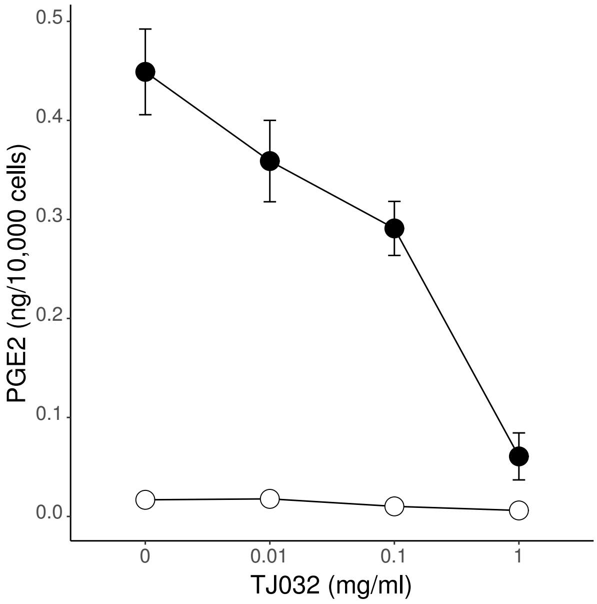

	drug1	drug2	viability	dilution	OD	conc. (pg/ml)	net (ng/ml)	(ng/10,000 cells)
1	0	0.000	102.23	5	0.737	19.80	0.099	0.019
2	0	0.000	96.79	5	0.742	18.64	0.093	0.019
3	0	0.000	100.99	5	0.771	11.90	0.060	0.012
4	0	0.010	102.23	5	0.773	11.43	0.057	0.011
5	0	0.010	95.23	5	0.719	24.00	0.120	0.025
6	0	0.010	100.67	5	0.749	17.03	0.085	0.017
7	0	0.100	104.41	5	0.776	10.72	0.054	0.010
8	0	0.100	97.56	5	0.783	9.03	0.045	0.009
9	0	0.100	101.45	5	0.774	11.20	0.056	0.011
10	0	1.000	104.10	5	0.794	6.28	0.031	0.006
11	0	1.000	99.27	5	0.798	5.23	0.026	0.005
12	0	1.000	101.92	5	0.791	7.05	0.035	0.007
13	10	0.000	103.16	5	0.238	490.75	2.454	0.476
14	10	0.000	104.25	5	0.253	416.05	2.080	0.399
15	10	0.000	102.70	5	0.239	484.97	2.425	0.472
16	10	0.010	105.18	5	0.251	424.71	2.124	0.404
17	10	0.010	103.01	5	0.268	360.44	1.802	0.350
18	10	0.010	103.16	5	0.277	333.30	1.667	0.323
19	10	0.100	101.76	5	0.296	286.69	1.433	0.282
20	10	0.100	103.32	5	0.300	278.33	1.392	0.269
21	10	0.100	101.92	5	0.279	327.77	1.639	0.322
22	10	1.000	101.61	5	0.637	44.64	0.223	0.044
23	10	1.000	104.25	5	0.610	52.35	0.262	0.050
24	10	1.000	103.01	5	0.504	90.45	0.452	0.088
