# Supplementary material for: Effects of shinbuto and ninjinto on prostaglandin E2 production in lipopolysaccharide-treated human gingival fibroblasts
Source: PeerJ. 2017 Dec 1;5:e4120. doi: 10.7717/peerj.4120 (PMC5713626; doi:10.7717/peerj.4120)
Supplement: Data S1 [file peerj-05-4120-s001.zip › Fig2/006_PgLPS_TJ041_PGE2-1.pdf]

- Exp. 6
- Condition
  - drug1: PgLPS (pg/ml)
  - drug2: TJ041 (mg/ml)
  - experimental No. 1
  - treatment: 24h
- Measurement
  - PGE2
  - Date: 2012.7.6
- Cells
  - cells: HGFs (No. 1), passages: 15
  - cell numbers: 1×10^4 cells/well = 5×10^4 cells/ml

	conc.	OD
1	7.8	0.802
2	15.6	0.726
3	31.2	0.707
4	62.5	0.580
5	125.0	0.428
6	250.0	0.318
7	500.0	0.243
8	1000.0	0.191

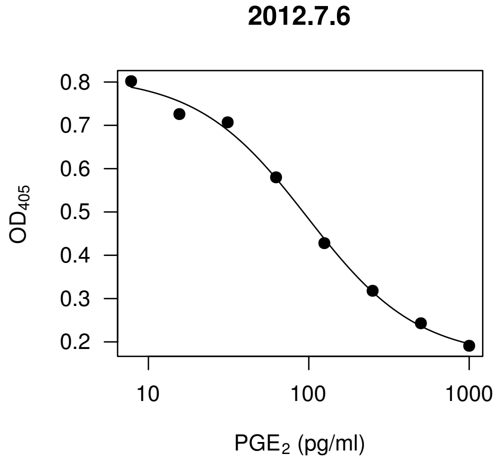

	drug1	drug2	mean	SD
1	0	0.000	0.024	0.014
2	0	0.010	0.024	0.009
3	0	0.100	0.023	0.009
4	0	1.000	0.028	0.007
5	10	0.000	0.456	0.060
6	10	0.010	0.469	0.037
7	10	0.100	0.449	0.073
8	10	1.000	0.335	0.076

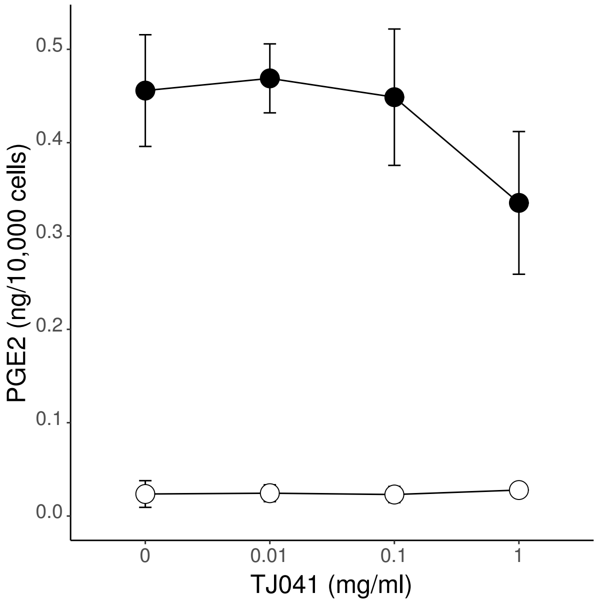

	drug1	drug2	viability	dilution	OD	conc. (pg/ml)	net (ng/ml)	(ng/10,000 cells)
1	0	0.000	102.68	5	0.691	30.70	0.153	0.030
2	0	0.000	96.30	5	0.684	32.42	0.162	0.034
3	0	0.000	101.01	5	0.790	7.30	0.037	0.007
4	0	0.010	99.49	5	0.718	24.23	0.121	0.024
5	0	0.010	94.94	5	0.759	14.71	0.074	0.015
6	0	0.010	99.65	5	0.680	33.41	0.167	0.034
7	0	0.100	102.83	5	0.682	32.91	0.165	0.032
8	0	0.100	95.85	5	0.730	21.43	0.107	0.022
9	0	0.100	99.49	5	0.759	14.71	0.074	0.015
10	0	1.000	101.47	5	0.712	25.65	0.128	0.025
11	0	1.000	97.37	5	0.674	34.91	0.175	0.036
12	0	1.000	102.08	5	0.724	22.83	0.114	0.022
13	10	0.000	101.92	5	0.246	447.96	2.240	0.440
14	10	0.000	102.53	5	0.253	416.05	2.080	0.406
15	10	0.000	99.95	5	0.233	521.83	2.609	0.522
16	10	0.010	100.40	5	0.250	429.17	2.146	0.427
17	10	0.010	102.08	5	0.238	490.75	2.454	0.481
18	10	0.010	100.86	5	0.236	502.73	2.514	0.498
19	10	0.100	100.71	5	0.266	367.03	1.835	0.364
20	10	0.100	104.35	5	0.235	508.94	2.545	0.488
21	10	0.100	101.77	5	0.236	502.73	2.514	0.494
22	10	1.000	100.71	5	0.299	280.38	1.402	0.278
23	10	1.000	103.75	5	0.283	317.20	1.586	0.306
24	10	1.000	101.62	5	0.250	429.17	2.146	0.422
