# Supplementary material for: Effects of shinbuto and ninjinto on prostaglandin E2 production in lipopolysaccharide-treated human gingival fibroblasts
Source: PeerJ. 2017 Dec 1;5:e4120. doi: 10.7717/peerj.4120 (PMC5713626; doi:10.7717/peerj.4120)
Supplement: Data S1 [file peerj-05-4120-s001.zip › Fig3/023_TJ-30-32_PGE2-1.pdf]

- Exp. 23
- Condition
  - drug1: TJ-30-32 ()
  - experimental No. 1
  - treatment: 24h
- Measurement
  - PGE2
  - Date: 2016.6.18
- Cells
  - cells: HGFs (No. 4), passages: 17
  - cell numbers: $1 \times 10^4$ cells/well = $5 \times 10^4$ cells/ml

| | conc. | OD |
|---|---|---|
| 1 | 7.8 | 1.070 |
| 2 | 15.6 | 0.943 |
| 3 | 31.2 | 0.879 |
| 4 | 62.5 | 0.755 |
| 5 | 125.0 | 0.529 |
| 6 | 250.0 | 0.362 |
| 7 | 500.0 | 0.292 |
| 8 | 1000.0 | 0.223 |

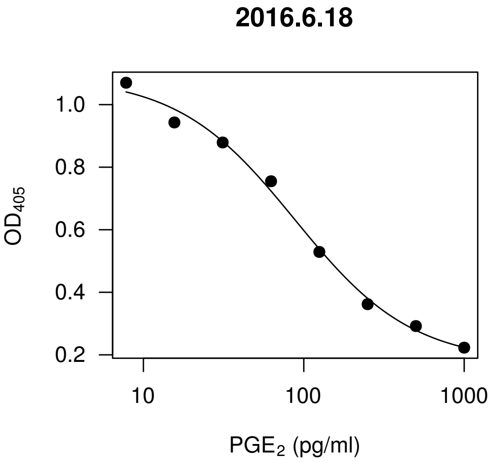


| | drug1 | mean | SD |
|---|---|---|---|
| 1 | 1 | 0.192 | 0.058 |
| 2 | 2 | 0.995 | 0.069 |
| 3 | 3 | 1.032 | 0.176 |
| 4 | 4 | 1.109 | 0.172 |

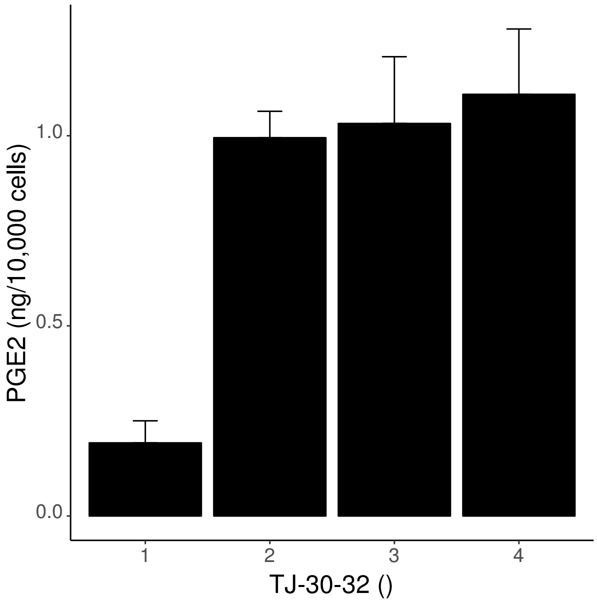

| | drug1 | viability | dilution | OD | conc. (pg/ml) | net (ng/ml) | (ng/10,000 cells) |
|---|---|---|---|---|---|---|---|
| 1 | 1 | 121.64 | 25 | 0.849 | 36.89 | 0.922 | 0.152 |
| 2 | 1 | 120.57 | 25 | 0.705 | 67.10 | 1.677 | 0.278 |
| 3 | 1 | 119.49 | 25 | 0.824 | 41.39 | 1.035 | 0.173 |
| 4 | 1 | 107.15 | 25 | 0.856 | 35.67 | 0.892 | 0.166 |
| 5 | 2 | 100.18 | 25 | 0.416 | 209.28 | 5.232 | 1.045 |
| 6 | 2 | 105.19 | 25 | 0.402 | 223.97 | 5.599 | 1.065 |
| 7 | 2 | 95.89 | 25 | 0.449 | 180.01 | 4.500 | 0.939 |
| 8 | 2 | 98.75 | 25 | 0.444 | 184.03 | 4.601 | 0.932 |
| 9 | 3 | 106.80 | 25 | 0.366 | 270.50 | 6.763 | 1.266 |
| 10 | 3 | 137.20 | 25 | 0.391 | 236.71 | 5.918 | 0.863 |
| 11 | 3 | 134.52 | 25 | 0.357 | 284.70 | 7.118 | 1.058 |
| 12 | 3 | 112.70 | 25 | 0.413 | 212.30 | 5.307 | 0.942 |
| 13 | 4 | 110.37 | 25 | 0.382 | 248.03 | 6.201 | 1.124 |
| 14 | 4 | 143.10 | 25 | 0.383 | 246.73 | 6.168 | 0.862 |
| 15 | 4 | 136.66 | 25 | 0.329 | 338.35 | 8.459 | 1.238 |
| 16 | 4 | 128.44 | 25 | 0.342 | 311.42 | 7.786 | 1.212 |
