# Supplementary material for: Effects of shinbuto and ninjinto on prostaglandin E2 production in lipopolysaccharide-treated human gingival fibroblasts
Source: PeerJ. 2017 Dec 1;5:e4120. doi: 10.7717/peerj.4120 (PMC5713626; doi:10.7717/peerj.4120)
Supplement: Data S1 [file peerj-05-4120-s001.zip › Fig6/025_shokyo_PGE2-2.pdf]

- Exp. 25
- Condition
  - drug1: shokyo (ug/ml)
  - experimental No. 2
  - treatment: 24h
- Measurement
  - PGE2
  - Date: 2017.4.11
- Cells
  - cells: HGFs (No. 1), passages: 13
  - cell numbers: 0.8×10^4 cells/well = 4×10^4 cells/ml

	conc.	OD
1	7.8	1.061
2	15.6	0.911
3	31.2	0.755
4	62.5	0.624
5	125.0	0.408
6	250.0	0.280
7	500.0	0.239
8	1000.0	0.205

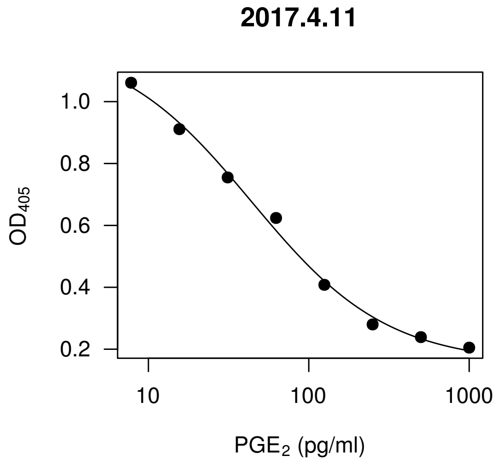

	drug1	mean	SD
1	0	0.414	0.093
2	1	0.301	0.032
3	10	0.212	0.046
4	100	0.068	0.042

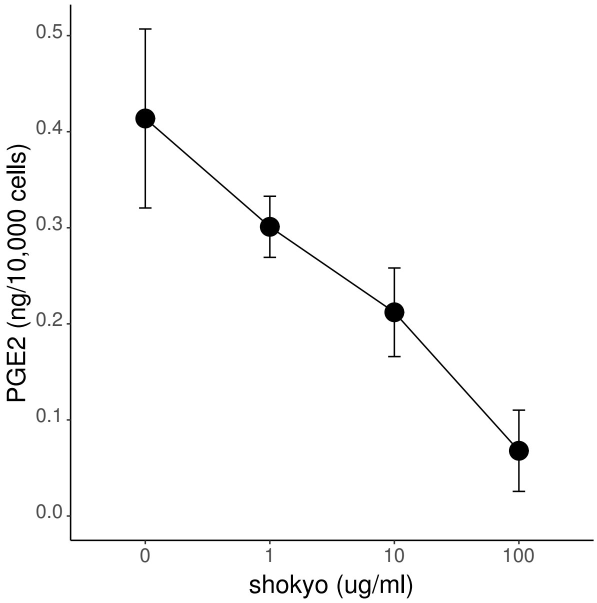

	drug1	viability	dilution	OD	conc. (pg/ml)	net (ng/ml)	(ng/10,000 cells)
1	0	96.29	25	0.526	78.37	1.959	0.509
2	0	102.23	25	0.628	52.75	1.319	0.322
3	0	101.47	25	0.567	66.58	1.664	0.410
4	1	101.32	25	0.624	53.54	1.339	0.330
5	1	113.20	25	0.651	48.39	1.210	0.267
6	1	100.86	25	0.646	49.30	1.233	0.306
7	10	102.39	25	0.680	43.43	1.086	0.265
8	10	100.71	25	0.784	29.30	0.733	0.182
9	10	97.97	25	0.781	29.65	0.741	0.189
10	100	114.57	25	1.047	7.83	0.196	0.043
11	100	109.69	25	1.048	7.77	0.194	0.044
12	100	102.54	25	0.887	19.15	0.479	0.117
