# Supplementary material for: Effects of shinbuto and ninjinto on prostaglandin E2 production in lipopolysaccharide-treated human gingival fibroblasts
Source: PeerJ. 2017 Dec 1;5:e4120. doi: 10.7717/peerj.4120 (PMC5713626; doi:10.7717/peerj.4120)
Supplement: Data S1 [file peerj-05-4120-s001.zip › Fig6/025_herb_PGE2-2.pdf]

- Exp. 25
- Condition
  - drug1: herb ()
  - experimental No. 2
  - treatment: 24h
- Measurement
  - PGE2
  - Date: 2017.1.28
- Cells
  - cells: HGFs (No. 1), passages: 13
  - cell numbers: 0.667×10^4 cells/well = 3.335×10^4 cells/ml

	conc.	OD
1	7.8	0.553
2	15.6	0.455
3	31.2	0.370
4	62.5	0.307
5	125.0	0.270
6	250.0	0.229
7	500.0	0.210
8	1000.0	0.197

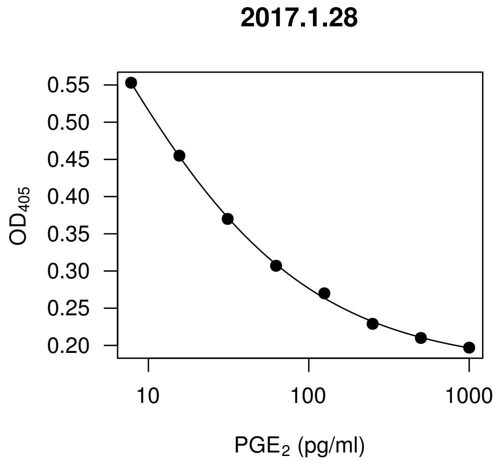

	drug1	mean	SD
1	1	0.173	0.043
2	2	1.887	0.507
3	3	2.806	0.257
4	4	1.367	0.159
5	5	1.979	0.384
6	6	0.555	0.047
7	7	1.808	0.330
8	8	0.322	0.024
9	9	0.889	0.111
10	10	2.123	0.401

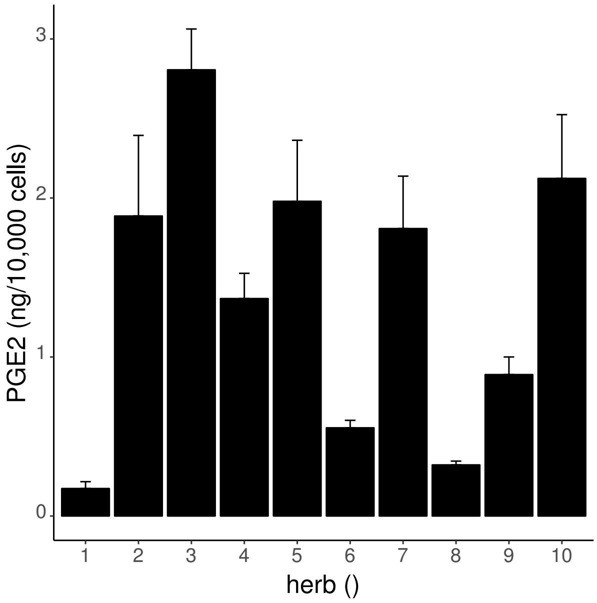

	drug1	viability	dilution	OD	conc. (pg/ml)	net (ng/ml)	(ng/10,000 cells)
1	1	95.83	50	0.468	14.04	0.702	0.220
2	1	105.74	50	0.523	9.52	0.476	0.135
3	1	98.43	50	0.505	10.77	0.539	0.164
4	2	109.15	50	0.246	177.90	8.895	2.444
5	2	111.27	50	0.272	107.80	5.390	1.452
6	2	111.43	50	0.261	131.12	6.556	1.764
7	3	105.25	50	0.243	190.36	9.518	2.712
8	3	106.88	50	0.244	186.06	9.303	2.610
9	3	103.79	50	0.238	214.37	10.719	3.097
10	4	110.94	50	0.274	104.24	5.212	1.409
11	4	111.75	50	0.284	88.86	4.443	1.192
12	4	109.48	50	0.271	109.64	5.482	1.501
13	5	107.85	50	0.247	174.03	8.702	2.419
14	5	108.99	50	0.261	131.12	6.556	1.804
15	5	110.45	50	0.263	126.33	6.317	1.715
16	6	81.69	50	0.369	32.13	1.606	0.590
17	6	102.82	50	0.349	39.31	1.965	0.573
18	6	103.95	50	0.361	34.76	1.738	0.501
19	7	110.29	50	0.252	156.50	7.825	2.127
20	7	111.75	50	0.259	136.18	6.809	1.827
21	7	111.92	50	0.271	109.64	5.482	1.469
22	8	110.29	50	0.394	25.48	1.274	0.346
23	8	111.27	50	0.410	22.18	1.109	0.299
24	8	108.67	50	0.405	23.15	1.157	0.319
25	9	113.70	50	0.294	76.66	3.833	1.011
26	9	113.70	50	0.312	60.19	3.010	0.794
27	9	112.89	50	0.306	65.05	3.252	0.864
28	10	108.02	50	0.244	186.06	9.303	2.582
29	10	104.77	50	0.262	128.69	6.435	1.842
30	10	111.27	50	0.256	144.36	7.218	1.945
