# Supplementary material for: Effects of shinbuto and ninjinto on prostaglandin E2 production in lipopolysaccharide-treated human gingival fibroblasts
Source: PeerJ. 2017 Dec 1;5:e4120. doi: 10.7717/peerj.4120 (PMC5713626; doi:10.7717/peerj.4120)
Supplement: Data S1 [file peerj-05-4120-s001.zip › Fig6/025_kankyo_PGE2-2.pdf]

- Exp. 25
- Condition
  - drug1: kankyo (ug/ml)
  - experimental No. 2
  - treatment: 24h
- Measurement
  - PGE2
  - Date: 2017.4.11
- Cells
  - cells: HGFs (No. 1), passages: 13
  - cell numbers: 0.8×10^4 cells/well = 4×10^4 cells/ml

	conc.	OD
1	7.8	1.061
2	15.6	0.911
3	31.2	0.755
4	62.5	0.624
5	125.0	0.408
6	250.0	0.280
7	500.0	0.239
8	1000.0	0.205

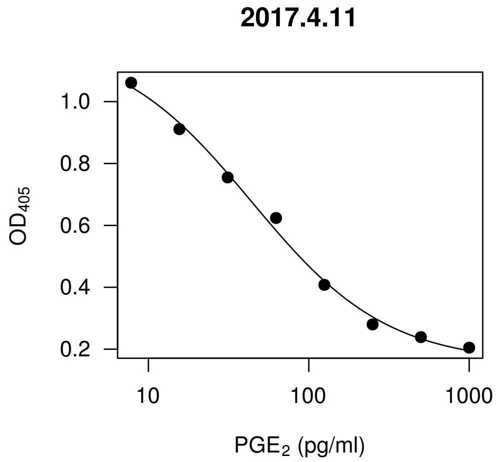

	drug1	mean	SD
1	0	0.463	0.094
2	1	0.351	0.109
3	10	0.170	0.084
4	100	0.029	0.015

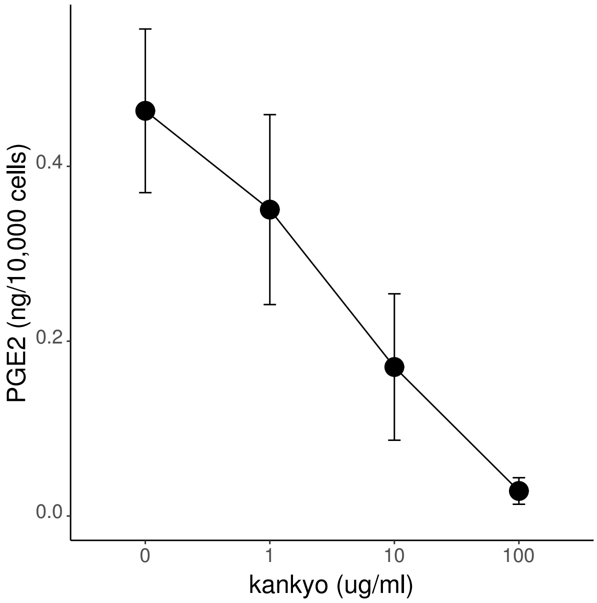

	drug1	viability	dilution	OD	conc. (pg/ml)	net (ng/ml)	(ng/10,000 cells)
1	0	105.22	25	0.568	66.32	1.658	0.394
2	0	106.36	25	0.545	72.60	1.815	0.427
3	0	88.43	25	0.519	80.64	2.016	0.570
4	1	105.79	25	0.611	56.24	1.406	0.332
5	1	100.66	25	0.698	40.61	1.015	0.252
6	1	87.71	25	0.571	65.55	1.639	0.467
7	10	99.81	25	0.685	42.63	1.066	0.267
8	10	101.94	25	0.868	20.81	0.520	0.128
9	10	90.13	25	0.915	16.85	0.421	0.117
10	100	106.07	25	1.106	4.54	0.114	0.027
11	100	94.97	25	1.065	6.79	0.170	0.045
12	100	95.54	25	1.152	2.21	0.055	0.014
