# Supplementary figures and images for: Effects of shinbuto and ninjinto on prostaglandin E2 production in lipopolysaccharide-treated human gingival fibroblasts
Source: PeerJ. 2017 Dec 1;5:e4120. doi: 10.7717/peerj.4120 (PMC5713626; doi:10.7717/peerj.4120)

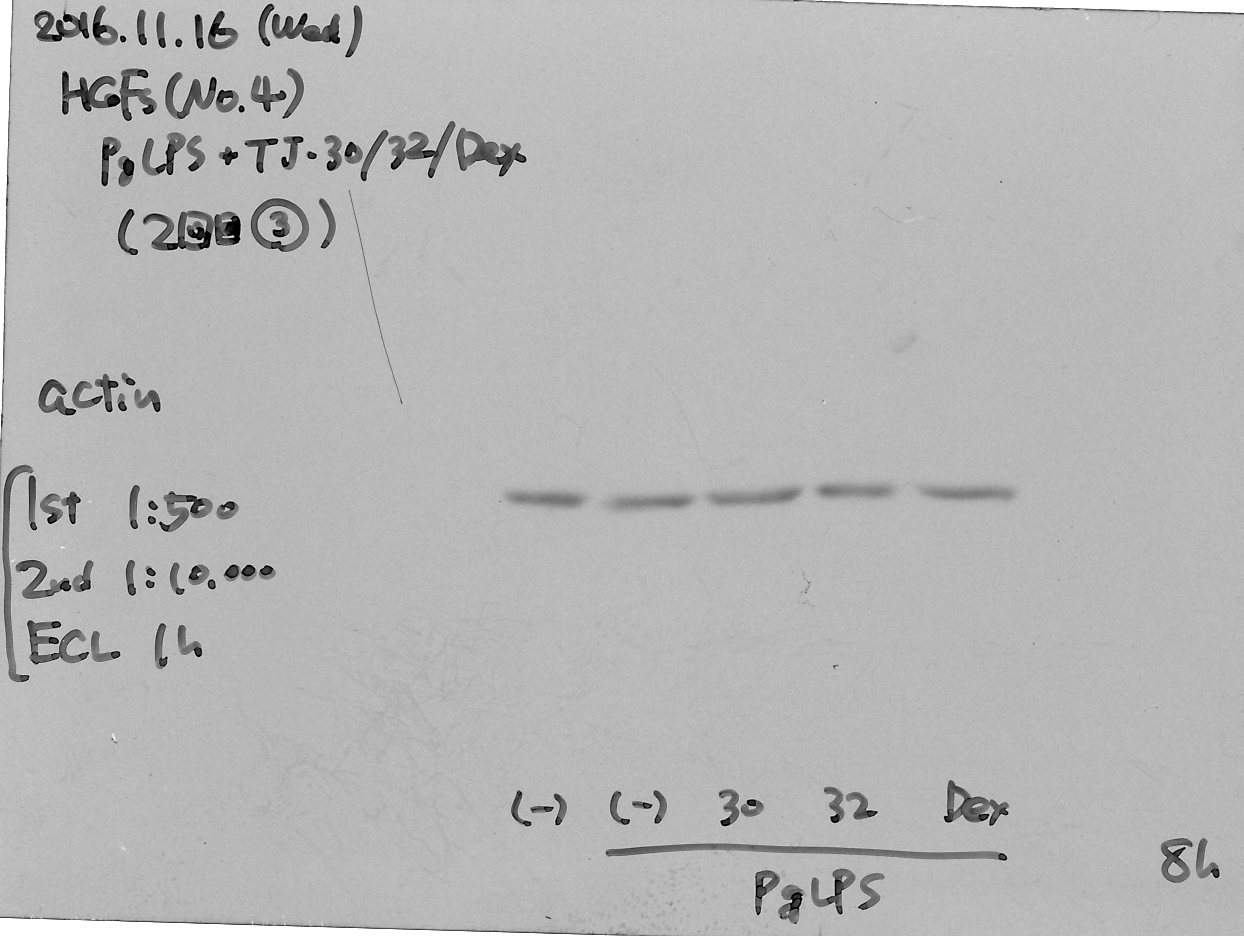

Supplement: Data S2 [file peerj-05-4120-s002.zip › Fig4A/actin-20161116-01.tif]

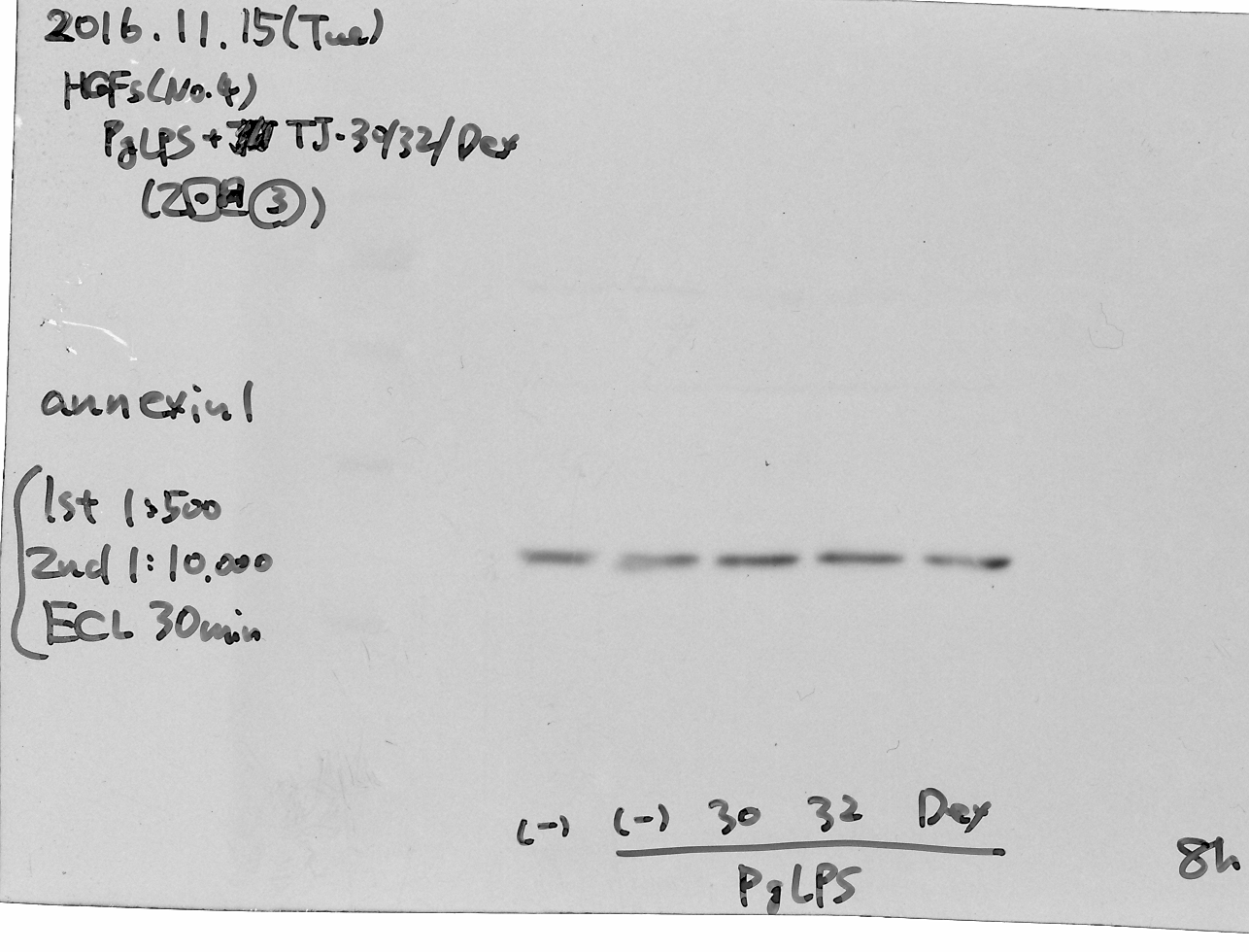

Supplement: Data S2 [file peerj-05-4120-s002.zip › Fig4A/annexin1-20161115-01.tif]

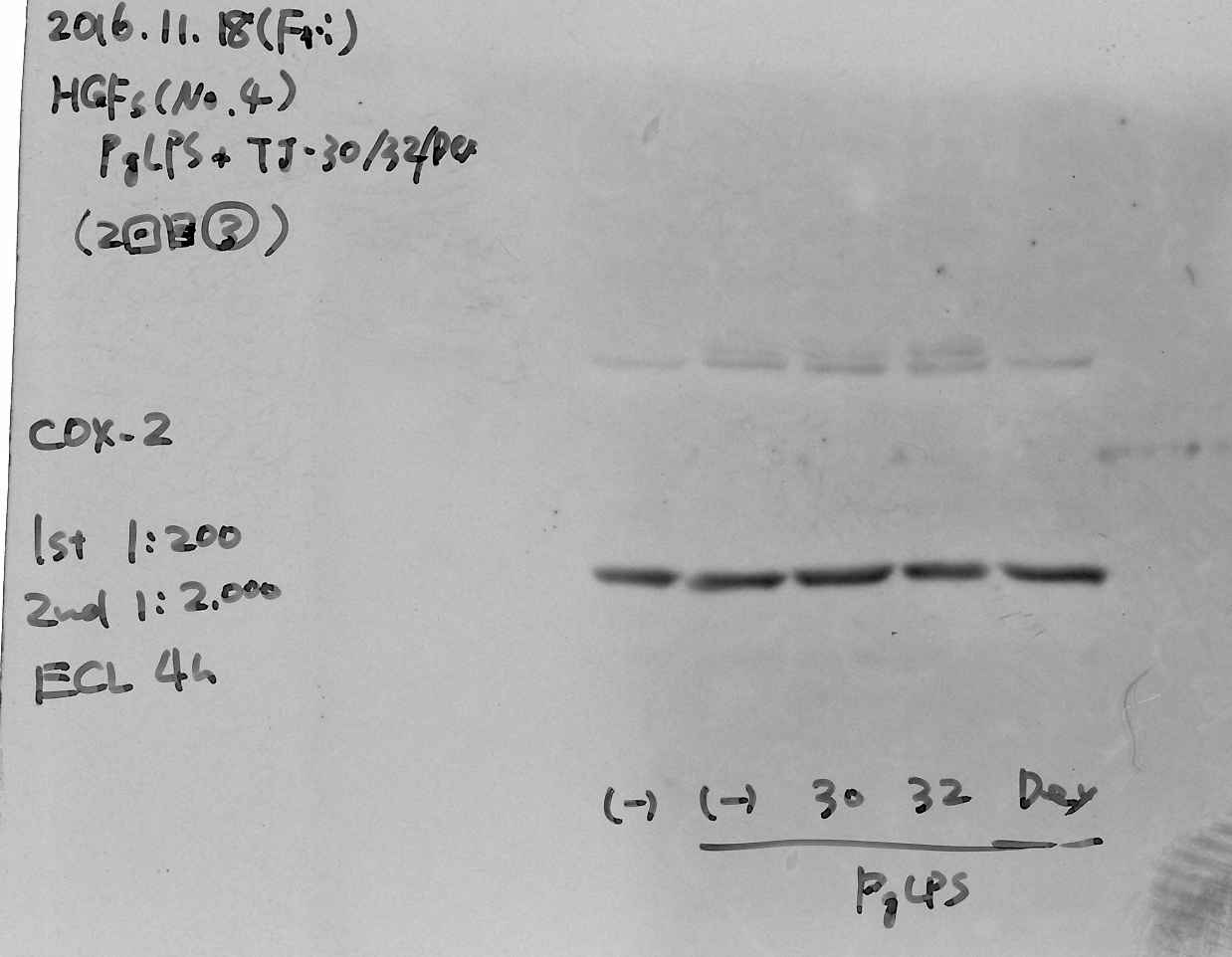

Supplement: Data S2 [file peerj-05-4120-s002.zip › Fig4A/COX2-20161118-01.tif]

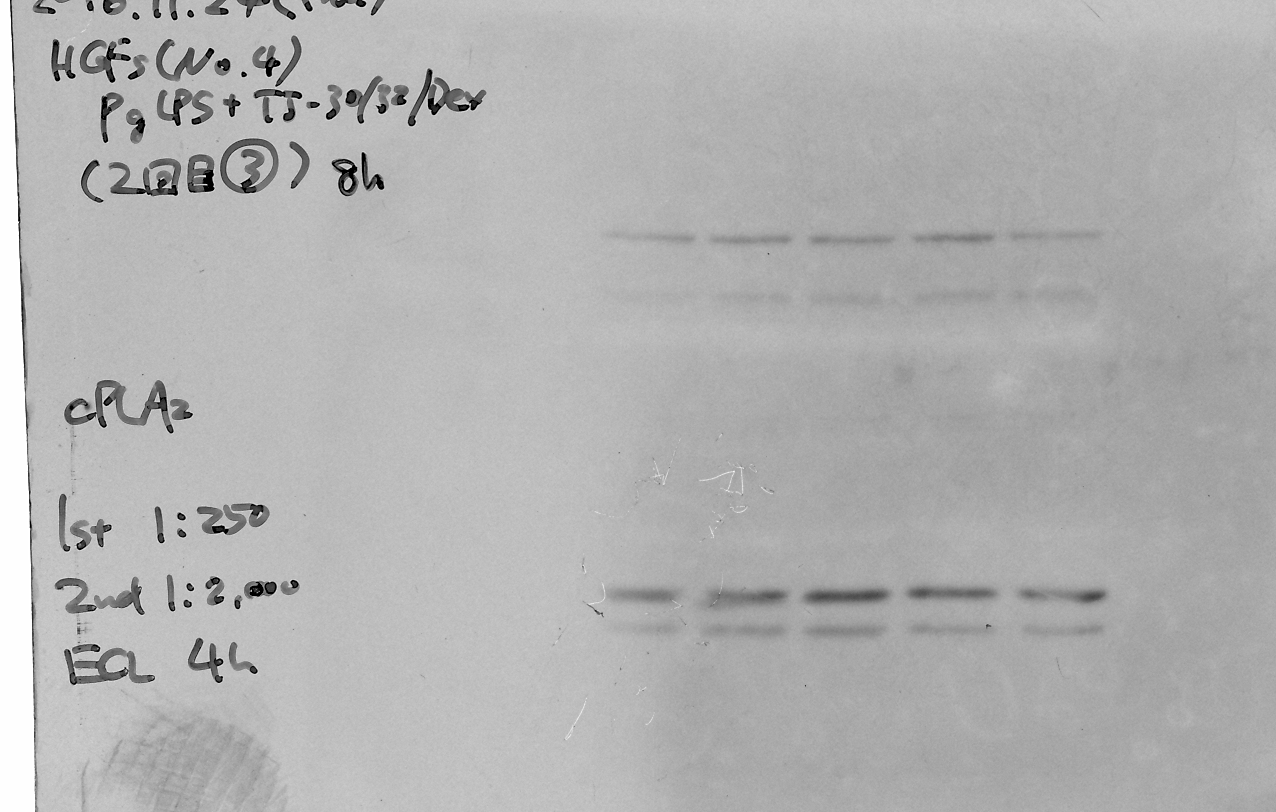

Supplement: Data S2 [file peerj-05-4120-s002.zip › Fig4A/cPLA2-20161124-01.tif]

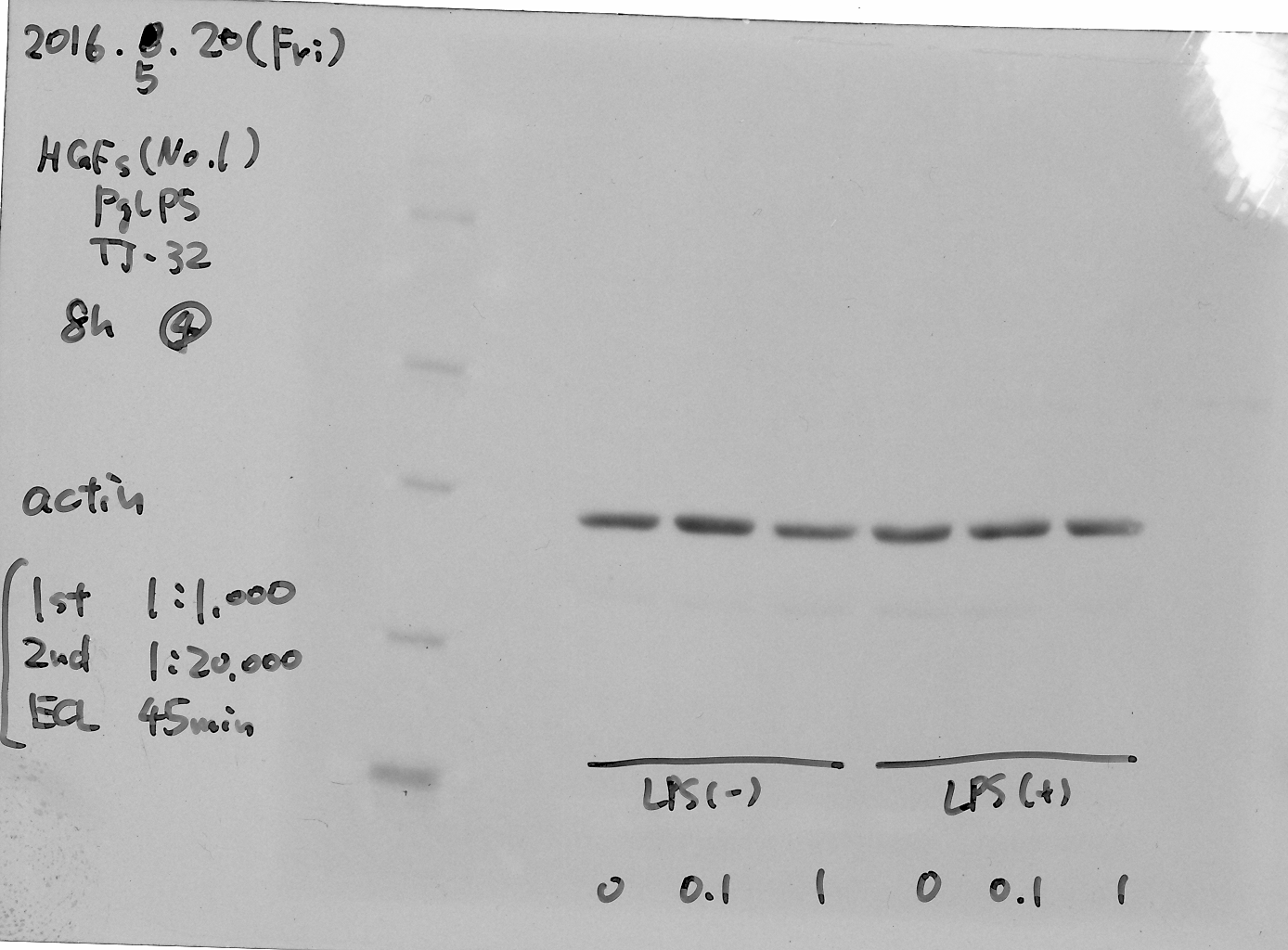

Supplement: Data S2 [file peerj-05-4120-s002.zip › Fig4B/actin-20160520-01.tif]

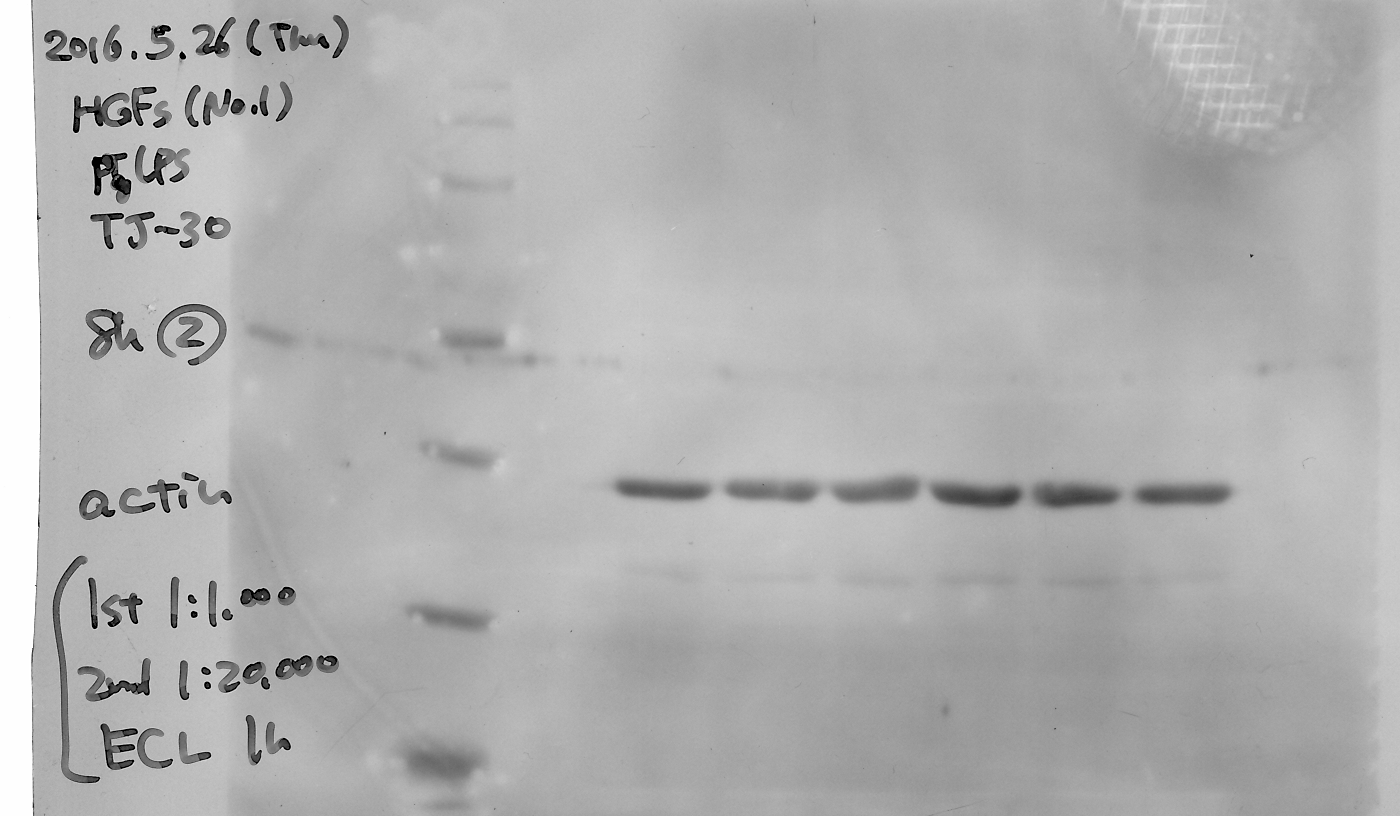

Supplement: Data S2 [file peerj-05-4120-s002.zip › Fig4B/actin-20160526-01.tif]

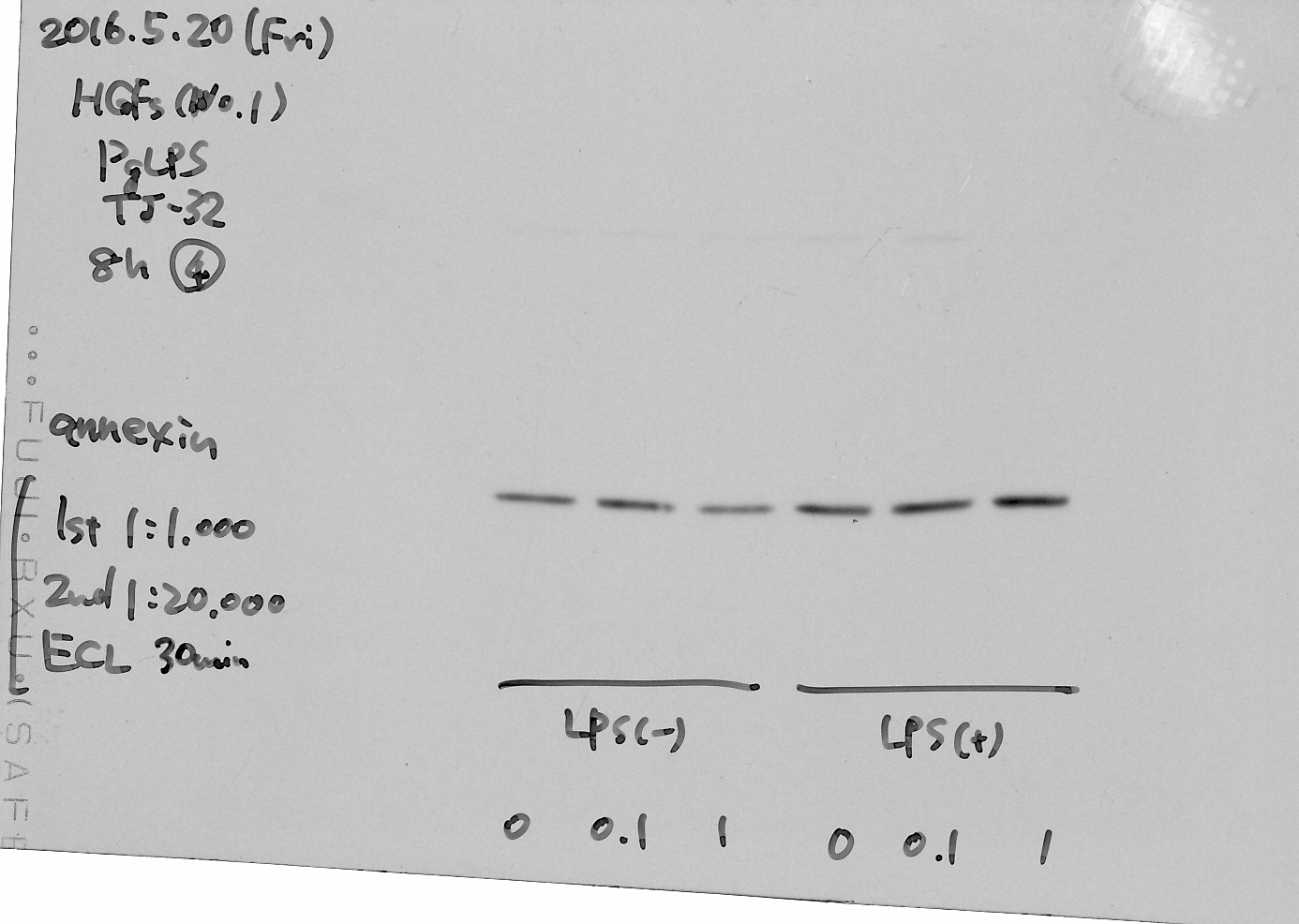

Supplement: Data S2 [file peerj-05-4120-s002.zip › Fig4B/annexin1-20160520-01.tif]

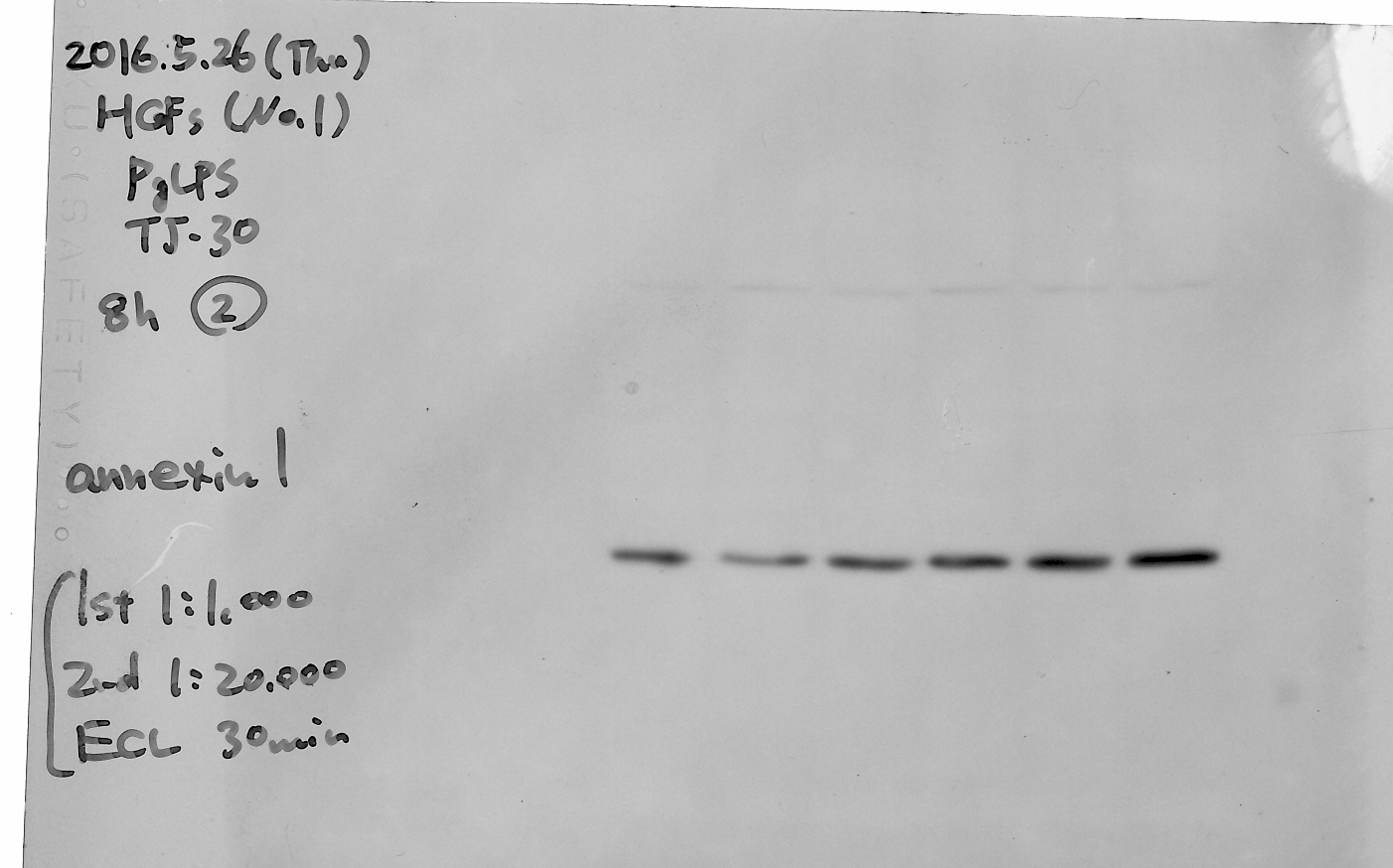

Supplement: Data S2 [file peerj-05-4120-s002.zip › Fig4B/annexin1-20160526-01.tif]

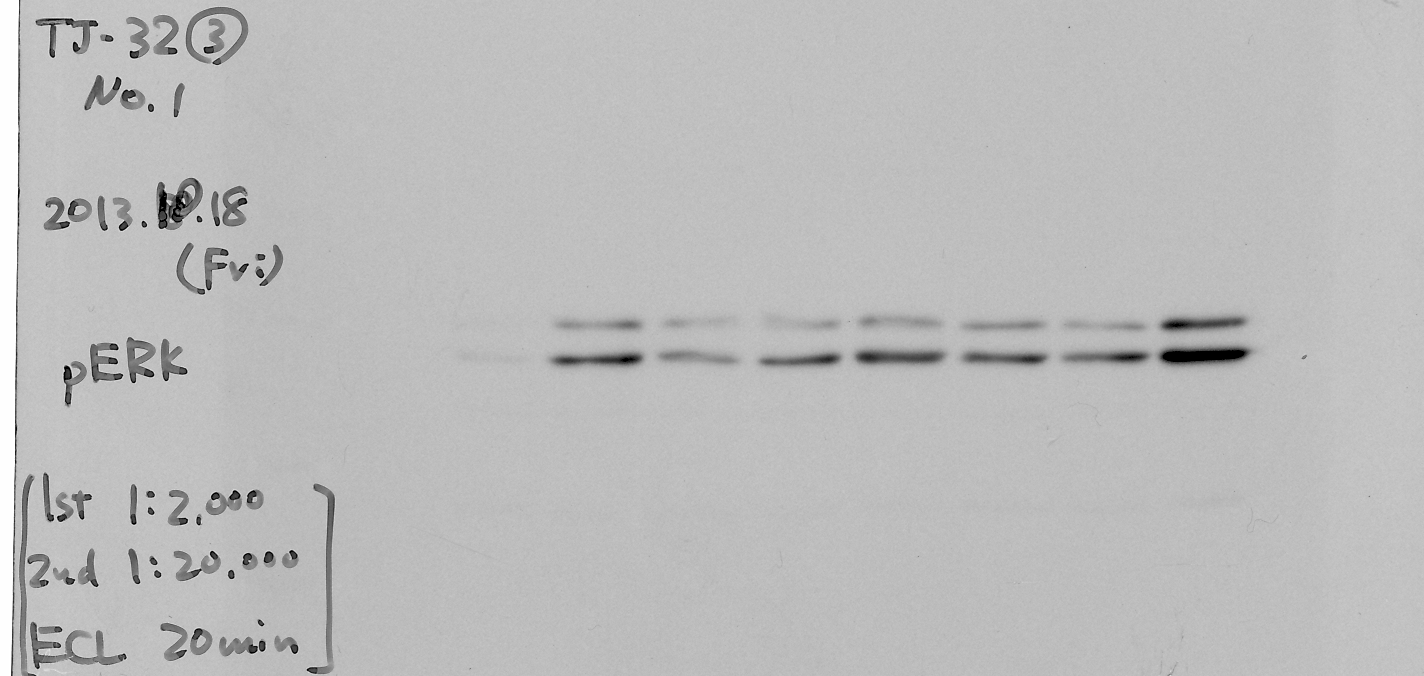

Supplement: Data S2 [file peerj-05-4120-s002.zip › Fig5/TJ32_pERK-20131018-01.tif]

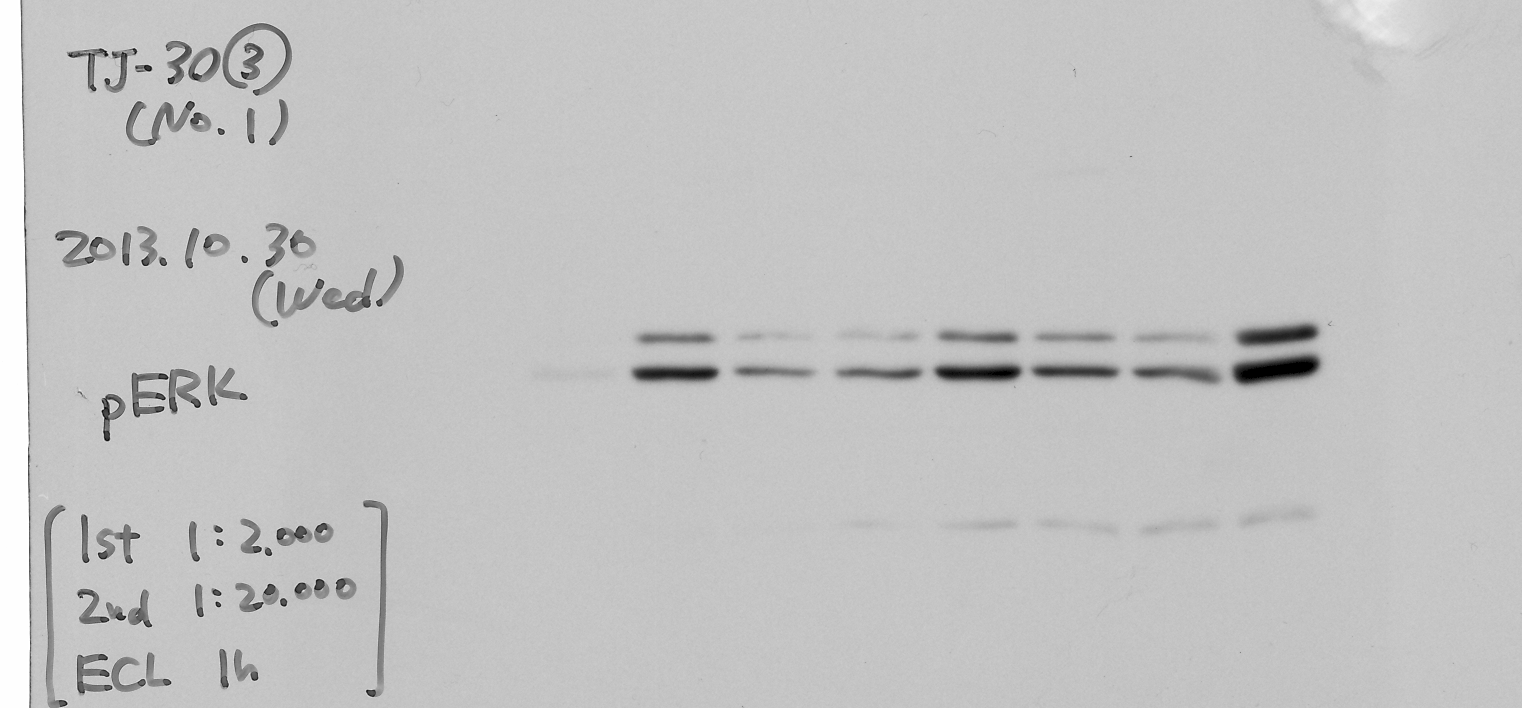

Supplement: Data S2 [file peerj-05-4120-s002.zip › Fig5/TJ30_pERK-20131030-01.tif]

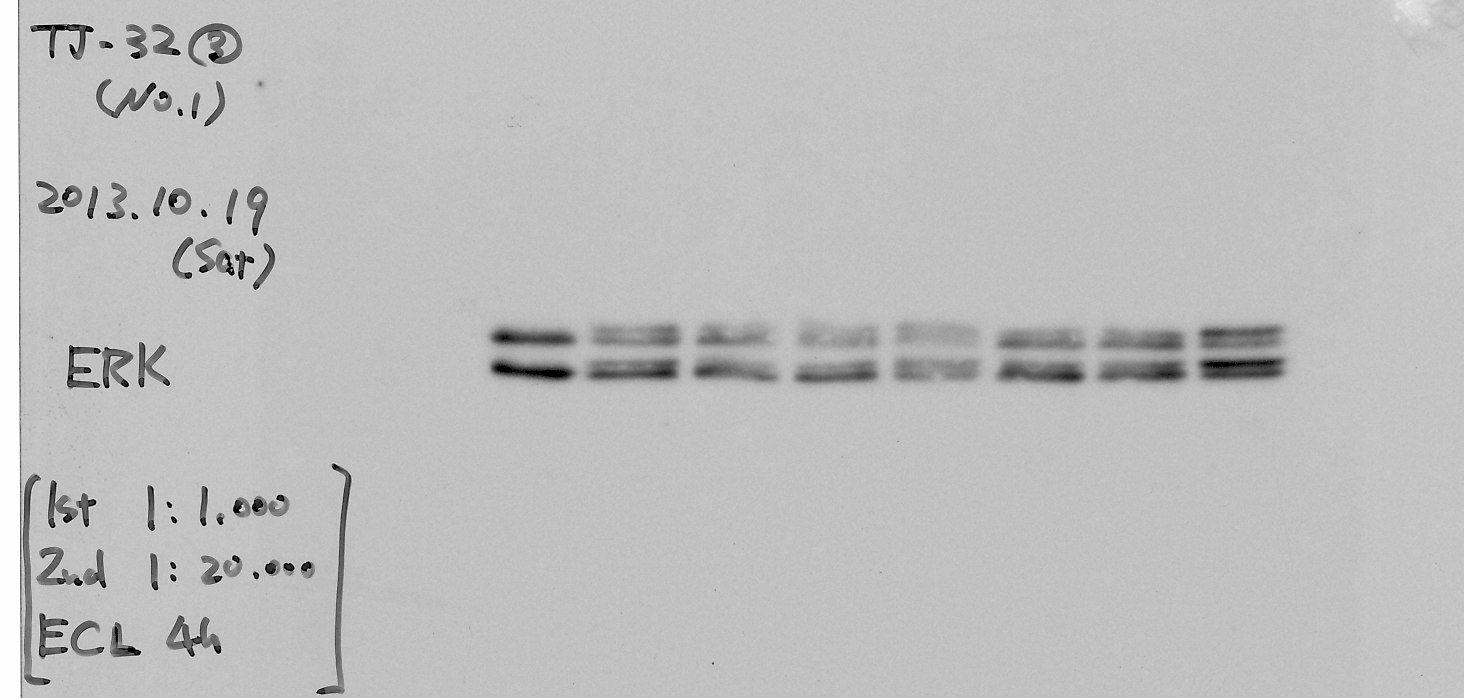

Supplement: Data S2 [file peerj-05-4120-s002.zip › Fig5/TJ32_ERK-20131019-01.tif]

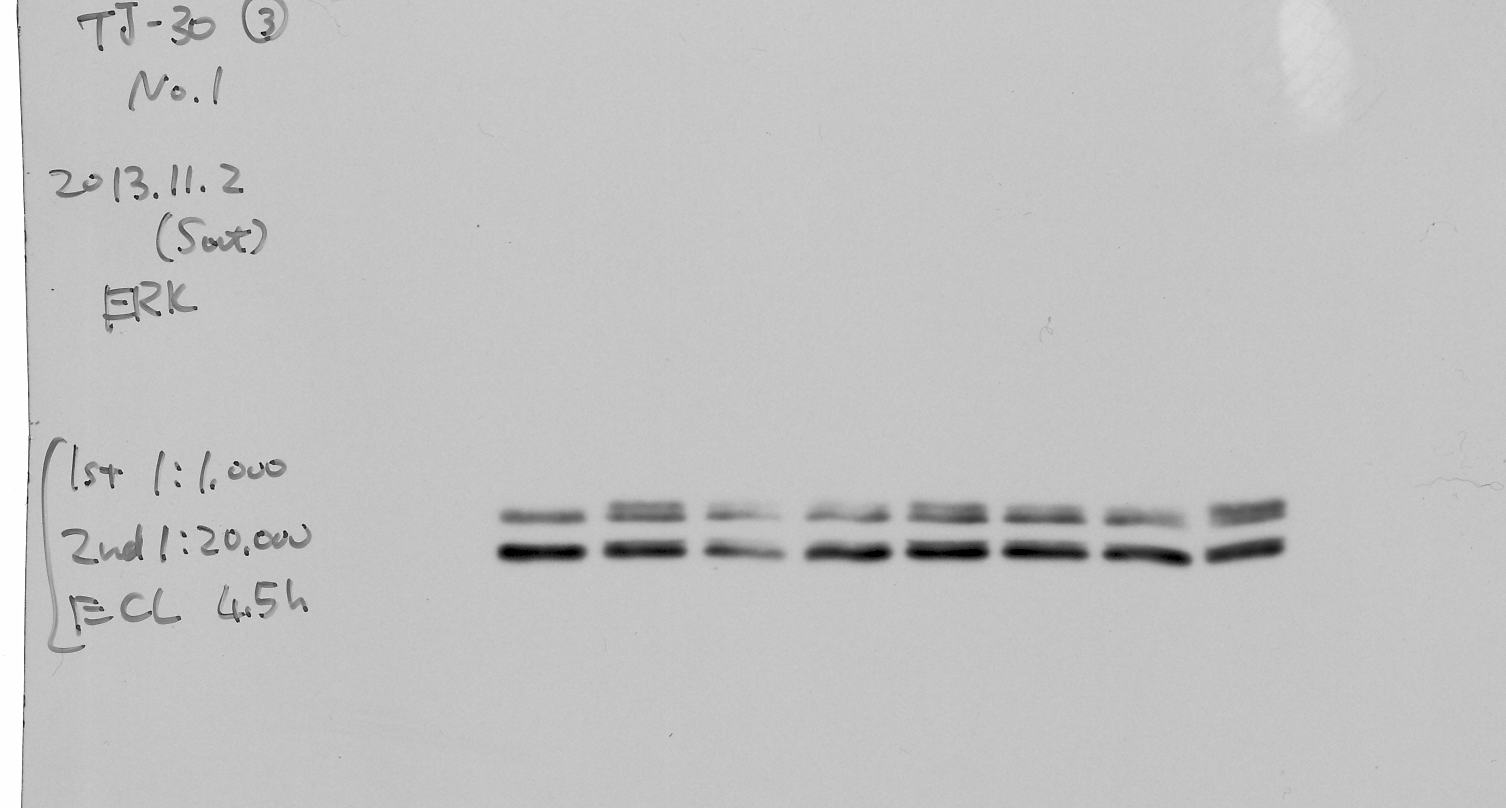

Supplement: Data S2 [file peerj-05-4120-s002.zip › Fig5/TJ30_ERK-20131102-01.tif]

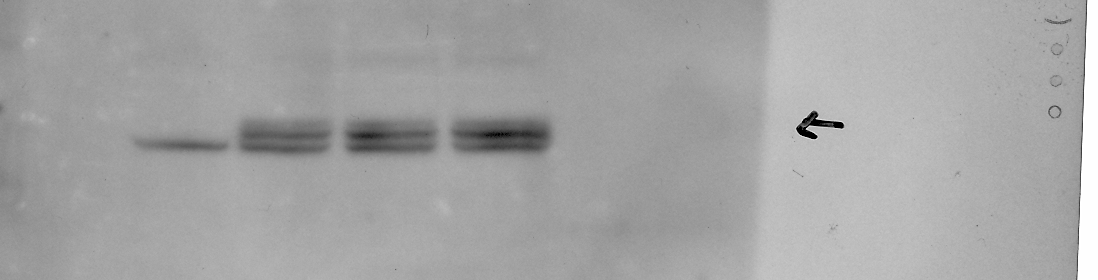

Supplement: Data S2 [file peerj-05-4120-s002.zip › Fig7/COX2-20170329-01.tif]

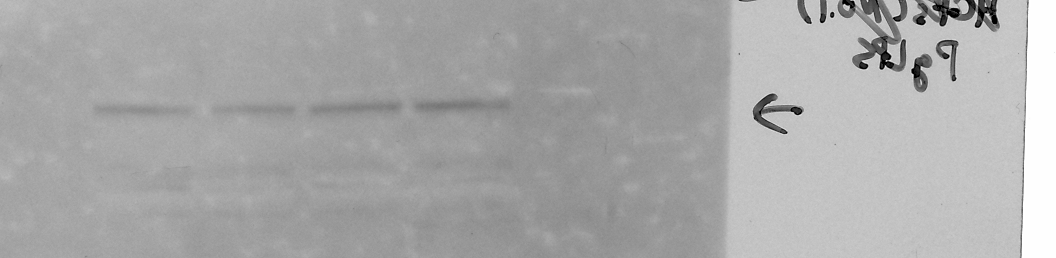

Supplement: Data S2 [file peerj-05-4120-s002.zip › Fig7/cPLA2-20170331-01.tif]

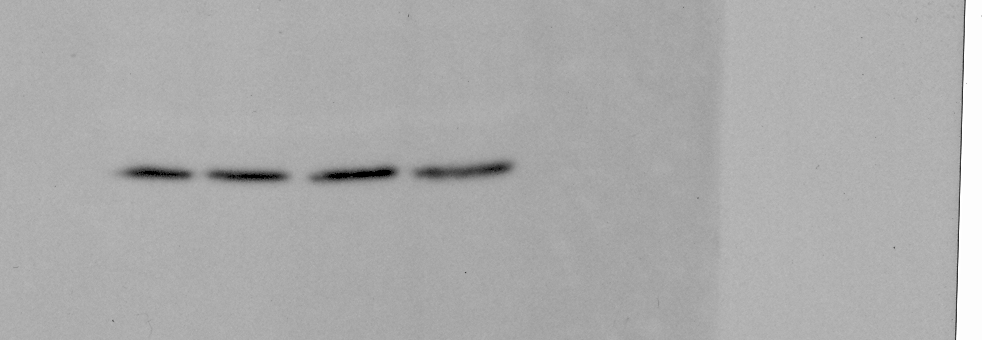

Supplement: Data S2 [file peerj-05-4120-s002.zip › Fig7/annexin1-20170330-01.tif]

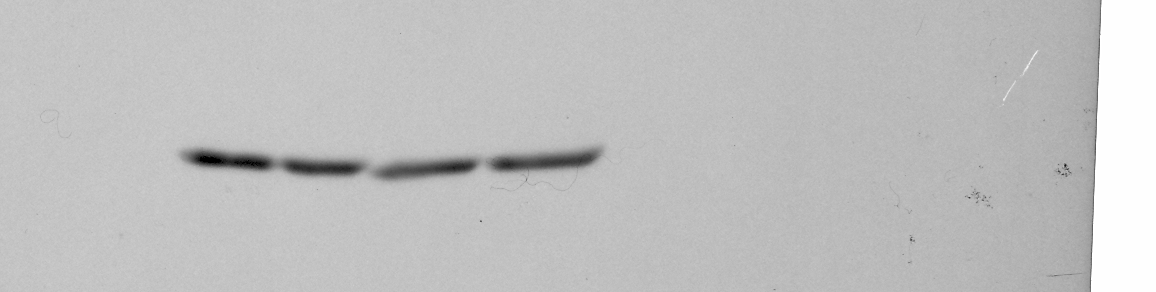

Supplement: Data S2 [file peerj-05-4120-s002.zip › Fig7/actin-20170328-01.tif]

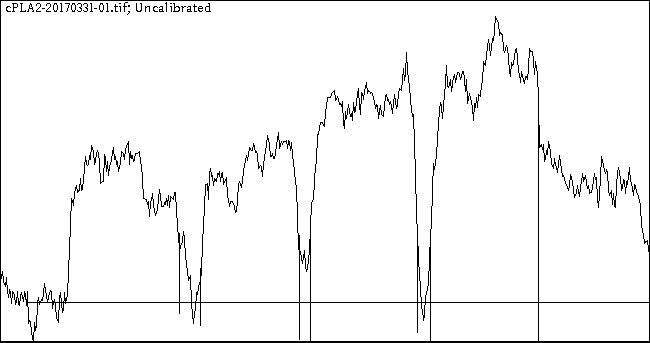

Supplement: Data S3 [file peerj-05-4120-s003.zip › revise_data/Western-analyzed/Fig7/cPLA2.tif]

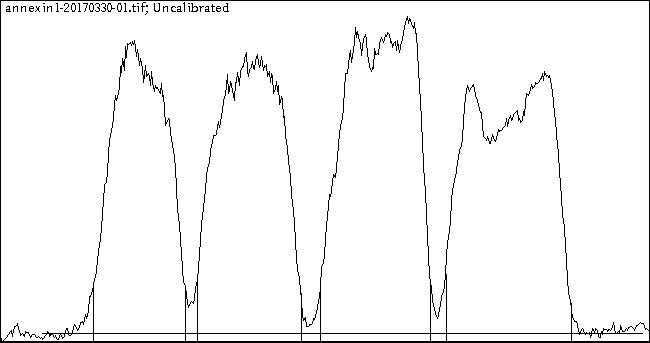

Supplement: Data S3 [file peerj-05-4120-s003.zip › revise_data/Western-analyzed/Fig7/annexin1.tif]

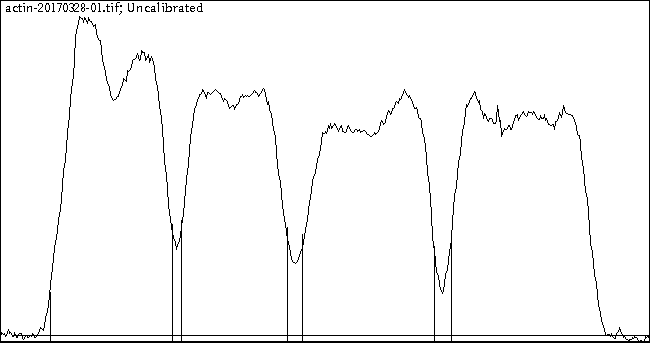

Supplement: Data S3 [file peerj-05-4120-s003.zip › revise_data/Western-analyzed/Fig7/actin.tif]

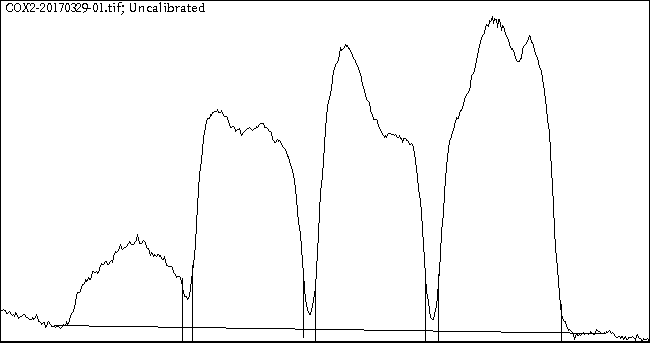

Supplement: Data S3 [file peerj-05-4120-s003.zip › revise_data/Western-analyzed/Fig7/COX2.tif]

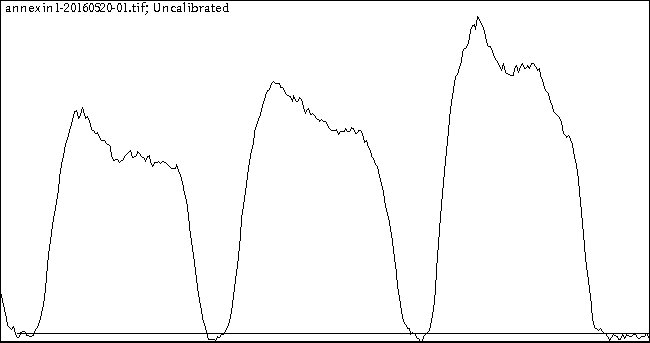

Supplement: Data S3 [file peerj-05-4120-s003.zip › revise_data/Western-analyzed/Fig4B/annexin32.tif]

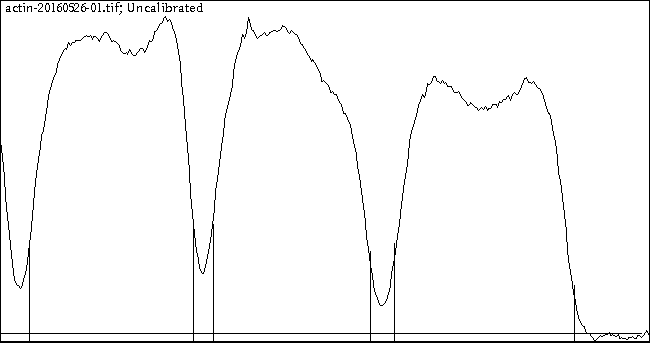

Supplement: Data S3 [file peerj-05-4120-s003.zip › revise_data/Western-analyzed/Fig4B/actin32.tif]

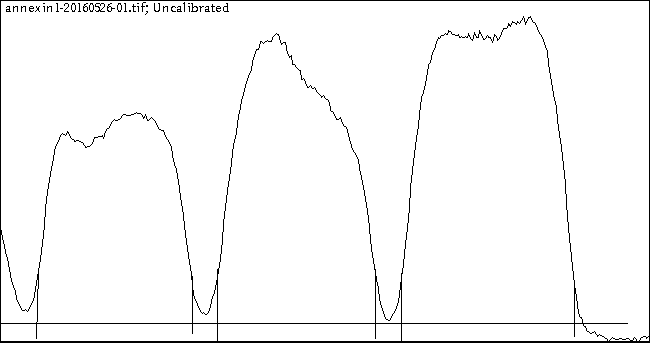

Supplement: Data S3 [file peerj-05-4120-s003.zip › revise_data/Western-analyzed/Fig4B/annexin30.tif]

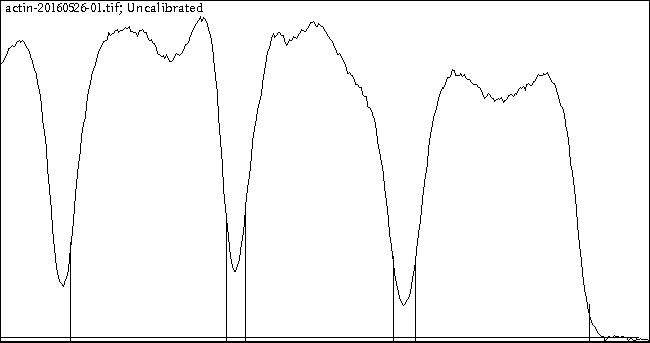

Supplement: Data S3 [file peerj-05-4120-s003.zip › revise_data/Western-analyzed/Fig4B/actin30.tif]

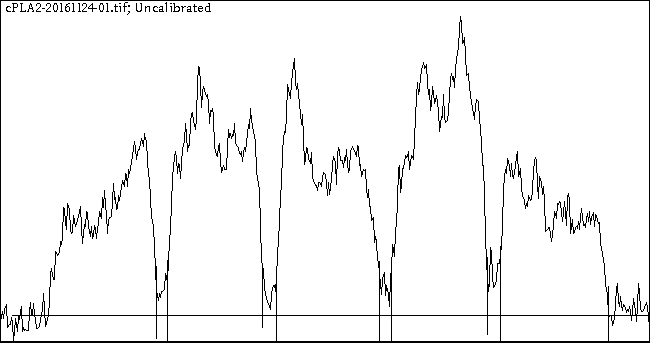

Supplement: Data S3 [file peerj-05-4120-s003.zip › revise_data/Western-analyzed/Fig4A/cPLA2.tif]

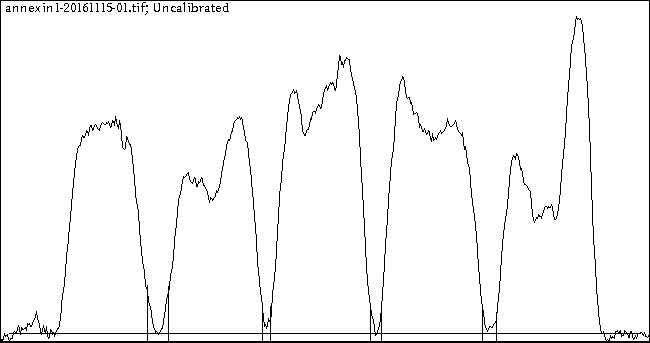

Supplement: Data S3 [file peerj-05-4120-s003.zip › revise_data/Western-analyzed/Fig4A/annexin1.tif]

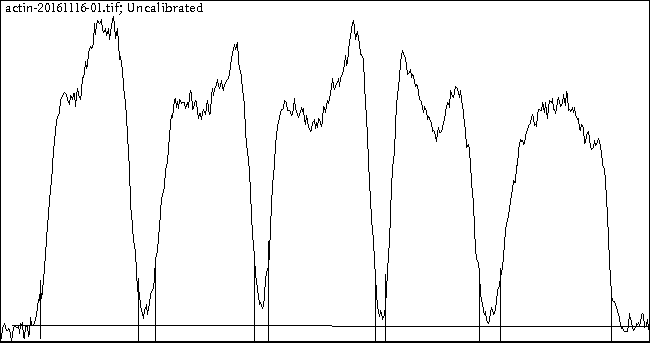

Supplement: Data S3 [file peerj-05-4120-s003.zip › revise_data/Western-analyzed/Fig4A/actin.tif]

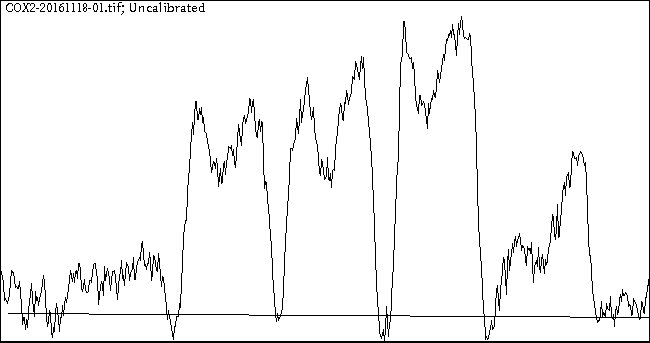

Supplement: Data S3 [file peerj-05-4120-s003.zip › revise_data/Western-analyzed/Fig4A/COX2.tif]
