# Supplementary material for: Effects of shinbuto and ninjinto on prostaglandin E2 production in lipopolysaccharide-treated human gingival fibroblasts
Source: PeerJ. 2017 Dec 1;5:e4120. doi: 10.7717/peerj.4120 (PMC5713626; doi:10.7717/peerj.4120)
Supplement: Data S3 [file peerj-05-4120-s003.zip › revise_data/025_herb_IL6-2-2.pdf]

- Exp. 25
- Condition
  - drug1: herb ()
  - experimental No. 2
  - treatment: 24h
- Measurement
  - IL-6
  - Date: 2017.10.23
- Cells
  - cells: HGFs (No. 1), passages: 13
  - cell numbers: 0.667×10^4 cells/well = 3.335×10^4 cells/ml

	conc.	OD	OD-blank
1	0.0	0.077	0.000
2	31.2	0.095	0.018
3	62.5	0.103	0.026
4	125.0	0.144	0.067
5	250.0	0.209	0.132
6	500.0	0.347	0.270
7	1000.0	0.588	0.511

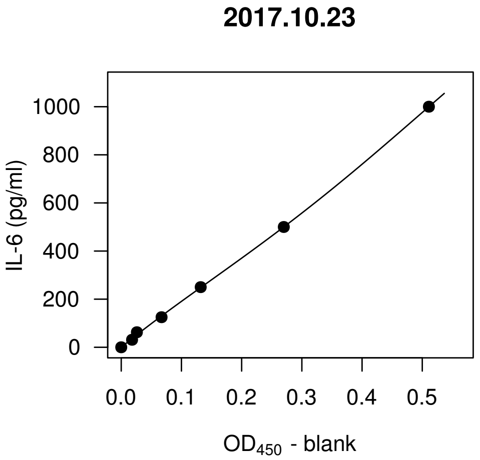

	drug1	mean	SD
1	1	0.037	0.023
2	2	2.701	0.112
3	3	4.688	0.738
4	4	2.461	0.133
5	5	2.661	0.187
6	6	2.134	0.180
7	7	2.401	0.070
8	8	2.676	0.329
9	9	1.956	0.151
10	10	2.528	0.142

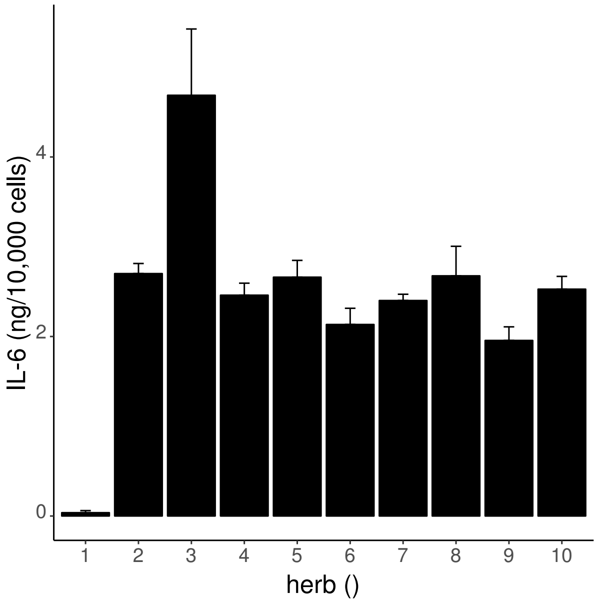

	drug1	viability	dilution	OD	conc. (pg/ml)	net (ng/ml)	(ng/10,000 cells)
1	1	95.83	20	0.082	10.22	0.204	0.064
2	1	105.74	20	0.079	4.10	0.082	0.023
3	1	98.43	20	0.079	4.10	0.082	0.025
4	2	109.15	20	0.331	469.96	9.399	2.582
5	2	111.27	20	0.349	503.98	10.080	2.716
6	2	111.43	20	0.358	521.17	10.423	2.805
7	3	105.25	20	0.560	939.11	18.782	5.351
8	3	106.88	20	0.523	858.95	17.179	4.820
9	3	103.79	20	0.435	673.68	13.474	3.893
10	4	110.94	20	0.328	464.33	9.287	2.510
11	4	111.75	20	0.335	477.47	9.549	2.562
12	4	109.48	20	0.305	421.62	8.432	2.309
13	5	107.85	20	0.355	515.42	10.308	2.866
14	5	108.99	20	0.334	475.59	9.512	2.617
15	5	110.45	20	0.326	460.59	9.212	2.501
16	6	81.69	20	0.228	282.34	5.647	2.073
17	6	102.82	20	0.261	341.54	6.831	1.992
18	6	103.95	20	0.296	405.08	8.102	2.337
19	7	110.29	20	0.314	438.25	8.765	2.383
20	7	111.75	20	0.313	436.40	8.728	2.342
21	7	111.92	20	0.327	462.46	9.249	2.478
22	8	110.29	20	0.379	561.79	11.236	3.055
23	8	111.27	20	0.324	456.85	9.137	2.462
24	8	108.67	20	0.323	454.99	9.100	2.511
25	9	113.70	20	0.276	368.64	7.373	1.944
26	9	113.70	20	0.262	343.34	6.867	1.811
27	9	112.89	20	0.292	397.76	7.955	2.113
28	10	108.02	20	0.324	456.85	9.137	2.536
29	10	104.77	20	0.302	416.09	8.322	2.382
30	10	111.27	20	0.344	494.48	9.890	2.665
