# Supplementary material for: Effects of shinbuto and ninjinto on prostaglandin E2 production in lipopolysaccharide-treated human gingival fibroblasts
Source: PeerJ. 2017 Dec 1;5:e4120. doi: 10.7717/peerj.4120 (PMC5713626; doi:10.7717/peerj.4120)
Supplement: Data S3 [file peerj-05-4120-s003.zip › revise_data/025_herb_IL8-2.pdf]

- Exp. 25
- Condition
  - drug1: herb ()
  - experimental No. 2
  - treatment: 24h
- Measurement
  - IL-8
  - Date: 2017.10.20
- Cells
  - cells: HGFs (No. 1), passages: 13
  - cell numbers: 0.667×10^4 cells/well = 3.335×10^4 cells/ml

	conc.	OD	OD-blank
1	0.0	0.185	0.000
2	15.6	0.207	0.022
3	31.2	0.222	0.037
4	62.5	0.265	0.080
5	125.0	0.345	0.160
6	250.0	0.478	0.293
7	500.0	0.620	0.435
8	1000.0	0.782	0.597

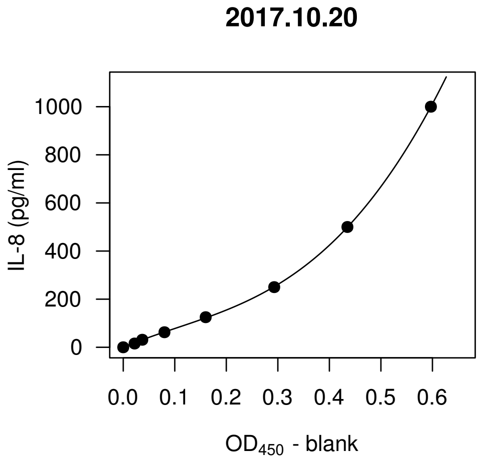

	drug1	mean	SD
1	1	0.041	0.037
2	2	3.587	0.304
3	3	6.652	0.855
4	4	2.665	0.053
5	5	3.497	0.232
6	6	4.526	0.604
7	7	3.099	0.224
8	8	5.322	0.792
9	9	3.217	0.097
10	10	3.549	0.167

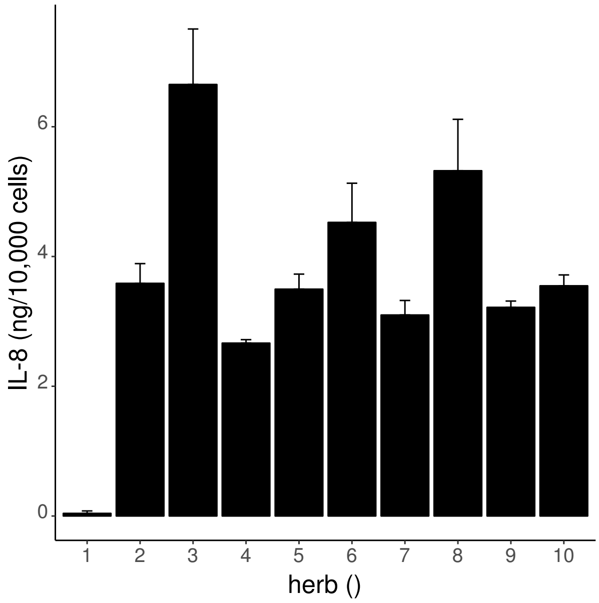

	drug1	viability	dilution	OD	conc. (pg/ml)	net (ng/ml)	(ng/10,000 cells)
1	1	95.83	50	0.191	5.32	0.266	0.083
2	1	105.74	50	0.186	0.89	0.045	0.013
3	1	98.43	50	0.187	1.79	0.089	0.027
4	2	109.15	50	0.476	249.51	12.476	3.427
5	2	111.27	50	0.478	252.03	12.602	3.396
6	2	111.43	50	0.508	292.61	14.630	3.937
7	3	105.25	50	0.606	467.96	23.398	6.666
8	3	106.88	50	0.635	534.61	26.731	7.499
9	3	103.79	50	0.573	400.80	20.040	5.790
10	4	110.94	50	0.426	193.07	9.654	2.609
11	4	111.75	50	0.432	199.23	9.962	2.673
12	4	109.48	50	0.431	198.20	9.910	2.714
13	5	107.85	50	0.472	244.53	12.227	3.399
14	5	108.99	50	0.470	242.08	12.104	3.330
15	5	110.45	50	0.497	277.11	13.855	3.761
16	6	81.69	50	0.500	281.26	14.063	5.162
17	6	102.82	50	0.493	271.66	13.583	3.961
18	6	103.95	50	0.519	308.87	15.444	4.455
19	7	110.29	50	0.454	223.19	11.159	3.034
20	7	111.75	50	0.476	249.51	12.476	3.347
21	7	111.92	50	0.449	217.54	10.877	2.914
22	8	110.29	50	0.556	369.57	18.479	5.024
23	8	111.27	50	0.545	350.52	17.526	4.723
24	8	108.67	50	0.598	450.85	22.543	6.220
25	9	113.70	50	0.478	252.03	12.602	3.323
26	9	113.70	50	0.470	242.08	12.104	3.192
27	9	112.89	50	0.465	236.03	11.802	3.135
28	10	108.02	50	0.470	242.08	12.104	3.360
29	10	104.77	50	0.482	257.14	12.857	3.680
30	10	111.27	50	0.490	267.63	13.381	3.606
